# *In vivo* protein interaction network analysis reveals porin-localized antibiotic inactivation in *Acinetobacter baumannii* strain AB5075

**DOI:** 10.1038/ncomms13414

**Published:** 2016-11-11

**Authors:** Xia Wu, Juan D. Chavez, Devin K. Schweppe, Chunxiang Zheng, Chad R. Weisbrod, Jimmy K. Eng, Ananya Murali, Samuel A. Lee, Elizabeth Ramage, Larry A. Gallagher, Hemantha D. Kulasekara, Mauna E. Edrozo, Cassandra N. Kamischke, Mitchell J. Brittnacher, Samuel I. Miller, Pradeep K. Singh, Colin Manoil, James E. Bruce

**Affiliations:** 1 Department of Genome Sciences, University of Washington, 850 Republican Street, Brotman Building Room 154, Seattle, Washington 98109, USA; 2 Department of Chemistry, University of Washington, Seattle, Washington 98109, USA; 3 Department of Microbiology, University of Washington, Seattle, Washington 98195, USA; 4 Department of Medicine, University of Washington, Seattle, Washington 98195, USA

## Abstract

The nosocomial pathogen *Acinetobacter baumannii* is a frequent cause of hospital-acquired infections worldwide and is a challenge for treatment due to its evolved resistance to antibiotics, including carbapenems. Here, to gain insight on *A. baumannii* antibiotic resistance mechanisms, we analyse the protein interaction network of a multidrug-resistant *A. baumannii* clinical strain (AB5075). Using *in vivo* chemical cross-linking and mass spectrometry, we identify 2,068 non-redundant cross-linked peptide pairs containing 245 intra- and 398 inter-molecular interactions. Outer membrane proteins OmpA and YiaD, and carbapenemase Oxa-23 are hubs of the identified interaction network. Eighteen novel interactors of Oxa-23 are identified. Interactions of Oxa-23 with outer membrane porins OmpA and CarO are verified with co-immunoprecipitation analysis. Furthermore, transposon mutagenesis of *oxa-23* or interactors of Oxa-23 demonstrates changes in meropenem or imipenem sensitivity in strain AB5075. These results provide a view of porin-localized antibiotic inactivation and increase understanding of bacterial antibiotic resistance mechanisms.

## Abstract (web summary)

The bacterial pathogen *Acinetobacter baumannii* has evolved resistance to many antibiotics, including carbapenems. Here, Wu *et al.* show that the carbapenemase Oxa-23 interacts with the outer membrane porin CarO in an *A. baumannii* isolate, indicative of porin-localised antibiotic inactivation.

## Graphical abstract

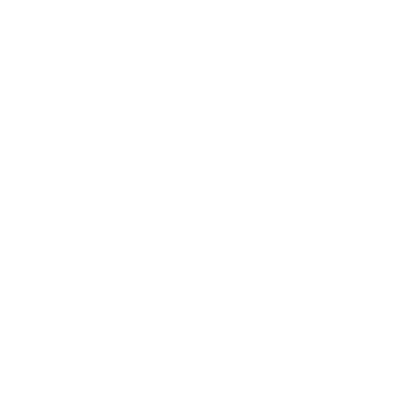

A*cinetobacter baumannii* is a Gram-negative nosocomial pathogen that has emerged as a common threat for hospital acquired infections worldwide^1^. In a prevalence study of infections in intensive care units among 75 countries, *Acinetobacter* species were observed to be the fifth most common pathogen and *Acinetobacter* infections were also associated with greater risk of hospital mortality^2^. Two notable characteristics of *A. baumannii* pathogens were reported: (1) *A. baumannii* has gradually evolved resistance to many antibiotics (that is, multidrug resistance), including carbapenems, and (2) *A. baumannii* is inherently resistant to desiccation and disinfectants, and therefore difficult to eradicate^3^. Furthermore, data acquired from 55,330 US clinical *A. baumannii* isolates from 2002 to 2008 indicate that carbapenem-associated multiclass resistance in *A. baumannii* increased from 20.6% in 2002 to 49.2% in 2008 (ref. 4). As a result, recent years have seen an increase in patient morbidity, mortality and health care costs attributed to *A. baumannii* infections^5^,^6^. *A. baumannii* AB5075 is a multidrug-resistant and infectious clinical strain acquired from a patient with tibia osteomyelitis^7^,^8^ and is the focus in the present study.

Significant efforts have already been invested to better understand multidrug resistance mechanisms in *A. baumannii*. These results revealed that β-lactamases and membrane porins are major genetic determinants of *A. baumannii* drug resistance^3^,^9^. β-Lactamase enzymes represent a class of unique transpeptidases that possess hydrolytic activity against β-lactam antibiotics. In *A. baumannii*, β-lactamases have evolved over time with enhanced catalytic ability against a wider spectrum of β-lactams including last-resort carbapenems^10^,^11^. Specifically, the class-D β-lactamases (also called oxacillinases) Oxa-51 and Oxa-23 are most frequently linked to *A. baumannii* multidrug-resistant phenotypes^12^,^13^.

Membrane porins, on the other hand, are mainly responsible for transport of nutrients and antibiotics into bacterial cells^14^. The membrane permeability and porin activity in *A. baumannii* are intrinsically low compared with *Escherichia coli*^15^,^16^, consistent with the hypothesis that limited antibiotic penetration may contribute to the multidrug resistance in *A. baumannii* cells^17^. Notably, inactivation of a main membrane porin CarO causes increased resistance to carbapenems in *A. baumannii* strains (AB242, AB825, AB125 and AB17978)^18^,^19^,^20^. Likewise, inactivation of a 33 KDa outer membrane (OM) porin was associated with increased carbapenem resistance in *A. baumannii* strain JC10/01 (ref. 21).

Despite current progress, many details regarding antibiotic resistance mechanisms in *A. baumannii* are still lacking. For example, in AB5075, both *oxa-23* and *carO* genes are encoded in the genome, whose expression levels are known to exert opposing effects to antibiotic resistance^9^. However, as mentioned, AB5075 is multidrug resistant and such traits cannot be fully explained by the presence/absence of *oxa-23* and *carO* genes. One reason may be that *oxa-23* and *carO* genes have genetic interactions when both are present. Another reason could be that additional factors that are not yet identified also contribute to drug resistance in AB5075. This is particularly relevant when considering that ∼30% of the genes in *A. baumannii* strains (1,122/3,732 in AB0057, 1,326/3,656 in AB_AYE and 1,074/3,813 in AB5075) are currently annotated as uncharacterized proteins. Thus, we decided to investigate the protein interaction network in AB5075 cells, first to identify protein interactions for protein candidates that are known to be important for antibiotic resistance (including Oxa-23 and CarO) and, second, to identify novel protein targets that may be associated with antibiotic resistance in AB5075 cells based on the protein interaction data.

*In vivo* chemical cross-linking with mass spectrometry (XL-MS) was used for these efforts, because this approach can generate both protein–protein interaction and cross-link site information. As chemical cross-linking requires the two reactive amino acids are close to one another, the cross-link site information uniquely provides structural details on identified protein interactions. The *in vivo* cross-linking approach employed here is based on protein interaction reporter (PIR) technology first developed in our lab^22^. PIR technologies combine amine reactive groups, MS-cleavable features and affinity tag for enrichment. Peptide pairs linked by PIR cross-linkers are released after low-energy collision-induced dissociation in the mass spectrometer and each peptide is subjected to independent fragmentation by the targeted MS^3^ to obtain peptide sequence and protein interaction information. Importantly, PIR cross-linkers are cell membrane permeable, enabling *in vivo* analysis of protein complexes including membrane and soluble proteins in bacterial and human cells^23^,^24^,^25^,^26^,^27^,^28^,^29^. Other groups have also applied XL-MS, to determine protein surfaces, subunit localization and orientations, binding interface and dynamics of protein complexes^30^,^31^,^32^,^33^,^34^,^35^.

In the present study, the PIR XL-MS method is further optimized for the *in vivo* analysis of protein interactions in the multidrug-resistant clinical isolate *A. baumannii* AB5075. By automatically targeting cross-linked peptides ‘on the fly'^26^, we identify an extensive protein interaction network in AB5075, including Oxa23-CarO and Oxa23-OmpA complexes that were previously unknown. The cross-link sites also provide the first structural insights for these complexes in AB5075 cells.

## Results

### *In vivo* protein interaction network of *A. baumannii* AB5075

**Figure 1:**
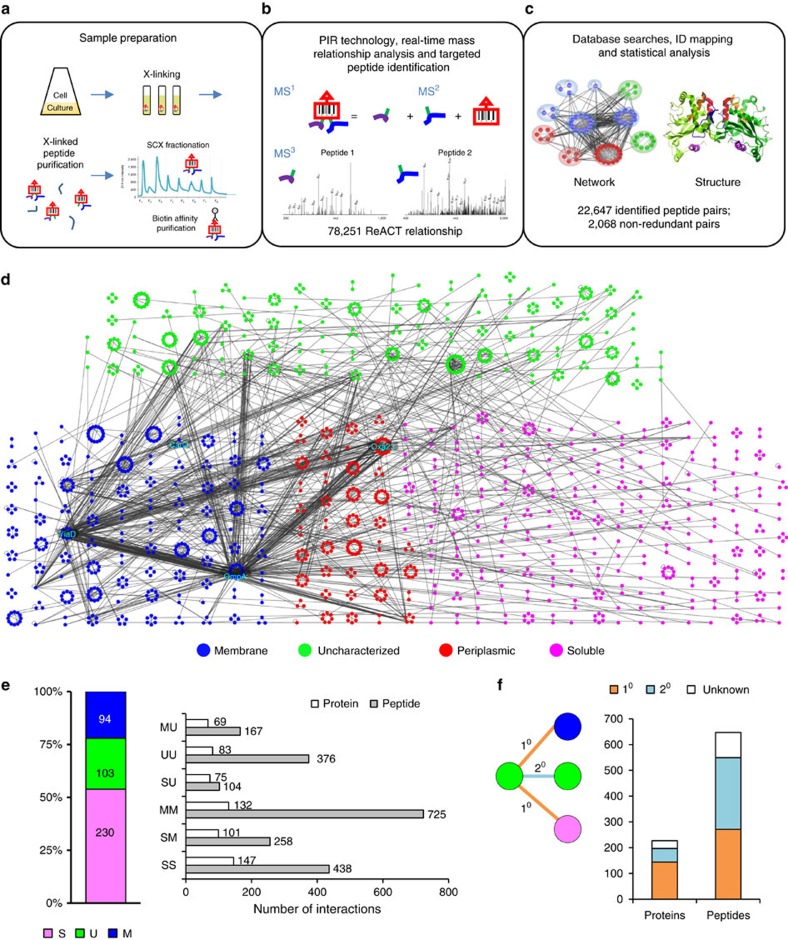
Identification of *in vivo* protein interactions in *A. baumannii* cells. (**a**–**c**) Schematic overview of experimental procedures. (**d**) Protein interaction network in AB5075 cells comprising 2,068 non-redundant cross-linked peptide pairs. Nodes indicate the identified cross-linked sites and were grouped into circles to represent proteins. Node colours illustrate the protein primary subcellular localization. Blue, membrane proteins; green, uncharacterized proteins; magenta, soluble proteins; red, soluble proteins predicted to be localized in periplasm^47^,^48^. Edges represent the identified cross-links between the two sites. Protein interaction hubs OmpA, YiaD and Oxa-23, as well as OM porin CarO are highlighted. Uncharacterized proteins were identified cross-linked to membrane, periplasmic and soluble proteins. (**e**) Composition of protein nodes (M, membrane proteins; S, soluble proteins; U, uncharacterized proteins) and edges in the network based on subcellular localization. Edges: MM, cross-links between membrane proteins; MU, cross-links of membrane proteins to uncharacterized proteins; SM, cross-links of membrane proteins to soluble proteins; SS, cross-links between soluble proteins; SU, cross-links of soluble proteins to uncharacterized proteins; UU, cross-links between uncharacterized proteins. (**f**) Cross-links between the uncharacterized proteins and the functionally annotated proteins, either directly (1° interactions) or through an intermediate partner (2° interactions), could shed light on the function for these uncharacterized proteins.

PIR cross-linking caused no observable loss in live cells for AB5075 (Supplementary Fig. 1). A total of 2,068 non-redundant cross-linked peptide pairs were identified in AB5075 with the *in vivo* cross-linking approach (Fig. 1a–c and Supplementary Fig. 2). Among those, 1,148 cross-links correspond to 245 intra-molecular interactions (that is, two cross-linked peptides are from the same protein molecule) and the remaining 920 cross-links represent 398 inter-molecular interactions (including 349 binary protein interactions and 49 homooligomers; that is, two cross-linked peptides are from different protein molecules). The experimental reproducibility of the identified cross-links was about 55–80% for inter-data set comparison, consistent with repeatability levels common to data-dependent acquisition in proteomics experiments^36^. Of note, OM proteins OmpA and YiaD, as well as β-lactamase Oxa-23, were the three proteins most frequently identified in these experiments and exhibited the most dense edges in the protein interaction network (Fig. 1d).

A subset of the data set comprised homodimeric cross-linked peptide pairs (that is, the two cross-linked peptides have identical or overlapping amino acid sequences from the same protein)^24^,^29^. Forty-nine homomultimeric interactions were identified, supported with 101 unambiguous homodimeric cross-links. This includes several known homooligomeric proteins, such as glyceraldehyde-3-phosphate dehydrogenase (GapA), citrate synthase (GltA) and OM protein OmpA^24^,^37^, whereas many other homodimeric interactions have not been previously reported (Supplementary Data 1). Particularly relevant to *A. baumannii* multidrug resistance, unambiguous homodimer linkages were identified in resistance-nodulation-division efflux pump components AdeI, AdeK, AcrB and the key β-lactamase Oxa-23 discussed in detail below.

We categorized the cross-linked proteins based on their annotated primary subcellular localization^38^,^39^, which included 94 membrane proteins (M), 230 soluble proteins (S) and 103 uncharacterized proteins (U). The analysis revealed that each protein sub-group was linked more frequently to proteins of the same classification (that is, M–M, S–S and U–U; Fig. 1e), although inter-class linkages were also identified. PIR technology enabled identification of interactions between soluble and membrane proteins, which are difficult to detect by conventional methods. Novel linkages of uncharacterized proteins to the annotated proteins were also identified, which helped reveal the functional context of those uncharacterized proteins (Fig. 1d,f). Membrane protein complexes (M–M) constituted the highest number (725) of cross-linked peptide pairs, averaging 5 links for each given protein pair, and provides the greatest detail that can be used for structural mapping of protein interactions. For instance, although identified S–S interactions constituted a slightly larger number of protein interactions (147 versus 132 M–M), the number of cross-linked S–S peptide pairs identified was only 60% of that found for M–M. The greater cross-linking efficiency for membrane proteins in these experiments may be attributed to the greater local concentrations of the cross-linkers exposed to membrane proteins compared with cytosolic proteins. The cross-linking reactions were performed in this study with intact cells and membrane proteins are localized in outer layers of subcellular compartments and thus may experience highest cross-linker concentration.

To further characterize the identified protein interactions, we performed sequence alignment of all cross-linked proteins to the protein domain database SUPERFAMILY 1.75 (ref. 40). The precise cross-linked residues were used to determine what parts of the proteins (at the domain level) are most often identified in cross-linked relationships and involved in protein–protein interactions. As a result, cross-linked sites of 225 proteins were identified within recognized domain sequences (*E*-value ≤1.0E−4). Moreover, when proteins shared a common domain, cross-linked sites from these proteins were observed to be in the conserved domains. For example, the identified cross-linked sites in OmpA-like amino-terminal (N-terminal) domain consist of cross-link data of three β-barrel porins, including OmpA, OmpW and CarO. Likewise, cross-linked sites in OmpA-like carboxy-terminal (C-terminal) domain were attributed to five cross-linked proteins that contain OmpA-like C-terminal domains, including OmpA, YiaD, Pal, ABUW_0505 and ABUW_2730. The results gave rise to a domain interaction network of 74 inter-domain and 98 intra-domain linkages (Supplementary Fig. 3 and Supplementary Data 2). Six of the inter-domain and 55 of the intra-domain interactions were common to previous identified interaction domains in *E. coli*^26^ or *Pseudomonas aeruginosa*^27^. At the protein level, homologous proteins involved in 138 inter-protein interactions (39.5% of the 349) and 168 intra-protein interactions (68.6% of the 245) in AB5075 could be mapped to the *E. coli* Interaction Database^41^, using a protein BLAST score threshold (bit score ≥40). The domain interaction network revealed that OmpA-like C-terminal, OmpA-like N-terminal and β-lactamase/transpeptidase-like domains are frequently involved in inter-domain interactions, underlying the sequence and structural basis of OmpA, YiaD and Oxa-23 proteins being the hubs in protein interactions in AB5075, in these experiments.

It is noted that although OmpA, YiaD and Oxa-23 are abundant proteins in AB5075, no strong correlation was found between protein abundance^42^ and the number of identified protein interactions for a given protein (*R*^2^<0.3; Supplementary Fig. 4).

### Protein interactions of β-lactamase Oxa-23 in AB5075 cells

As Oxa-23 is a primary antibiotic resistance determinant in *A. baumannii* strains^9^,^10^ and Oxa-23 appears to be an interacting ‘hub' in AB5075 in the network above (Fig. 1d), a focus on Oxa-23 interactions is presented here.

Oxa-23 is one of the six β-lactamase-like domain containing proteins identified in the cross-linking analysis in AB5075 cells. The other five identified β-lactamase-like proteins also included class D β-lactamase Oxa-69, class C β-lactamase AmpC, class A β-lactamase blaGES-11 and two penicillin-binding proteins PBP-1A (ABUW_0289) and PBP-6 (ABUW_1127). The crystal structure of Oxa-23 in *A. baumannii* (PDB 4JF6)^43^ consists of a globular α-domain and a β-sheet-rich β-domain (Supplementary Fig. 5A), which is a general structural feature conserved for β-lactamases. Four unambiguous homodimeric cross-links of Oxa-23 were identified (K194, K102, K104 and K107 sites), indicating that Oxa-23 proteins could form homodimers as reported for other class D β-lactamases^44^,^45^. Superimposition of the Oxa-23 monomeric structures with the dimeric templates of Oxa-10 (ref. 44) and Oxa-46 (ref. 45) showed that the K194-K194 cross-link (located at α8 helix) in the dimeric model matched the distance constraints for the biotin aspartyl prolyl *N*-hydroxyphthalamide (BDP-NHP) cross-linkers, whereas K102-K102, K104-K107 and K107-K107 cross-links (all located at α3-α4 loop) did not (Supplementary Fig. 5B). The identification of homodimeric cross-links at α3-α4 loop of Oxa-23 structure suggests the dynamic conformations of Oxa-23 dimers or the existence of higher orders of oligomeric forms of Oxa-23 in AB5075. It is noted that a tetrameric form of Oxa-10 was previously reported^46^.

**Figure 2:**
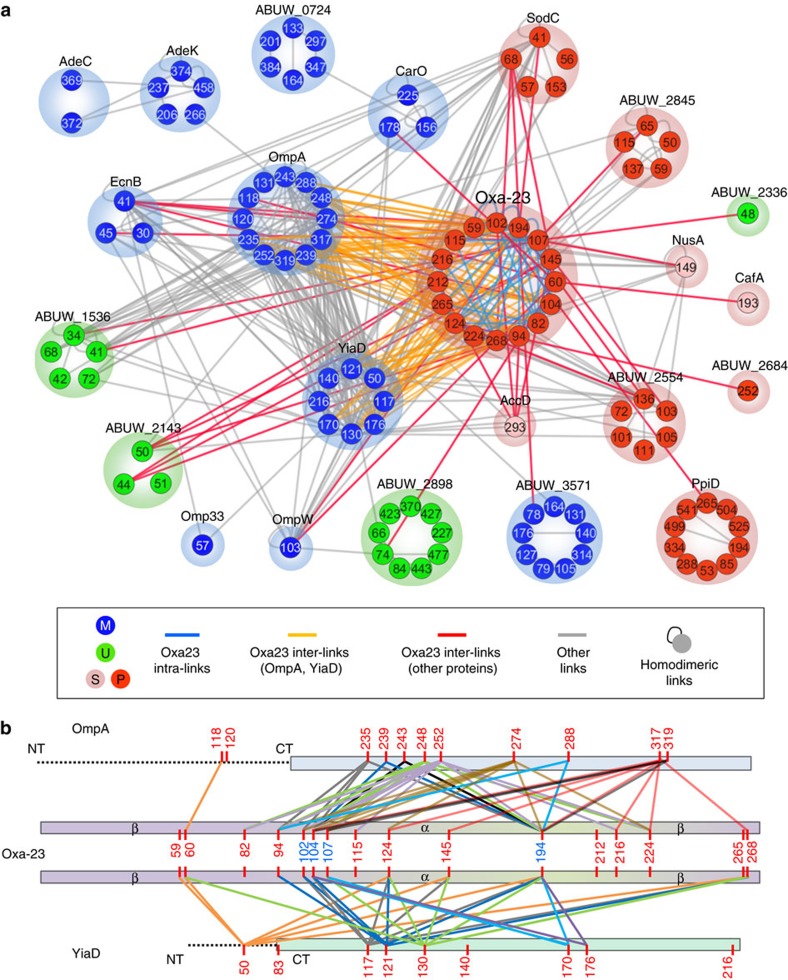
Protein interaction network of Oxa-23 in AB5075. (**a**) The network included 18 Oxa-23 direct interaction partners (6 membrane, 4 uncharacterized and 8 soluble proteins) and four extended interaction partners (Omp33, AdeK, AdeC and ABUW_0724). Node number indicates cross-linked lysine residues. Nodes were grouped by proteins. Node colour shows the primary subcellular localization of the proteins. Blue, membrane proteins; green, uncharacterized proteins; light red, soluble proteins; dark red, soluble proteins predicted to localize in periplasm. Edge colours illustrate the specific types of Oxa-23 cross-links. Blue edges are intra-protein cross-links of Oxa-23. Orange or red edges are inter-protein cross-links of Oxa-23 to OmpA and YiaD, or to other interactors. Grey edges are cross-links formed between Oxa-23 interactors. The network was generated with Cytoscape 3.0.2 (ref. 73). (**b**) Inter-protein cross-links of OmpA-Oxa23 and YiaD-Oxa23 annotated with protein primary sequences and protein domains. Homodimeric cross-linked sites of Oxa-23 K102, K104, K107 and K194 are highlighted in blue. Cross-links are colored based on the cross-linked lysine residues of OmpA and YiaD.

Eighteen protein interactors were identified cross-linked to Oxa-23 in AB5075 (Fig. 2a). These interactors were enriched for the presence of predicted signal peptides^47^,^48^ (*P*=0.00017, Fisher's exact test), consistent with the periplasmic localization of Oxa-23 proteins. In particular, OM proteins OmpA (with 53 non-redundant links) and YiaD (with 31 non-redundant links) were most heavily linked to Oxa-23. Mapping of the cross-linked residues to protein primary sequences showed that the majority of the cross-links were located at the C-terminal domains of OmpA and YiaD proteins (Fig. 2b). Interestingly, both of the C-terminal domains of OmpA and YiaD shared a conserved OmpA-like C-terminal domain structure (Supplementary Fig. 6) that was reported to associate with peptidoglycan layer in bacterial cells^49^. The diversity of Oxa-23 cross-links with OmpA and YiaD C-terminal domains suggests the dynamic associations of Oxa-23 with OmpA and YiaD. In addition to binding to OmpA and YiaD, Oxa-23 was also observed to interact with other OM proteins, including CarO porin, OmpW porin and lipoprotein ABUW_3571 (Fig. 2a). Gene Ontology analysis showed that Oxa-23 interactors function in multiple biological pathways. For instance, Oxa-23 interacts with metabolic enzymes including superoxide dismutase SodC, pathogenesis-related proteins including entericidin EcnB, two haemolysin-like proteins (ABUW_2554 and ABUW_2845) and four uncharacterized proteins including ABUW_1536, ABUW_2143, ABUW_2336 and ABUW_2898. Phyre2 homology modelling^50^ further identified that ABUW_2898 contains an 18-stranded β-barrel structure homologous to capsule protein Wzi (PDB 2YNK) in *E. coli*^51^.

### Membrane porin localization of Oxa-23 in AB5075

**Figure 3:**
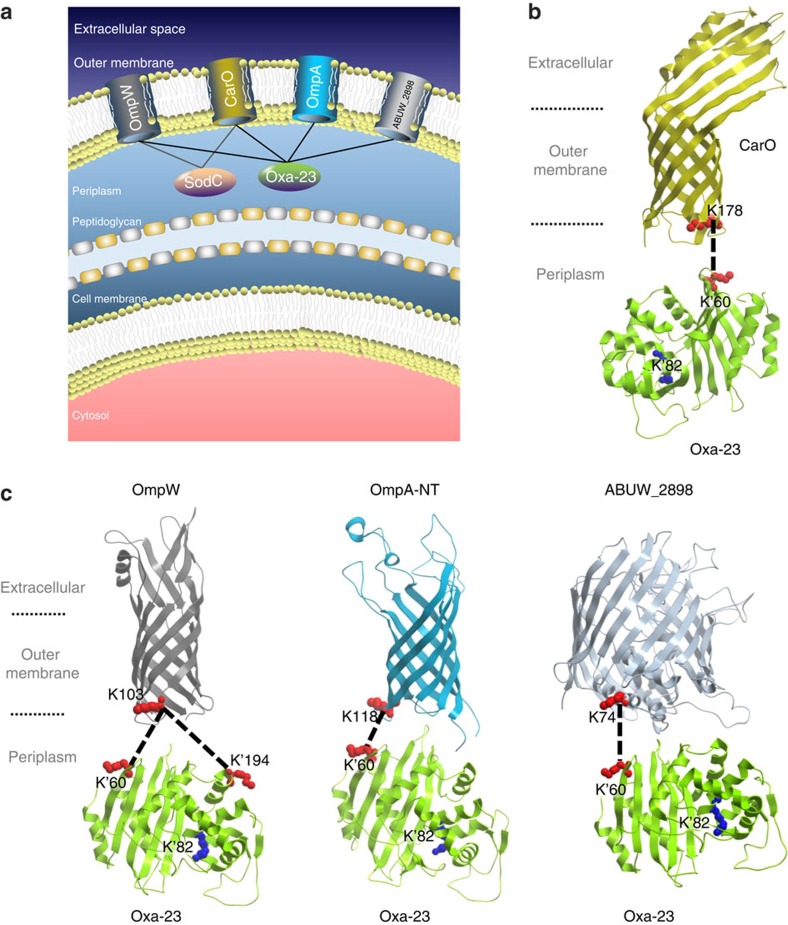
Protein interactions of Oxa-23 with outer membrane porins. (**a**) Summary model showing interactions of periplasmic enzymes Oxa-23 and superoxide dismutase SodC with the OM porins that may help localize Oxa-23 and SodC near the inner surface of the OM. (**b**) Structural features of Oxa-23 interactions with the eight-stranded β-barrel of CarO porin. PDB structures Oxa-23 (4FJ6)^43^ and CarO (4FUV)^60^ were used. (**c**) Structural features of Oxa-23 interactions with OmpW, OmpA-NT and ABUW_2898. Structural models of the 8 stranded β-barrel of OmpW, OmpA-NT and the 18-stranded β-barrel of ABUW_2898 were generated with Phyre2 (ref. 50) using *E. coli* templates OmpW (2F1T)^74^, OmpA-NT (1G90)^75^ and Wzi (2YNK)^51^. It is noteworthy that OmpA has also been proposed to fold into a one-domain, open-channel conformation^15^,^76^. However, structural models of the open-channel forms are not available. The subcellular compartments, the identified cross-link sites and Oxa-23 catalytic site K82 are highlighted.

Oxa-23 was identified interacting with four membrane porins, including OmpA-N-terminal domain, CarO, OmpW and ABUW_2898 (Fig. 3a). Strikingly, we found that all porins were cross-linked to a common residue K60 in Oxa-23, which is localized in the β-strand loop β1–β2 in Oxa-23 structure (Fig. 3b,c). Besides K60, only one other site in Oxa-23 was identified as cross-linked to any porin, which was K194 that was cross-linked to OmpW-K103. We verified that both K60 and K194 are solvent accessible residues^52^. Peptides containing Oxa23-K60 residue (KINLYGNALSR) were also identified in intra-protein cross-links with other Oxa-23 peptides. We examined the K60 peptide fragment spectra in these intra- and inter-protein cross-links, and observed strong spectral similarities. Thus, the general, low false discovery rate (FDR) of an intra-protein cross-link in database searches and the spectrum correlation of K60 peptide in inter- and intra-protein cross-link identification insured beyond the applied FDR cutoff that the peptide sequence assignment of Oxa-23 K60 peptide in Oxa23-porin cross-links was correct. Intriguingly, lysine residues in porin proteins that were identified cross-linked to Oxa-23 K60 were all localized at the periplasmic end of the β-barrel structures (CarO: K178, OmpW: K103, OmpA: K118 and ABUW_2898: K74; Fig. 3b,c). As the β-barrel structures are orientation specific when inserted in OMs, these site-specific cross-links between Oxa-23 and porin structures suggested a protein conformation-specific binding of Oxa-23 to porins at the periplasmic side of the OM of the cell. As carbapenem mechanism of action involves inhibition of peptidoglycan synthesis^53^, localizing Oxa-23 outside the peptidoglycan layer, nearest to the OM where carbapenems must first pass, before diffusion to their intended targets could serve to protect critical peptidoglycan synthetic enzymes.

**Figure 4:**
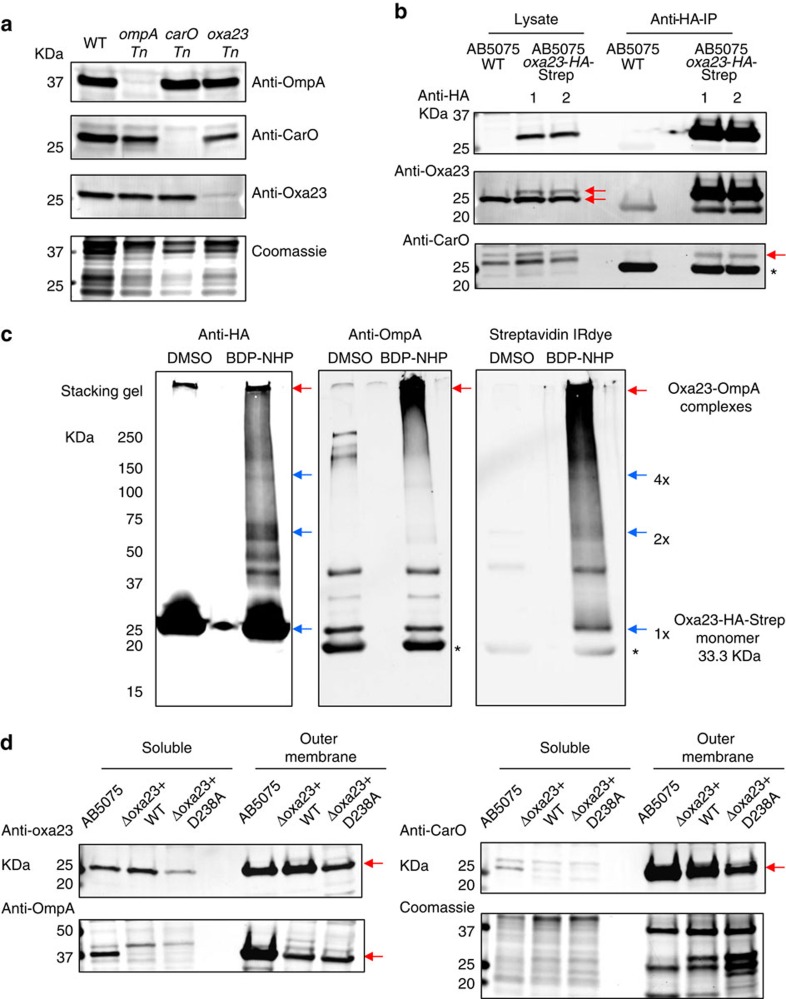
Validation of Oxa-23 interactions with CarO and OmpA. (**a**) Antibody specificity was confirmed with detection of protein bands that matched the molecular weight of the target proteins (OmpA, CarO and Oxa-23). Furthermore, the detected protein bands also matched the sizes of protein bands that showed reduction in specific transposon mutants (*ompA Tn*, *carO Tn* and *oxa-23 Tn*). (**b**) Co-IP analysis of anti-HA antibodies with AB5075-pMMB-*oxa23* cell protein lysates. Red arrows in anti-Oxa23 immunoblots indicate the detection of both the endogenous Oxa-23 and the tagged Oxa-23. Red arrow in anti-CarO immunoblots indicates CarO proteins that were co-immunoprecipitated with Oxa23-HA-Strep in AB5075. Asterisk corresponds to the immunoglobulin light chains. (**c**) Co-IP analysis of anti-HA antibodies with the BDP-NHP cross-linked AB5075-pMMB-*oxa23* cells. Blue arrows indicate that protein bands matching the sizes of Oxa23-HA-Strep monomers (1 × ), dimers (2 × ) and tetramers (4 × ) could be immunoprecipitated with anti-HA antibodies after the BDP-NHP cross-linking reactions. Red arrows show that higher molecular weight bands reactive to anti-OmpA antibodies could be pulled down with the anti-HA antibodies, supporting the identification of Oxa23-OmpA protein interactions. Streptavidin IRdye confirmed that the high molecular weight bands contained strong biotin (that is, cross-linking) signals in the cross-linked samples, but not in the dimethyl sulfoxide (DMSO)-treated control samples. Asterisks correspond to the immunoglobulin light chains. (**d**) OM protein-enriched fractions were prepared from AB5075 WT, or *Δoxa23* complementation mutants with pMMB.A1-*oxa23* (−WT or −D238A) cells. Two micrograms of protein were loaded for each lane. Strong anti-Oxa23 immunoblot signals were observed at the OM-enriched fractions. Enrichment of OM-protein markers OmpA and CarO are also shown in these samples. Red arrows indicate the detection of specific protein bands, Oxa-23, OmpA and CarO. Coomassie-stained gel shows the loading of equal total proteins. Full blots are shown in Supplementary Fig. 15.

To verify the identified Oxa-23 interactions with membrane porins, we obtained the sequence-specific antibodies for OmpA, CarO and Oxa-23 proteins (Methods). The specificity of the antibodies was confirmed with the reactivity of protein bands that matched the expected molecular weights of target proteins (OmpA, CarO or Oxa-23) and the reduction of those protein bands in specific transposon mutants (*ompA Tn*, *carO Tn* and or *oxa-23 Tn*)^54^ (Fig. 4a).

AB5075-pMMB-*oxa23* cells that expressed Oxa-23 with C-terminal HA-Strep tags (Supplementary Methods) were used for co-immunoprecipitation assays (co-IP), to examine the binding of Oxa23-HA-Strep with OmpA and CarO *in vivo*. As shown in Fig. 4b, anti-HA and anti-Oxa23 antibodies confirmed that Oxa23-HA-Strep proteins could be expressed in AB5075 cells and could be immunoprecipitated with anti-HA agarose antibodies. Furthermore, anti-CarO antibodies detected CarO protein bands that could be co-immunoprecipitated with Oxa23-HA-Strep proteins, consistent with the XL-MS identification of CarO-Oxa23 proteins in AB5075. However, the co-IP evidence for OmpA-Oxa23-HA-Strep was not observed under native co-IP conditions, possibly due to the inaccessibility of HA tag of Oxa23-HA-Strep proteins that were bound to OmpA.

To further verify the interactions of OmpA-Oxa23 in AB5075 cells, we cross-linked AB5075-pMMB-*oxa23* cells. Protein lysates were denatured with SDS detergents to expose the HA-Strep tags of OmpA-Oxa23 complexes and anti-HA co-IP analysis was performed with the denatured protein lysates. As shown in Fig. 4c, anti-HA agarose antibodies pulled down both the non-cross-linked and cross-linked forms of Oxa23-HA-Strep proteins. Furthermore, anti-HA and anti-OmpA antibodies detected the overlapping cross-linked protein bands at high molecular weights (>250 kDa) that contained OmpA-Oxa23-HA-Strep complexes. The large size of OmpA-Oxa23 complexes is consistent with the observation of OmpA and Oxa-23 protein-interaction hubs in AB5075, in which the purified OmpA-Oxa23 complexes could also be cross-linked to other proteins. In addition, the cross-linking co-IP assays also revealed the Oxa23-HA-Strep protein bands matching the dimeric and tetrameric sizes of the Oxa23-HA-Strep proteins, consistent with the identification of homoodimeric cross-links of Oxa-23 in AB5075 cells.

Another line of evidence includes the strong anti-Oxa23 signals observed in the OM protein-enriched fractions based on differential centrifugation analyses. Enrichment of OM proteins was confirmed with the OM protein markers OmpA and CarO (Fig. 4d). The co-purification of Oxa-23 with OmpA and CarO proteins in OMs supports the XL-MS identification of Oxa-23 interactions with OM porins in AB5075 cells. It is noteworthy that the OM association of Oxa-23 was also verified for the complemented Oxa-23 proteins with pMMB.A1 plasmid in *Δoxa23* deletion strains (details of *Δoxa23* complementation mutants are further described below).

### Antibiotic susceptibility of *oxa-23* transposon mutants

To determine the effects of Oxa-23 protein interactions on antibiotic resistance, transposon insertion mutants for *oxa-23* or genes encoding Oxa-23 interactors were obtained in the background strain of AB5075 (ref. 54). Minimal inhibitory concentration (MIC) assays were used to examine the antibiotic sensitivity of *A. baumannii* mutants. We confirmed that AB5075 wild-type (WT) strain is carbapenem resistant, with MIC of 8 μg ml^−1^ for both meropenem and imipenem (Table 1). In contrast, the MICs for the antibiotic-sensitive strain AB19606 were only 1 μg ml^−1^ for meropenem and 0.5 μg ml^−1^ for imipenem^55^. Inactivation of *oxa-23* caused twofold increase in sensitivity to meropenem and eightfold increase to imipenem, indicating that *oxa-23* is one of the major genetic determinants for carbapenem resistance in AB5075. In comparison, inactivation of other β-lactamase genes *oxa-69* and *ampC* did not increase antibiotic sensitivity, consistent with the argument that Oxa-69 and AmpC lack enzyme specificity and/or protein interactions to produce resistance effects.

Five OM porins were identified in the extended interaction network of Oxa-23 (Fig. 2a) and all of these five porins showed measureable changes in meropenem sensitivity, and two of them also showed detectable changes to imipenem sensitivity (Table 1). Interestingly, the trend of these sensitivity changes appeared to be porin specific, which could be attributed to the specificity in antibiotic uptake and transport for the porin channels^55^. We observed that the *carO* transposon mutant became slightly more sensitive to meropenem and imipenem compared with AB5075 WT, which is opposite to previous reports for other *A. baumannii* strains (AB242, AB825, AB125 and AB17978)^18^,^19^,^20^. This strain-specific phenotype may be owing to emerging functional roles of CarO in AB5075 strain, where it serves not only as a membrane channel but also as an anchor to localize Oxa-23 to the OM. Similarly, we observed a meropenem- and imipenem-sensitive phenotype when inactivating another major porin, OmpA in AB5075. On the other hand, transposon mutants for membrane porin *ompW* and an uncharacterized porin *ABUW_0724* (cross-linked to CarO) exhibited twofold increased resistance to meropenem, although not for imipenem. The results for *ompW* mutants are consistent with the observation of decreased levels of OmpW in carbapenem-resistant strain AB_RS307 (ref. 56). Finally, inactivation of *yiaD* (an Oxa-23 major interacting partner and OmpA-like-CT domain-containing protein) in AB5075 increased resistance to meropenem, while slightly decreasing the resistance to imipenem.

**Figure 5:**
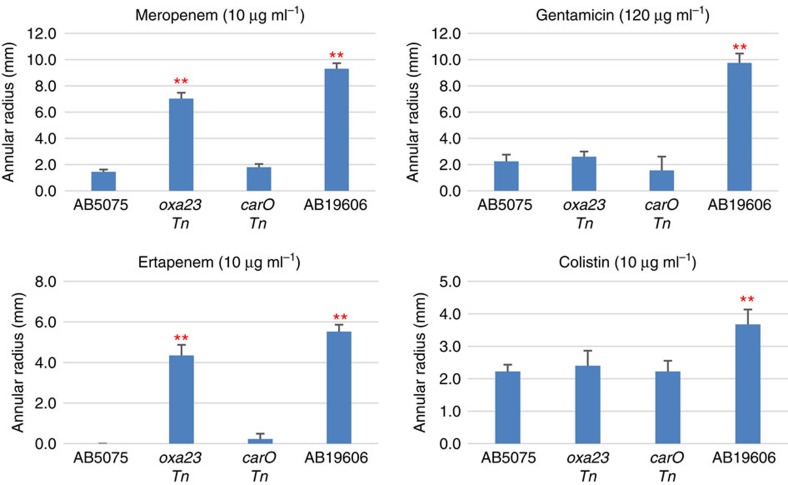
Antibiotic sensitivity of AB5075 cells and transposon mutants examined with agar diffusion assays. The inhibition zone (annular radius) was measured after incubation at 37 °C for 24 h. Four individual isolates (biological replicates) were examined for each strain. Error bars indicate the s.d. of the four biological replicates. Asterisks indicate statistically significantly different from AB5075 WT (**P<0.01, one-way unpaired analysis of variance, *n*=4). Meropenem and imipenem sensitivity measurements with MIC assays are shown in Table 1.

Consistent with results of MIC assays, agar diffusion assays showed that AB5075 is more resistant to carbapenem antibiotics compared with AB19606, including meropenem and ertapenem. Moreover, AB5075 is also more resistant than AB19606 to the aminoglycoside antibiotic gentamicin and the polymyxin antibiotic colistin (Fig. 5). Meanwhile, *oxa-23 Tn* mutant exhibited increased sensitivity to carbapenems, but not to gentamicin and colistin compared with AB5075 WT. This antibiotic class-specific response in *oxa-23 Tn* mutant is consistent with the enzymatic specificity of Oxa-23 (refs 43, 57). Furthermore, these results also suggest that although Oxa-23 is important in carbapenem resistance, there may be other resistance mechanisms in AB5075, making it more resistant than AB19606 to gentamycin and colistin.

### *Δoxa23* deletion mutant and complementation mutants

To further investigate the role of Oxa-23 in carbapenem resistance in AB5075, a *Δoxa23* gene deletion mutant was obtained. The *Δoxa23* mutant exhibited 8-fold increase in meropenem sensitivity and 16-fold increase in imipenem sensitivity compared with AB5075 WT (Table 2). The greater sensitivity changes for the *Δoxa23* mutant relative to the *oxa-23* transposon mutant may be attributed to the more complete deletion of *oxa-23* in the *Δoxa23* mutant. We next complemented the *Δoxa23* mutant with pMMB.A1 vector, which enables constitutive expression of *oxa-23*. The Oxa-23 protein level in *Δoxa23+*pMMB.A1-*oxa23*-WT complementation mutant was about 1.5-fold of the original amount in AB5075 WT. In this condition, carbapenem resistance was regained in the complementation mutant (Table 2). Deletion of Oxa-23 proteins appeared to also have an impact on the OmpA and CarO protein levels (Supplementary Fig. 7). Nonetheless, changes of OmpA and CarO protein abundance did not appear to affect the outcome of Oxa-23 complementation strain to regain carbapenem resistance. These results indicate that *oxa-23* is critical to confer carbapenem resistance in AB5075.

We analysed the electrostatic potential surfaces in three-dimensional structures of Oxa-23 and the four OM porins. Charged residues 58DKK60 (at the β1–β2 loop) and D238 (at the β7–β8 loop) of Oxa-23 were identified as residues that could potentially form complementary electrostatic interactions with charged patches in porin periplasmic loops (Supplementary Fig. 8). *In silico* analyses with SCWRL4 (ref. 58) and site-directed mutator^59^ predicted that mutations of 58DKK60AAA and D238A did not result in significant structural changes for Oxa-23 proteins (Supplementary Fig. 9). Carbapenem hydrolysis activity assays with *E. coli* lysate containing recombinant Oxa-23 proteins verified that mutants 58DKK60AAA and D238A did not exhibit decreased activity compared with WT Oxa-23 proteins. However, when mutations were combined to a 58DKK60AAA/D238A double mutant, the meropenem hydrolysis activity of the mutant decreased by ∼30% compared with WT Oxa-23, whereas the imipenem hydrolysis activity of the mutant remained intact Supplementary Fig. 10). Thus, the mutations imparted in β-loop regions of Oxa-23 caused minor change in meropenem and no discernable change in imipenem hydrolysis activity of the enzyme as observed in *E. coli* cell lysates, consistent with structural predictions above.

**Figure 6:**
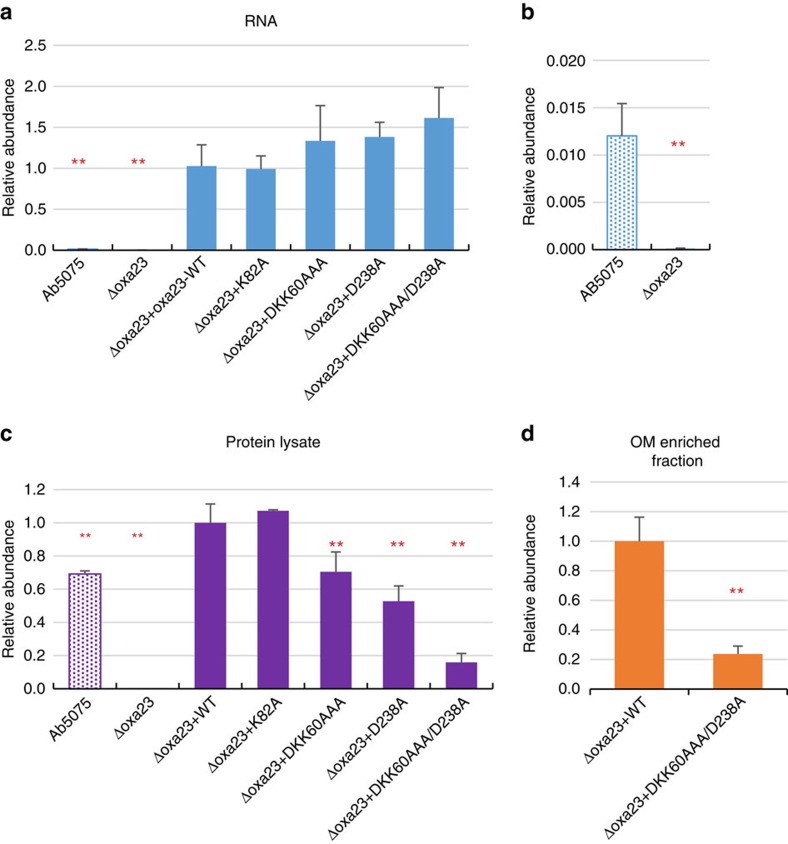
RNA- and protein-level comparison of *oxa-23* expression in *Δoxa23* complementation mutants. (**a**) Quantitative PCR measurements of *oxa-23* RNA level in AB5075, *Δoxa23* deletion mutant and *Δoxa23* complementation mutants. O*xa-23* RNA level was referenced to the *16S RNA*. Similar results were also obtained with an independent *oxa-23* primer set (Supplementary Fig. 11A). Error bars indicate the s.d. for the three biological replicates examined for each strain. **Statistical significance compared with *Δoxa23* complementation mutant with *WT-oxa23* gene (**P<0.01, one-way unpaired analysis of variance (ANOVA), *n*=3). (**b**) Zoom-in view of the relative *oxa-23* RNA level for AB5075 and *Δoxa23* deletion mutant. (**c**,**d**) Comparison of Oxa-23 protein levels in whole cell lysate (**c**) or OM protein-enriched fraction (**d**) in AB5075, *Δoxa23* deletion mutant and *Δoxa23* complementation mutants examined with parallel reaction monitoring (PRM) assays. Error bars indicate the s.d. for the three biological replicates. One microgram of protein digest was loaded for each PRM analysis. **Statistical significance compared to *Δoxa23* complementation mutant with *WT-oxa23* gene (**P<0.01, one-way unpaired ANOVA, *n*=3).

To further investigate the possible effects of the Oxa-23 mutations at 58DKK60 and D238 on carbapenem resistance in AB5075, we complemented the *Δoxa23* deletion strain with the *oxa-23* mutants DKK60AAA, D238A or DKK60AAA/D238A with the pMMB.A1 vector. Quantitative real-time PCR assays showed that all three mutants expressed equivalent RNA levels as the complemented *oxa-23* WT gene (Fig. 6a and Supplementary Fig. 11A). However, at the protein level, anti-Oxa23 immunoblots and parallel reaction monitoring assays detected consistent decreases for all three Oxa-23 charged-patch mutants, with DKK60AAA being at 70%, D238A at 50% and DKK60AAA/D238A at 10% of the complemented Oxa-23 WT protein level (Fig. 6c and Supplementary Fig. 11B). Interestingly, complementation with mutation at the catalytic site K82A did not show reduction in protein abundance, suggesting that the observed protein reduction was specific for the charged-patch mutations DKK60AAA and D238A. As mutants produced equivalent *oxa-23* RNA as the complemented *oxa-23* WT gene, the decreased protein abundance for the mutants resulted from posttranscriptional regulation for Oxa-23 proteins, meaning that either decreased translation rates or protein stability in DKK60AAA and D238A mutants caused lower protein levels. Based on the DNA sequence of the mutated residues, we confirmed that codon usage bias did not explain the observed decreased protein abundance. In addition, it is noteworthy that complementation strains were induced to produce 20-fold higher *oxa-23* RNA levels relative to AB5075 WT (Fig. 6b and Supplementary Fig. 11A); yet, at the protein level these difference were diminished to 1.5-fold or less (Fig. 6c). If regulated protein degradation of unbound Oxa-23 is a dominant source of protein homeostasis in the periplasm, the observed decrease in Oxa-23 protein levels resultant from charge-patch mutations intended to disrupt interactions with OM porins would appear consistent with this control mechanism.

We examined the carbapenem sensitivity changes of Oxa-23 site-directed complementation mutants with MIC assays. First, as expected, the catalytic site mutant K82A did not restore carbapenem resistance, despite protein levels equal to that of complemented WT Oxa-23, with meropenem MIC of 1 μg ml^−1^ and imipenem MIC of 0.5 μg ml^−1^ (Table 2 and Supplementary Figs 12 and 13). Second, mutants with charge-patch mutations, which cause defects in the proposed porin-localized antibiotic resistance mechanism, also exhibited increased carbapenem sensitivity (lower MIC values). For the single-patch complemented mutants (D238A and DKK60AAA), thinner lawn growth was observed at the 4 and 6 μg ml^−1^ imipenem concentrations and at the 6 μg ml^−1^ meropenem concentration compared with Oxa-23 WT complementation strain (Supplementary Figs 12 and 13), although the MIC values of the mutants were shown as small change or no change (Table 2). The ‘thinner lawn' phenotypes were repeatedly observed for all four DKK60AAA and all five D238A isolates tested with four independent MIC measurement experiments for imipenem and meropenem. These phenotypes are reproducible and are not random. For double-mutant DKK60AAA/D238A, twofold increase in meropenem sensitivity and fourfold increase in imipenem sensitivity were observed (Table 2 and Supplementary Figs 12 and 13), which represents a sizable fraction of the entire MIC reduction observed in *Δoxa23* deletion mutant. Although the meropenem susceptibility may be attributed to the decreased hydrolysis activity of DKK60AAA/D238A mutant, the imipenem sensitivity changes are independent of Oxa-23 enzymatic activity changes and therefore unequivocally indicate that the sites DKK60AAA/D238A influence the Oxa-23 function by mechanisms other than changes of hydrolysis activity (Supplementary Fig. 10). To ascertain whether DKK60AAA and D238A mutations affect membrane association of Oxa-23, we compared Oxa-23 protein levels purified in the OM-enriched fractions in WT versus *DKK60AAA/D238A* complementation strains. Figure 6d shows that the amount of DKK60AAA/D238A mutant detected in the OM-enriched fraction was ∼20% of the WT purified amount, consistent with the notion that Oxa-23 interactions with these OM porins are important for resistance.

## Discussion

With the evolving world-wide crisis of antibiotic resistance for microbial pathogens, new knowledge on antibiotic resistance mechanisms and pathogenesis is of critical importance. In the present study, XL-MS was used to provide the first view of the *in vivo* protein interaction network of the multidrug-resistant pathogenic bacterium, *A. baumannii* AB5075. With this unbiased approach, an extensive protein interaction network in AB5075 (2,068 cross-links for 245 intra-molecular and 398 inter-molecular interactions) was identified that includes many novel interactions among proteins from various cellular compartments. Oxa-23, a key carbapenemase in multidrug-resistant *A. baumannii* clinical isolates, OmpA, an important virulence factor in *A. baumannii* involved in host cell attachment, invasion and inhibition of host complement factors, and YiaD, an OmpA domain-containing OM protein were observed to be the most heavily linked protein interaction hubs in the network, comprising a total of 133 binary protein interactions and 495 inter-protein cross-links.

Most of the OXA-type carbapenemases show only weak *in vitro* carbapenemase activity, leading to previous speculation that *in vivo* carbapenem resistance may result from a combined action of an OXA-type carbapenemase and a secondary resistance mechanism such as porin deficiencies or overexpressed efflux pumps. However, *in vivo* cross-linking data illustrate that in cells, Oxa-23 proteins interact with several membrane porin proteins at their periplasmic surfaces. This finding reveals increased complexity of the carbapenem resistance mechanisms in AB5075. Neither transposon mutants with inactivation of *carO* (an imipenem channel candidate^55^) or *ompA* (a major porin in *A. baumannii*^15^) resulted in increased resistance of AB5075 to carbapenems. This suggests that either CarO or OmpA porins did not possess abundant channel activity for carbapenems *in vivo*^60^, redundancy exists in channels that uptake carbapenems, or that the losses of CarO and OmpA proteins also triggered protein interaction network changes of Oxa-23 that counterbalance the effects of CarO/OmpA porin losses. Furthermore, interactions of Oxa-23 and porins could conceivably benefit the function of porins as well. Given that membrane porins serve as channels for both the nutrient uptake and antibiotic penetration into bacterial cells^55^,^61^, the binding of Oxa-23 at the exit of porin channels can serve to act as a filter with enzymatic specificity to eliminate toxic antibiotics, while maintaining porin function for useful nutrient uptake^62^. We refer to this model of interaction as ‘porin-localized toxin inactivation' (Fig. 3a). Notably, we also identified interactions of superoxide dismutase (SodC) with the periplasmic faces of porins OmpW and CarO (Supplementary Fig. 14), which suggests that porin localization of protective enzymes may be a general advantageous strategy employed in these bacteria.

Abundant immunoblot signals were observed for Oxa-23 in OM protein-enriched fractions. This finding suggests that a significant population of Oxa-23 proteins may be associated with OM porins *in vivo*, consistent with the cross-linking identification of Oxa-23 and OM porins. The topology of Oxa23-porin complexes derived from cross-linked lysine pairs (Fig. 3b,c) reveals that the catalytic site of Oxa-23 K82 is thereby concentrated nearby the inner surface of the OM. This orientation enables increased Oxa-23 catalytic activity near OM porins, which would benefit hydrolysis of antibiotics immediately entering cells, before their diffusion into the cellular volume. Moreover, even if the OM porins that associate with Oxa-23 are not the primary channels transporting carbapenems, interactions identified in this work serve to localize and concentrate Oxa-23 near the inner surface of the OM, a region through which carbapenems must diffuse before exerting their mechanism of action. These results may provide new insight on the paradoxical relationship between the strong co-segregation of Oxa-23 in carbapenem-resistant clinical isolates and the low efficiency of Oxa-23 carbapenemase activity observed from *in vitro* kinetic analysis^43^,^63^.

*Δoxa-23* complementation mutants with mutations of interaction interfaces DKK60AAA, D238A and DKK60AAA/D238A were observed with decreased Oxa-23 protein levels compared with complementation strain with WT *oxa-23*. The DKK60AAA/D238A mutant also showed an increase in carbapenem sensitivity. These results verified the functional relevance of the Oxa-23 58DKK60 and D238 residues, and the identified Oxa23-porin cross-links. The results in this work suggest that the membrane association of Oxa-23 benefits Oxa-23 protein stability by either directly protecting Oxa-23 from proteolysis, or by reducing the protein aggregation (and subsequent degradation) of Oxa-23 in periplasm^64^, and in consequence, enables higher protein concentration of Oxa-23 in periplasm^63^ and increases carbapenem resistance. Sequence alignment with ‘OXA-' genes in *Acinetobacter* species in Uniprot (4,933 entries) confirmed that the charged patches 58DKK60, D238 are highly specific for Oxa-23 protein and the Oxa-23-like protein family^10^, indicating that not only the carbapenemase activity but also the evolved charged patches are distinct features for Oxa-23 enzyme to contribute to carbapenem resistance in *A. baumannii* isolates. New knowledge on Oxa-23 membrane association represents new opportunities to manipulate Oxa-23 function *in vivo* and enhance the clinical efficacy of antibiotic treatments. Further work is needed to test whether porin-localized antibiotic inactivation occurs in other *A. baumannii* isolates or in other bacteria.

## Methods

### *A. baumannii* AB5075 WT and mutant strains

*A. baumannii* strain AB5075 was provided by the laboratory of D. Zurawski at Walter Reed Army Institute of Research and was described previously^7^,^8^. AB5075 transposon insertion mutants for *oxa-23*, *ompA*, *carO*, *ampC*, *oxa-69*, *ompW*, *yiaD*, *ABUW_2898* and *ABUW_0724* were obtained from the sequence-verified transposon mutant library for AB5075 (ref. 54).

The Δ*oxa23* deletion mutant was constructed by allelic exchange with a PCR product, which was obtained with Gene Splicing by Overlap Extension method (SOEing PCR). The SOEing PCR product joined sequences of 600 bp 5′-upstream of *oxa-23* allele, the 5′ first 31 bp of *oxa-23* gene, a 6 bp BamHI digestion site, the 3′ last 34 bp of *oxa-23* gene and the 600 bp 3′ downstream of *oxa-23* allele. A stop codon is encoded at the 42–44 bp position of the allelic exchanged site. The joined sequence was cloned into a pSL15A plasmid, which contains a Tet-SacB counter-selection system, using XbaI and SacI digestion sites. Constructs were transformed to *E. coli* NEB-5α cells (New England Biolabs, Ipswich, MA, USA) and positive colonies were selected with tetracycline (Tc) 10 μg ml^−1^ and were verified by colony PCR. The positive constructs were purified from NEB-5α cells using QIAprep Spin Miniprep Kit and transformed to *E. coli* S17 mating strain^65^ selected with Tc 10 μg ml^−1^. To transform pSL15A*-*Δ*oxa23* constructs into AB5075, S17 mating cells and AB5075 recipient cells were mixed in a 1:1 ratio (based on OD_600_), and were incubated in Luria-Bertani (LB) agar for 6 h at 37 °C. Cells were scraped off the plate with PBS and were plated on LB agar plates containing Tc 15 μg ml^−1^ and chloramphenicol (Cm) 5 μg ml^−1^. Positive colonies were inoculated in LB medium and were streaked on LB agar plates (containing 6% sucrose). The positive colonies that were resistant to sucrose and sensitive to Tc 15 μg and Cm 5 μg ml^−1^ (that is, *Δoxa23* deletion mutant candidates) were selected. The deletion of the *oxa-23* allele in *Δoxa23* mutants were confirmed with colony PCR.

*Δoxa23* complementation mutants were constructed with pMMB.A1 vector. The pMMB.A1 is a derivative of pMMB-HA-Strep^66^ vector and contains a partial lacIq gene deletion that enabled constitutive expressions of the inserted gene. The XbaI and KpnI cloning sites were used for the insertion of *oxa-23* gene (including signal peptide sequence) and a TAA stop codon was included at the end of *oxa-23* gene, to avoid protein expressions of C-terminal tags. Complementation mutants were selected with LB agar plates containing Tc 15 μg ml^−1^ and Cm 5 μg ml^−1^.

Site-directed mutants of *oxa-23* were obtained with QuikChange II Site-Directed Mutagenesis Kit (Agilent Technologies, Santa Clara, CA, USA) following instruction manual, using the template of WT *oxa-23* in pMMB.A1 vector or in pET-28b vector (EMD Millipore, Billerica, MA, USA). The mutated residues were verified by DNA sequencing.

AB5075-pMMB-*oxa23*-HA-Strep strain was obtained by transformation of pMMB-*oxa23*-HA-Strep DNA into AB5075 WT cells. The TAA stop codon was omitted at the end of *oxa-23* gene to obtain the HA-Strep tag version of Oxa-23 proteins. Mutant cells were selected with LB agar plates containing Tc 15 μg ml^−1^ and Cm 5 μg ml^−1^.

A summary of the plasmids and strains used in the study is provided in Supplementary Table 1.

### Sequence-specific antibodies

Polyclonal sequence-specific antibodies for OmpA (against peptide 134 CIPDLSYHNDEEGTL 147)^67^, CarO (for peptide 127 CNDYDLTRNVDATRS 140) and Oxa-23 (for peptide 55 IQTDKKINLYGNALC 68) were obtained from Genscript (Piscataway, NJ, USA). Polyclonal anti-HA antibodies were obtained from Sigma-Aldrich (St Louis, MO, USA).

Information related to co-IP and immunoblot analyses is provided in Supplementary Methods. Full blot images for Fig. 4 and Supplementary Figs 7 and 11 are provided in Supplementary Figs 15–17.

### Synthesis of chemical cross-linkers

The PIR cross-linker BDP-NHP was synthesized as previously described^22^,^28^ and confirmed by direct infusion electrospray ionization–MS analysis. Additional synthesis details are provided in Supplementary Methods.

### *A. baumannii* cell cultures and cross-linking reactions

*A. baumannii* AB5075 cells were grown with LB media to late log phase (OD_600_=1.0). Cells were pelleted and washed with PBS three times to remove free amines, which may interfere with cross-linking reactions. Cells were cross-linked in the buffer of 500 mM Na_2_HPO_4_, pH 7.4 and 150 mM NaCl, with three doses of BDP-NHP cross-linkers with an incubation time of 3H and with shaking at 1,300 r.p.m. with a thermomixer at room temperature. This cross-linking condition was confirmed not to lyse the AB5075 cells (Supplementary Fig. 1). Reactions were quenched by adding 20 mM Tris-HCl pH 8.0, to reaction mixtures. Cell pellets were collected with centrifugation at 3,000 *g* at 4 °C for 15 min for proteomics analysis. Three biological replicates were analysed, which means that cells were grown up three times and independently cross-linked and analysed with liquid chromatography (LC)–MS analysis.

### Proteomics sample preparations

*A. baumannii* proteins were extracted by dissolving the cell pellets with lysis buffer (100 mM Tris, pH 8.0, 4% SDS and 10 mM dithiothreitol), assisted with 95 °C heating and sonication of extraction mixtures. SDS was removed through buffer exchange with Amicon (10 kDa molecular weight cut-off (MWCO)) (Millipore, Billerica, MA, USA) to the urea buffer (50 mM NH_4_HCO_3_ and 8 M Urea). Protein concentrations were measured with Bradford assays. Proteins were further alkylated with iodoacetamide and digested with trypsin (Promega, Madison, WA, USA). Tryptic peptides were purified with C18 Sep-Pak cartridges (Waters, Milford, MA, USA) before strong cation exchange (SCX) analysis.

SCX peptide fractionation was performed with an Agilent 1200 system (Agilent Technologies) using a 5 cm BioBasic SCX guard column (5 μm, 300 Å; Thermo Fisher Scientific, Waltham, MA, USA) and a PolySULFOETHYL Aspartamide SCX column (4.6 mm × 100 mm, 12 μm, 300 Å; Nest Group Inc., Southborough, MA). The buffer system of 25% acetonitrile, 0.5% formic acid and ammonium acetate was used. A step gradient of ammonium acetate concentrations (0, 25, 50, 75, 100, 150, 200, 300, 400 and 500 mM) was used to elute peptides at a flow rate of 1.5 ml min^−1^, with each fraction collected for 5 min.

The SCX elutes were dried in an EZ2-Plus evaporator (Genevac, Gardiner, NY, USA), and resuspended with biotin capture buffer (100 mM Tris; pH 8.0, 150 mM NaCl and 0.1% NP-40) and adjusted pH to be ∼7.5 by addition of 1 M Tris pH 8.0. The cross-linked peptides were purified with Monomeric Avidin UltraLink resin (Thermo Fisher Scientific) and beads were washed five times with buffer ([100 mM Tris pH 8.0 and 150 mM NaCl) and one time with H_2_O, before eluting with buffer (70% acetonitrile and 0.5% formic acids). Elutes were dried and resuspended with 0.1% formic acid for LC–MS analysis.

In addition to the in-solution digestion approach, *A. baumannii* protein extracts of cross-linked cells were also analysed by SDS–PAGE. Gels of high-molecular-weight regions (>100 kDa and stacking gels) were excised for cross-linked peptide identification (Supplementary Fig. 2). In-gel digestion was performed at 37 °C overnight in the buffer of (40 mM NH_4_HCO_3_ and 5% acetonitrile pH 8.0) and with trypsin concentration of 12.5 ng μl^−1^. Peptides were extracted from gels with the buffer of (50% acetonitrile and 0.1% trifluoroacetic acid) and speedvac dry. Peptides were resuspended with biotin capture buffer and cross-linked peptides were enriched with Monomeric Avidin UltraLink resins as described above for LC–MS analysis.

### LC and MS analysis

The purified cross-linked peptides were separated with C8 reversed-phase chromatography using a 3 cm trap column (5 μm, 200 Å; Bruker, Billerica, MA, USA) and a 60 cm analytical column (5 μm, 100 Å) in a NanoAcquity UPLC system (Waters). A column heater set to 45 °C was used to help control column pressure and homogeneity of peptide separation. The linear separation gradient of [10% A, 90% B] to [60% A, 40% B] at a flow rate of 300 nl min^−1^ was used, in which solvent A was H_2_O containing 0.1% formic acid and solvent B was acetonitrile containing 0.1% formic acid. The gradient time was 240 min for SCX fractionated samples and 60 min for in-gel purified samples. Peptides were electrosprayed into a Velos-FTICR mass spectrometer^68^ (Thermo Fisher Scientific).

The Velos-FTICR was operated in the ReACT mode^26^. Briefly, precursor ions with charge states of 4+ or higher were identified in FT-ICR (Fourier transform ion cyclotron resonance) MS^1^ scans and were selected for MS^2^ fragmentation analysis. The MS^2^ scan was analysed with FT-ICR and, if the accurate masses of the MS^2^ fragment masses were able to construct a mass relationship with the MS^1^ precursor mass within 20 p.p.m. mass errors (allowing two ^13^C isotope offsets), the matched MS^2^ fragments were targeted for MS^3^ fragmentation analysis. MS^3^ scans were performed with the low-resolution ion trap instrument. Additional information of the ReACT analysis is provided in Supplementary Fig. 2 and Supplementary Methods.

### MS data analysis

The raw data of MS^2^ precursor mass and MS^3^ fragmentation spectra were converted to mzXML files with ReAdW (version 4.2.1). The mzXML data were searched against an experimentally developed stage 1 protein database^69^ for *A. baumannii* containing forward and reverse protein sequences (total of 3,482 entries), using Comet (version 2015.01 rev.02)^70^. The 1,741 proteins included in the stage 1 database were identified as putative BDP-NHP reactive proteins (Supplementary Methods and Supplementary Data 3). Search settings included precursor mass tolerance 20 p.p.m., ^13^C isotope offsets (−1/0/1/2/3) enabled, peptide termini fully tryptic, maximum internal cleavage 3, fragment ion tolerance 1.0005 Da (with 0.4 Da fragment offset), fixed modification cysteine carbamidomethylation, variable modifications lysine stump (197.032422 Da), protein N termini stump (197.032422 Da) and methionine oxidation. The FDR for peptide identification (FDR) was determined by the ratio of the number of reverse hits to the number of target hits. Peptide identification threshold (FDR<5%) was used to obtain peptide candidates that were included to assign cross-linked peptide pairs. Furthermore, peptide candidates that were mapped to cross-link pairs were required to (1) have an internal lysine with stump modification; (2) C-terminal-cleaved lysine residues cannot be a stump modified lysine; (3) the peptide can be paired to another peptide that is also identified<5% FDR in the same ReACT cycle; and (4) in cases that two MS^3^ events for the same precursors yielded different peptide IDs, the ID with the better expectation value was selected.

Using this procedure, a total of 22,647 cross-linked peptide pairs could be assigned. In addition, decoy peptides that passed FDR<5% threshold were also mapped to PIR relationship, to construct decoy cross-link pairs (containing one or both peptide arms with decoy peptides), and 440 decoy pairs were observed. The FDR for the cross-linked peptide pairs (FDR_link_) was estimated with the ratio of the number of decoy pairs to the number of target pairs, which was 440/22,647=1.9%.

### Antibiotic sensitivity assays

The MIC assays were performed as previously described^71^. *A. baumannii* cells were grown in LB medium at 37 °C overnight. The cell density of each strain was normalized and diluted to OD_600_=1.0 with LB medium. The normalized cell suspension was further diluted 1,000-fold with LB medium and 5 μl of the diluted cell cultures (∼1 × 10^4^ cells) were spotted onto the LB agar plates that contained meropenem or imipenem antibiotics (Sigma-Aldrich). Concentration series of meropenem and imipenem included 0, 0.25, 0.5, 1, 2, 3, 4, 6, 8, 12 and 16 μg ml^−1^. Plates were incubated at 37 °C for 24 h and photographed. MIC is defined as the lowest antibiotic concentration preventing the lawn growth of the spotted cells.

For the agar diffusion assays, overnight *A. baumannii* cell cultures were diluted to OD_600_=0.2. Cells were spread onto a LB agar plates with a sterile cotton swap with a plastic shaft. The antibiotic discs (BBL Sensi-Disc, Becton, Dickinson and Company, Franklin Lakes, NJ, USA) were placed onto the bacterial lawn. Cell growth was allowed for 24 h at 37 °C. The annular radius (mm) of the inhibition zone was measured.

### Data availability

The MS cross-linking data have been deposited in the ProteomeXchange Consortium via the PRIDE partner repository^72^ with the data set identifier PXD004236. The authors declare that all other data supporting the findings of this study are available within the article and its Supplementary Information files or from the corresponding author upon request.

## Additional information

**How to cite this article:** Wu, X. *et al.* *In vivo* protein interaction network analysis reveals porin-localized antibiotic inactivation in *Acinetobacter baumannii* strain AB5075. *Nat. Commun.* **7,** 13414 doi: 10.1038/ncomms13414 (2016).

**Publisher's note:** Springer Nature remains neutral with regard to jurisdictional claims in published maps and institutional affiliations.

## Supplementary Material

Supplementary Information Supplementary Figures 1-17, Supplementary Table 1, Supplementary Methods and Supplementary References

Supplementary Data 1 Cross-linked peptide pairs and protein-protein interactions identified in this study.

Supplementary Data 2 Protein domain information relevant to Supplementary Fig. 3.

Supplementary Data 3 Proteins included in the stage 1 database. These are protein candidates that were labelled with BDP-NHP cross-linkers in AB5075 cells and identified with DDA analysis (Supplementary Methods).

## Figures and Tables

**Table 1:** MIC for AB5075 transposon mutants.

**Strains**	**Oxa-23 binders?**	**OmpA binders?**	**YiaD binders?**	**CarO binders?**	**Meropenem MIC (μg ml**^**−1**^**)**	**Imipenem MIC (μg ml**^**−1**^**)**
AB5075 WT					8	8
*oxa-23 Tn* (105/822)	—	Y	Y	Y	2–4	1
*carO Tn* (282/741)	Y	Y	Y	—	4–8	4
*ompA Tn* (264/1062)	Y	—	Y	Y	4–8	4
*ampC Tn* (736/1152)					8	8
*ampC Tn* (420/1152)					8	8
*oxa-69 Tn* (356/825)					8	8
*oxa-69 Tn* (192/825)					16	8
*ompW Tn* (328/582)	Y	Y	Y		16	8
*ompW Tn* (147/582)	Y	Y	Y		16	8
*yiaD Tn* (120/654)	Y	Y	—	Y	16	4–8
*yiaD Tn* (202/654)	Y	Y	—	Y	16	4–8
*ABUW_2898 Tn* (678/1446)	Y	Y			8–16	8
*ABUW_2898 Tn* (781/1446)	Y	Y			8–16	8
*ABUW_0724 Tn* (477/1431)				Y	16	8
*ABUW_0724 Tn* (225/1431)				Y	16	8
AB19606 WT					1	0.5

MIC, minimal inhibitory concentration; WT, wild type.

AB5075 transposon mutants were obtained from the AB5075 transposon mutant library^54^. Numbers in parentheses indicate the transposon insertion site and the full length of the gene sequence. The MIC (μg ml^−1^) is the lowest antibiotic concentration preventing the lawn growth of the bacteria. Range values indicate variations of MIC values for individual isolates of each strain. Four individual isolates were examined for each strain. Protein interaction information with Oxa-23, OmpA, YiaD or CarO is indicated with ‘Y'.

**Table 2:** MICs for *Δoxa23* complementation mutants.

**Strains**	**Meropenem MIC (μg ml**^**−1**^**)**	**Imipenem MIC (μg ml**^**−1**^**)**
AB5075 WT	8	8
*Δoxa23*	1	0.5
*Δoxa23+pMMB.A1-oxa23-WT*	8	8
*Δoxa23+pMMB.A1-oxa23-K82A*	1	0.5
*Δoxa23+pMMB.A1-oxa23-DKK60AAA*	8	6–8
*Δoxa23+pMMB.A1-oxa23-D238A*	6–8	6
*Δoxa23+pMMB.A1-oxa23-DKK60AAA/D238A*	3–4	2

MIC, minimal inhibitory concentration; WT, wild type.

*Δoxa23* deletion mutant was complemented with WT *oxa-23* gene or *oxa-23* site-directed mutants using pMMB.A1 vector. The MIC (μg ml^−1^) is the lowest antibiotic concentration preventing the lawn growth of the bacteria. Range values indicate variations of MIC values detected with individual isolates of each strain. Four individual isolates were examined for each strain. See Supplementary Figs 12 and 13 for more details.
