# Supplementary material for: In vivo protein interaction network analysis reveals porin-localized antibiotic inactivation in Acinetobacter baumannii strain AB5075
Source: Nat Commun. 2016 Nov 11;7:13414. doi: 10.1038/ncomms13414 (PMC5114622; doi:10.1038/ncomms13414)
Supplement: Supplementary Information — Supplementary Figures 1-17, Supplementary Table 1, Supplementary Methods and Supplementary References [file ncomms13414-s1.pdf]

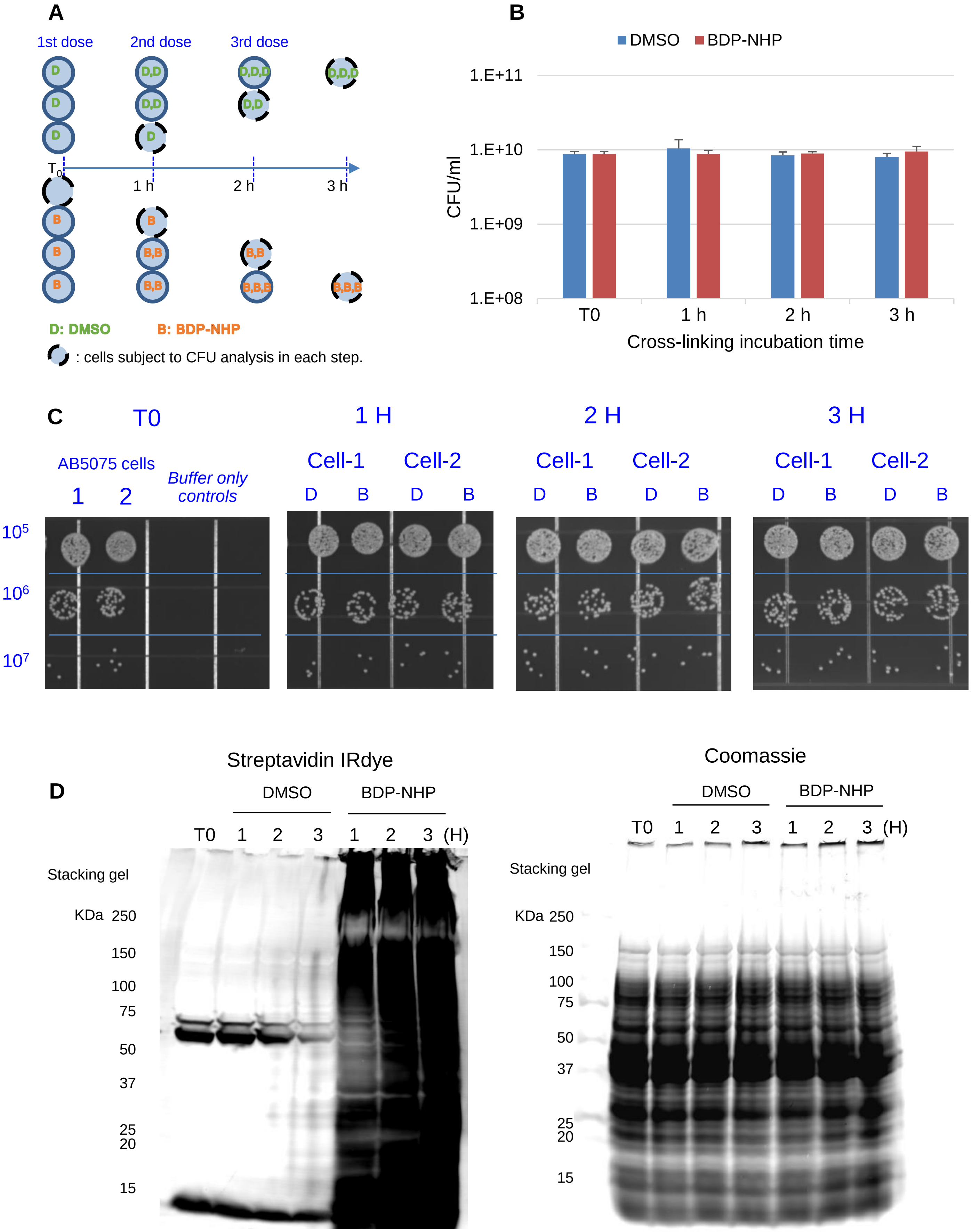
A
1st dose
2nd dose
3rd dose
D
D,D
D,D,D
B
B,B
B,B,B
T0
1 h
2 h
3 h
D: DMSO
B: BDP-NHP
: cells subject to CFU analysis in each step.
B
DMSO
BDP-NHP
CFU/ml
1.E+11
1.E+10
1.E+09
1.E+08
T0
1 h
2 h
3 h
Cross-linking incubation time
C
T0
1 H
2 H
3 H
AB5075 cells
1
2
Buffer only controls
Cell-1
Cell-2
D
B
10^5
10^6
10^7
D
Streptavidin IRdye
Coomassie
DMSO
BDP-NHP
T0
1
2
3
(H)
Stacking gel
KDa
250
150
100
75
50
37
25
20
15

Supplementary Fig. 1 Validation of *in vivo* protein cross-linking with AB5075 cells. A) Experimental design. AB5075 cells of OD_{600} = 1.0 grown in LB medium were harvested, washed three times with PBS, and aliquoted to seven tubes in buffer 500 mM Na_2HPO_4 (pH 7.4), 150 mM NaCl in 0.1 ml reaction volume. Colony forming unit (CFU) analysis was performed with cells at time points of T_0 (before addition of BDP-NHP cross-linkers), 1 hour (after cross-linking with 5 mM BDP-NHP), 2 hours (after cross-linking with two doses of 5 mM BDP-NHP), and 3 hours (after cross-linking with three doses of 5 mM BDP-NHP). Equivalent treatments with the solvent DMSO were also analyzed. B) CFU analysis showing that there were no detectable live cell loss of AB5075 after up to three hours cross-linking treatments with BDP-NHP. Error bars indicate the standard deviation of biological replicates (n=4). C) AB5075 cells before/after cross-linking were spotted onto LB agar plate to illustrate that AB5075 cells were live after the BDP-NHP cross-linking treatments. D) Infrared dye-labeled Streptavidins were used to detect the biotinylated proteins. Strong signals were observed for samples exposed to BDP-NHP cross-linkers. The reactive bands in T_0 and DMSO control samples may be resultant from the endogenous biotin containing proteins in AB5075. Coomassie stain examining the protein mobility shift in SDS-PAGE due to protein cross-linking. Results show that although cross-linking reactions indeed occurred to AB5075 cells, the protein fractions that were labeled in each cell may still be small, and therefore cross-linking did not result in significant loss of viable cells.

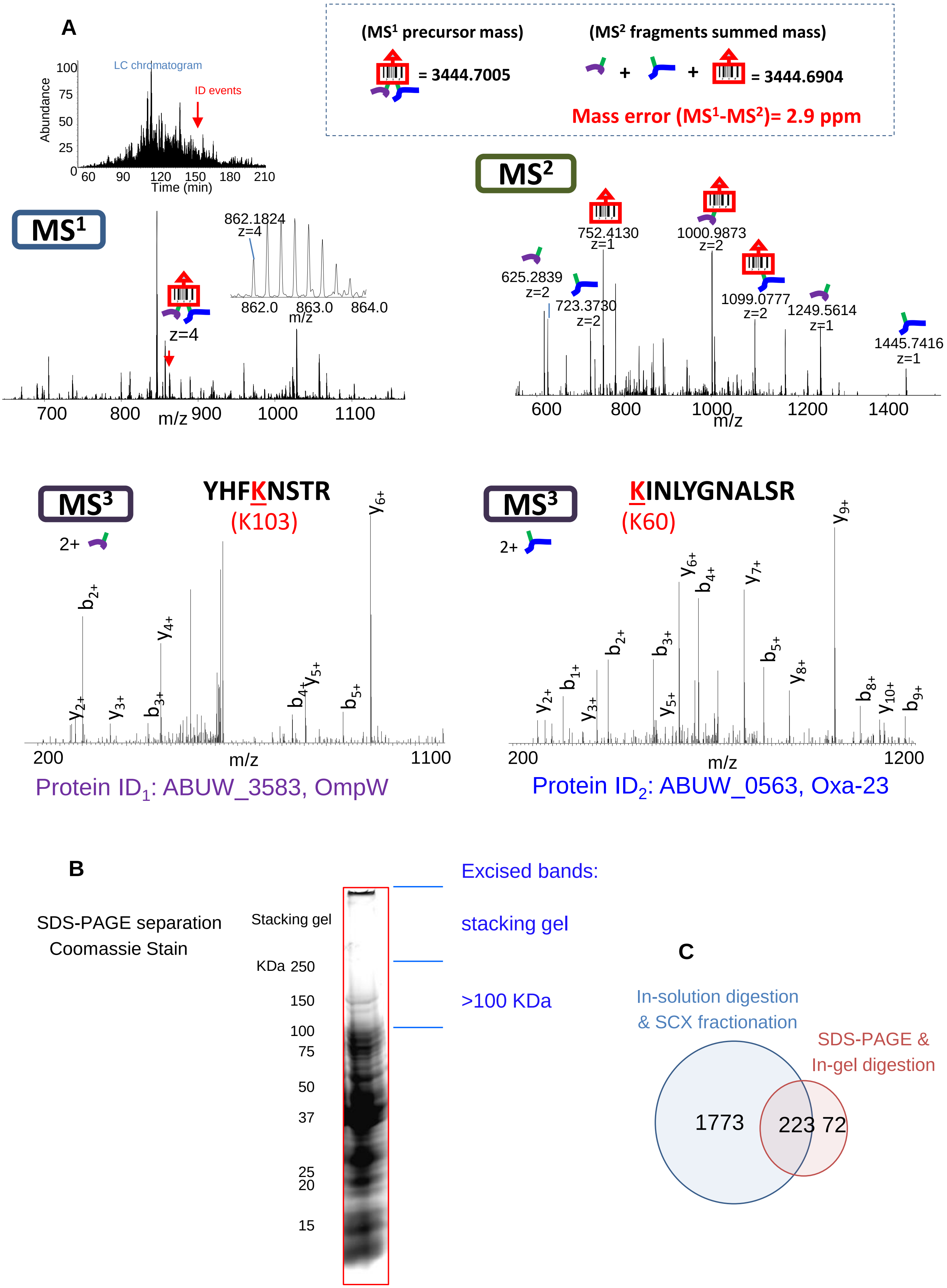
A
Abundance
100
75
50
25
0
LC chromatogram
ID events
60 90 120 150 180 210
Time (min)
(MS¹ precursor mass)
= 3444.7005
(MS² fragments summed mass)
+ +
= 3444.6904
Mass error (MS¹-MS²)= 2.9 ppm
MS¹
862.1824
z=4
862.0 863.0 864.0
m/z
z=4
700 800 m/z 900 1000 1100
MS²
752.4130
z=1
1000.9873
z=2
625.2839
z=2
723.3730
z=2
1099.0777
z=2
1249.5614
z=1
1445.7416
z=1
600 800 1000 1200 1400
m/z
MS³
2+
YHFKNSTR
(K103)
y6+
b2+
y4+
y2+
y3+
b3+
b4+
y5+
b5+
200 m/z 1100
Protein ID1: ABUW_3583, OmpW
MS³
2+
KINLYGNALSR
(K60)
y9+
y6+
b4+
y7+
b2+
b3+
b5+
y8+
b1+
y2+
y3+
y5+
b8+
y10+
b9+
200 m/z 1200
Protein ID2: ABUW_0563, Oxa-23
B
SDS-PAGE separation
Coomassie Stain
Stacking gel
KDa 250
150
100
75
50
37
25
20
15
Excised bands:
stacking gel
>100 KDa
C
In-solution digestion
& SCX fractionation
SDS-PAGE &
In-gel digestion
1773
223
72

Supplementary Fig. 2 Cross-linked peptide pair identification with Protein Interaction Reporter (PIR) technology. A) Real-time Analysis for Cross-linked peptide Technology (ReACT). The method consists of three steps. In the first step (MS^1), MS mass spectra are collected, and ions of charge states ≥ 4+ are identified and selected for the low energy MS/MS fragmentation analysis. In the second step (MS^2), MS/MS mass spectra are collected, peptide fragments that matched the mass relationship to MS^1 precursors are identified. In the third step (MS^3), the matched fragments are targeted for the MS/MS/MS analysis to obtain peptide fragmentation spectra that yield the peptide sequence information. Example showing the identification of the cross-linked peptide pair for β-lactamase Oxa-23 and outer membrane porin OmpW. The stump modified lysines are indicated, which were the cross-linked lysine residues. B) As an alternative approach of sample fractionation, proteins extracted from the BDP-NHP cross-linked cells were separated with SDS-PAGE. Gel regions corresponding to stacking gels and proteins bands > 100 KDa were excised for in-gel digestion analysis. C) Comparison of the cross-linked peptide pairs identified with SCX fractionation or SDS-PAGE separation approaches. The SCX approach contributed to a bigger number of the identified cross-linked peptide pairs. However, the SDS-PAGE analysis enabled identification of 72 cross-linked peptide pairs (24.4% of the 295 identification) that were not included in the SCX dataset.

AB5075 domain interaction network

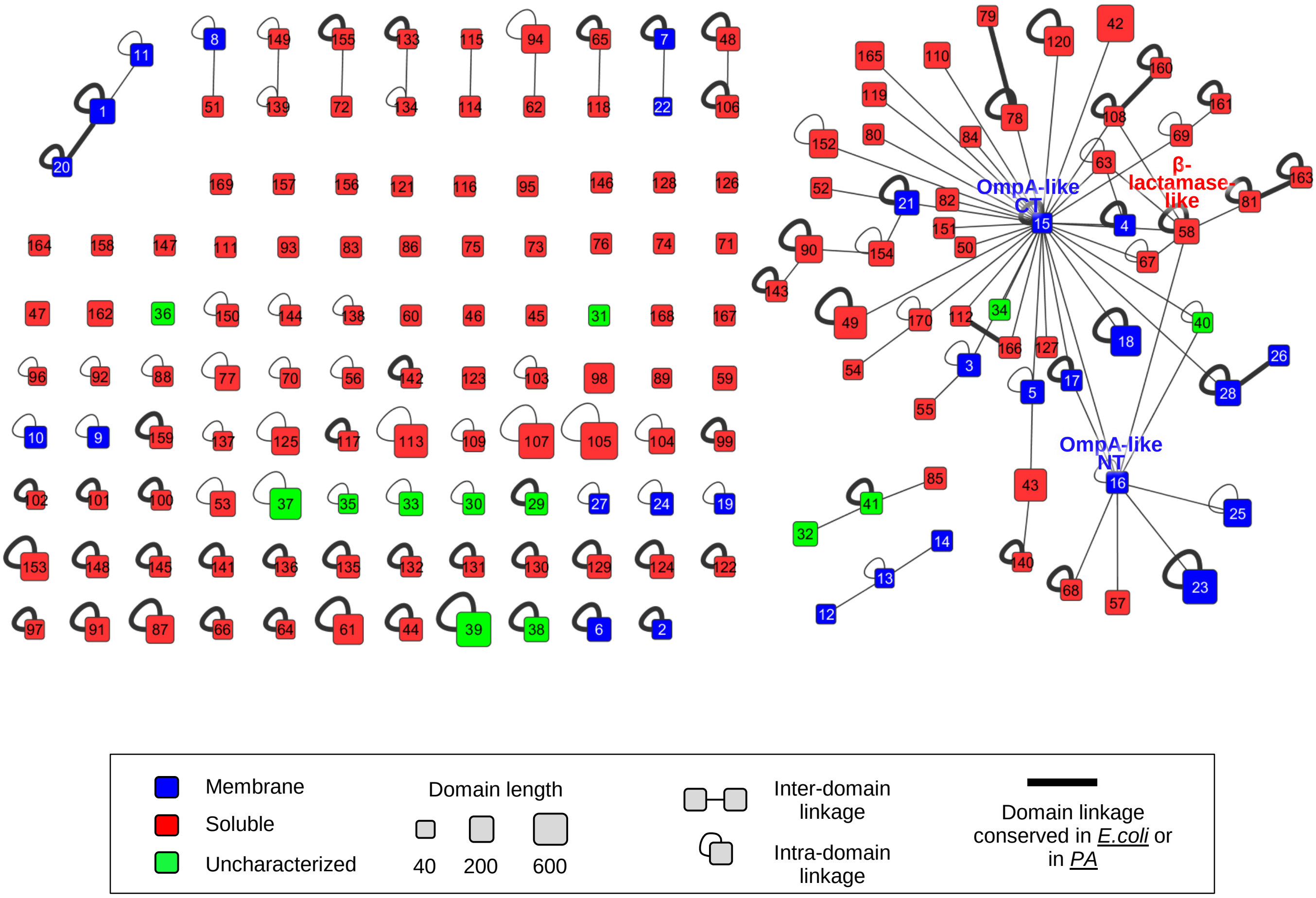

Supplementary Fig. 3 Domain interaction network in AB5075. Cross-linked proteins were mapped to protein domains based on SUPERFAMILY 1.75 (E value ≤ 1.0E-4). Interaction network was generated with Cytoscape 3.0.2. Node colors indicate the primary subcellular localization of proteins mapped to the domains. Blue: membrane proteins, Red: soluble proteins, and Green: uncharacterized proteins. Node sizes are proportion to the length of domains. Domain interaction hubs are indicated, including 15, OmpA-like CT domain; 16, OmpA-like NT domain; and 58, β-lactamase/transpeptidase-like domain. The complete information of the domains (node number) is provided in Supplementary Data 2. Singleton nodes are domains only containing intra-domain linkages, or those interacting with proteins of unknown domains. Thick edges showing conserved domain interactions identified in *E. coli*[1] or in *P. aeruginosa* (*PA*)[2].

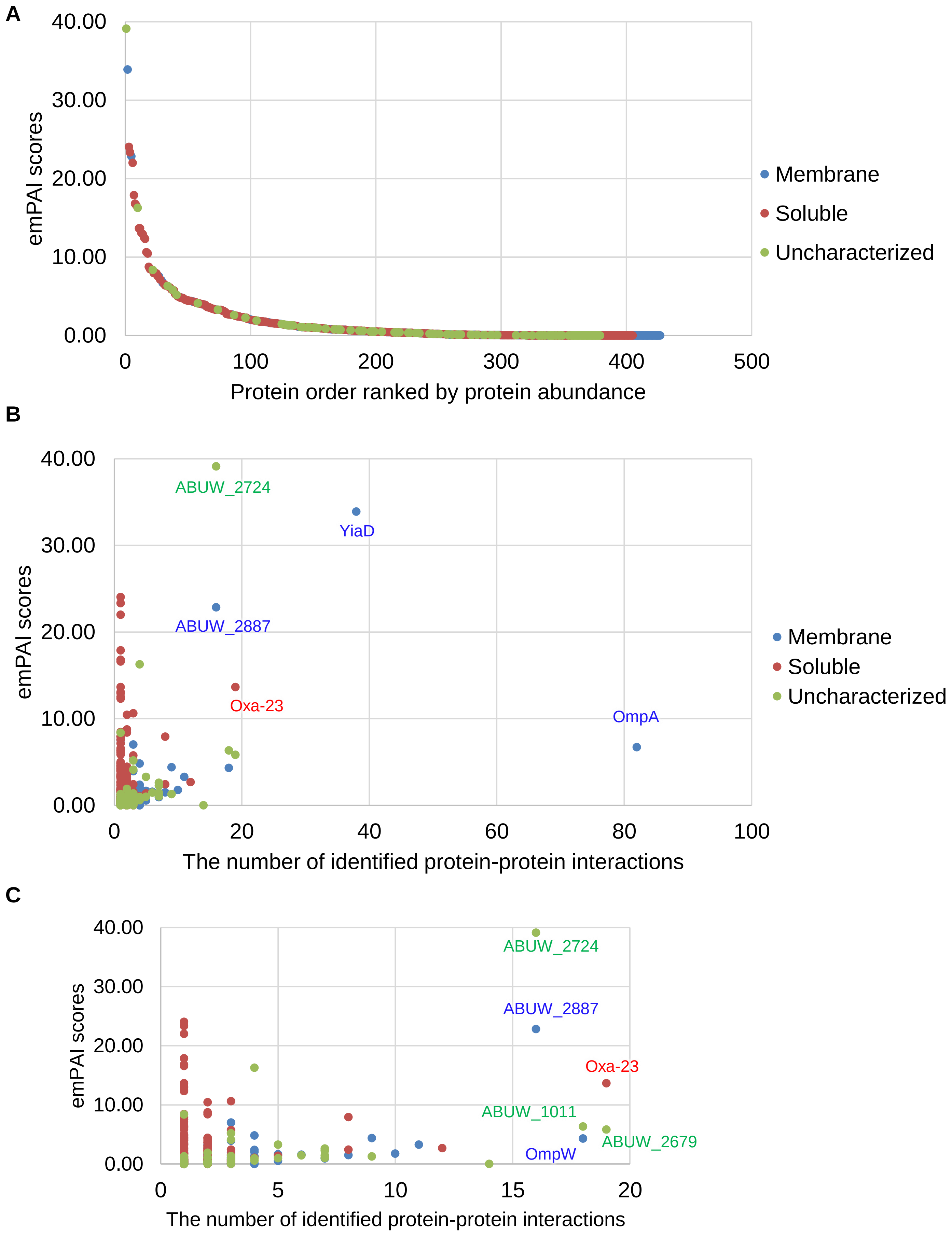

A
40.00
30.00
20.00
10.00
0.00
emPAI scores
0
100
200
300
400
500
Protein order ranked by protein abundance
Membrane
Soluble
Uncharacterized
B
40.00
30.00
20.00
10.00
0.00
emPAI scores
ABUW_2724
YiaD
ABUW_2887
Oxa-23
OmpA
0
20
40
60
80
100
The number of identified protein-protein interactions
Membrane
Soluble
Uncharacterized
C
40.00
30.00
20.00
10.00
0.00
emPAI scores
ABUW_2724
ABUW_2887
Oxa-23
ABUW_1011
ABUW_2679
OmpW
0
5
10
15
20
The number of identified protein-protein interactions

Supplementary Fig. 4 Comparison of the relative protein abundance for the identified cross-linked proteins. LC-MS/MS DDA analysis was performed for the non-crosslinked AB5075 cell lysates. Relative protein abundance was estimated with the Exponentially Modified Protein Abundance Index (emPAI)[3]. Membrane proteins (blue,) soluble proteins (red) and uncharacterized proteins (green) were categorized as Fig. 1D. A) Over three orders of magnitude were observed for the emPAI derived protein abundance for the cross-linked proteins in AB5075. B) No strong correlation was observed between the emPAI derived protein abundance and the number of identified protein-protein interactions, suggesting the specificity of *in vivo* PIR cross-linking. C) Inset showing X-axis from 0-20 range. Proteins identified with the large number of protein interactions, including OmpA, YiaD, Oxa-23, ABUW_2887, OmpW, ABUW_2724, ABUW_1011 and ABUW_2679 are highlighted.

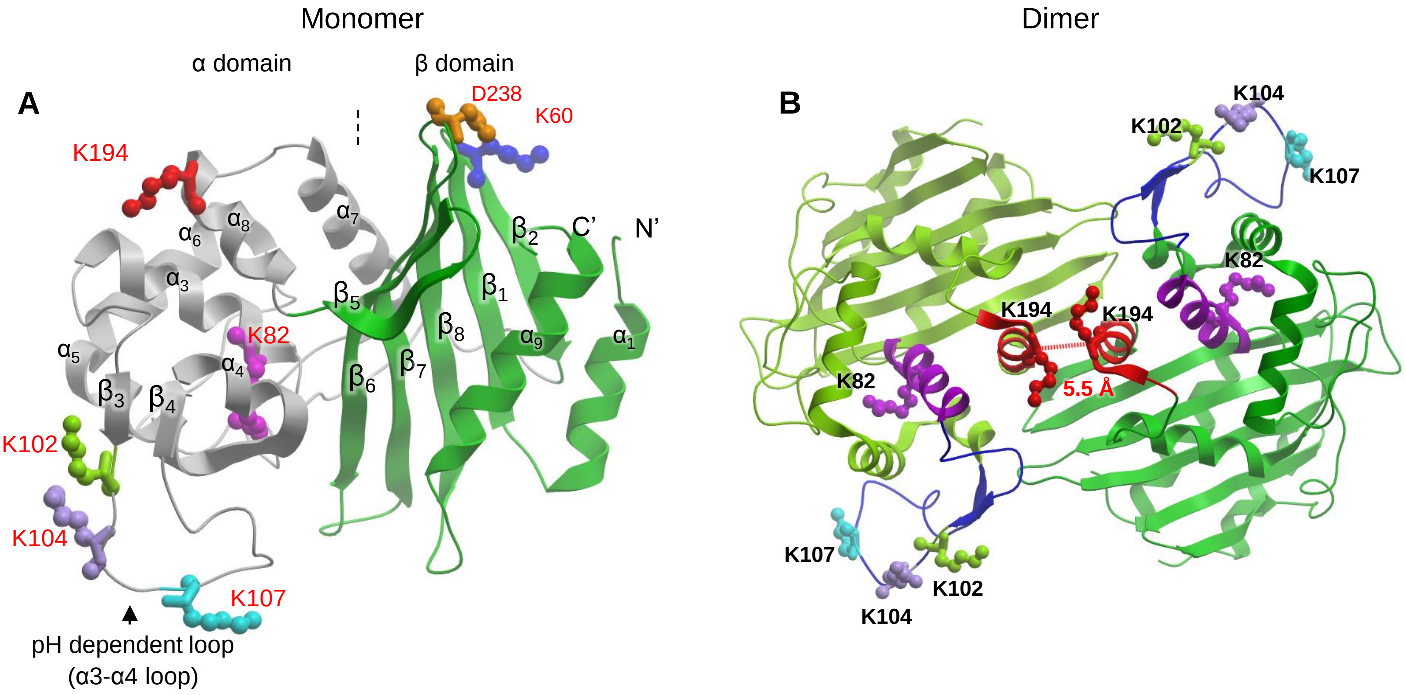

Supplementary Fig. 5 Oxa-23 crystal structure and homooligomeric interactions in AB5075. A) 3D structure of Oxa-23 (PDB: 4JF6)[4] consists of the globular α domain and the β-sheet rich β domain. Highlighted lysine residues included K82 (catalytic site), K102, K104, K107 and K194 (homooligomeric sites), and K60, D238 (putative binding interfaces with membrane porins). B) Oxa-23 homodimer model. Model was generated by superimposition of two Oxa-23 monomers 4JF6 to *P. aeruginosa* Oxa-10 dimer (PDB: 1FOF)[5]. Highlighted regions are catalytic site K82 at α3 helix (purple), homodimeric cross-linked sites K194 at α8 helix (red), and K102, K104, K107 at α3-α4 loop (blue). In this dimeric model, the Cα- Cα linear distance of K194-K194 was 5.5 Å, which matched the theoretical maximum Cα- Cα linear distance of BDP-NHP cross-linker of 42.2 Å[6]. However, the Cα- Cα linear distance of K102-K102, K104-K107 and K107-K107 cross-links were larger than 42.2 Å, suggesting the existence of other dimeric conformations or oligomeric forms of Oxa-23. Dimeric structural modeling with Oxa-46 template (PDB: 3IF6)[7] also showed similar results.

A

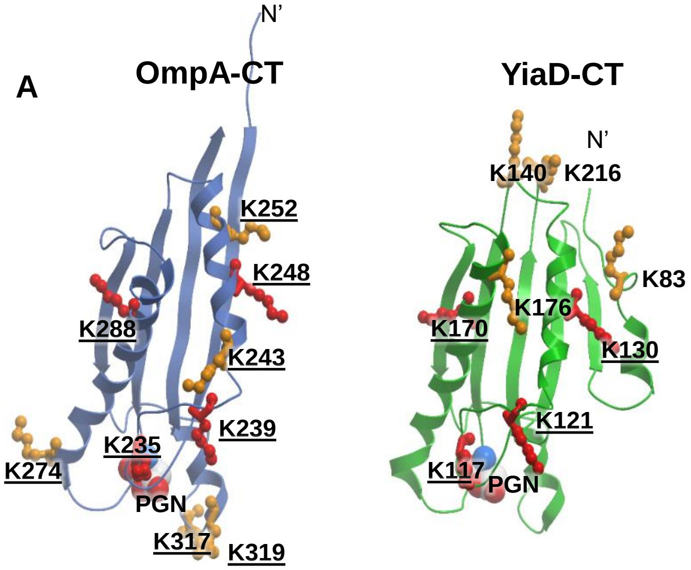

xlinked lysine summary

OmpA-CT	YiaD-CT
n/a	K83
K235	K117
K239	K121
K243	Y125
K248	K130
K252	T134
N258	K140
K274	S156
K288	K170
E294	K176
K317	S198
K319	A200
S335	K216

B

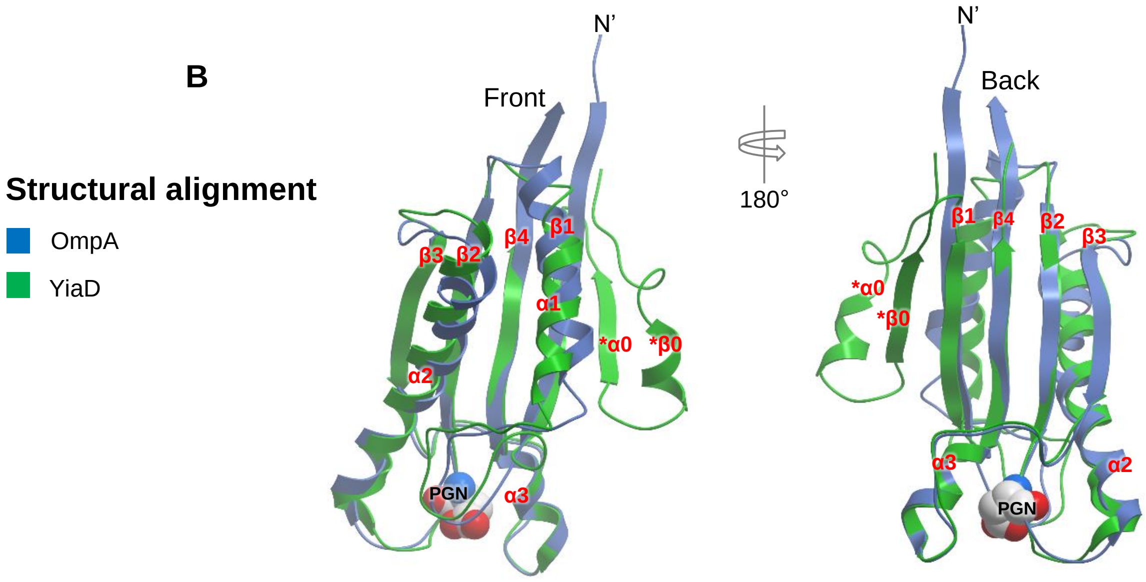

Supplementary Fig. 6 Protein structures and cross-linked sites of OmpA-CT and YiaD-CT domains in AB5075. A) OmpA-CT domain structure 3TD4 and YiaD-CT domain structure based on Phyre2 homology modeling using *P. aeruginosa* TSSL1 template 4B62. The peptidoglycan binding sites[8] (OmpA, D268; YiaD, D150) are highlighted. Protein sequence alignment identified conserved and diverged cross-linked lysines in OmpA-CT and YiaD-CT. Conserved lysines are shown in red and the diverged lysines are shown in orange in the 3D structure. Lysines that were cross-linked to Oxa-23 were underlined. B) Structural alignment of OmpA-CT and YiaD-CT domains using MolSoft BrowserPro (version 3.8-0). Both proteins share a core OmpA-like-CT domain structure of 4 β-strands and 3 α-helices (β1/α1/ β2/α2/ β3/α3/ β4)[8].

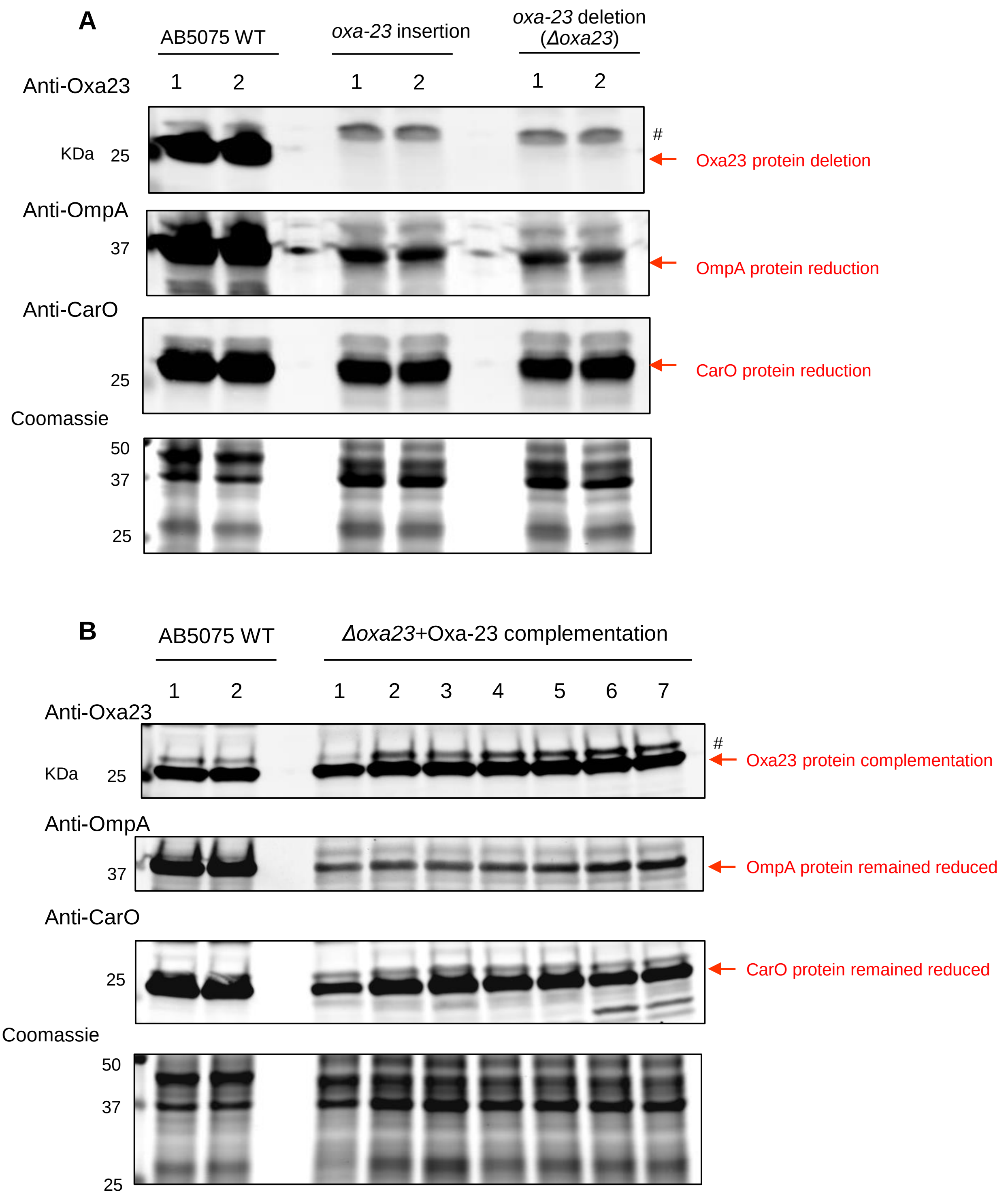

Supplementary Fig. 7 Protein reduction of OmpA and CarO porins due to inactivation of endogenous *oxa-23* gene in AB5075. A) Inactivation of endogenous *oxa-23* gene in AB5075 caused protein reduction of OmpA and CarO porins. B) Complementation of *oxa-23* gene with pMMB.A1 vector in *Δoxa-23* mutant restored the Oxa-23 protein level, but the OmpA and CarO proteins remained reduced in *Δoxa-23* complementation mutants compared to AB5075 WT strain. The # sign indicates a non-specific detection of anti-Oxa23 protein band. This protein band was only apparent in the SDS protein extracts for AB5075 cells, and was not detected in the soluble lysates (Fig. 4A, B, D). Full blots are shown in Supplementary Fig. 16.

A

Membrane porins	Periplasmic loops
OmpA-NT	69LTPWL73, 114NYDSKI119, 157RLNDAL162;
CarO	60NPYV63, 102IRPWGASTNRWAQGL116, 175FAPKINKNW183;
OmpW	59FFGQTPFS66, 101HFKNSTRF108, 148ADAKNW153;
ABUW_2898	60RALSQAKVTHPAQQK74,
Oxa-23	(β1-β2 loop) 58DKK60, (β7-β8 loop) 236QPDGK240

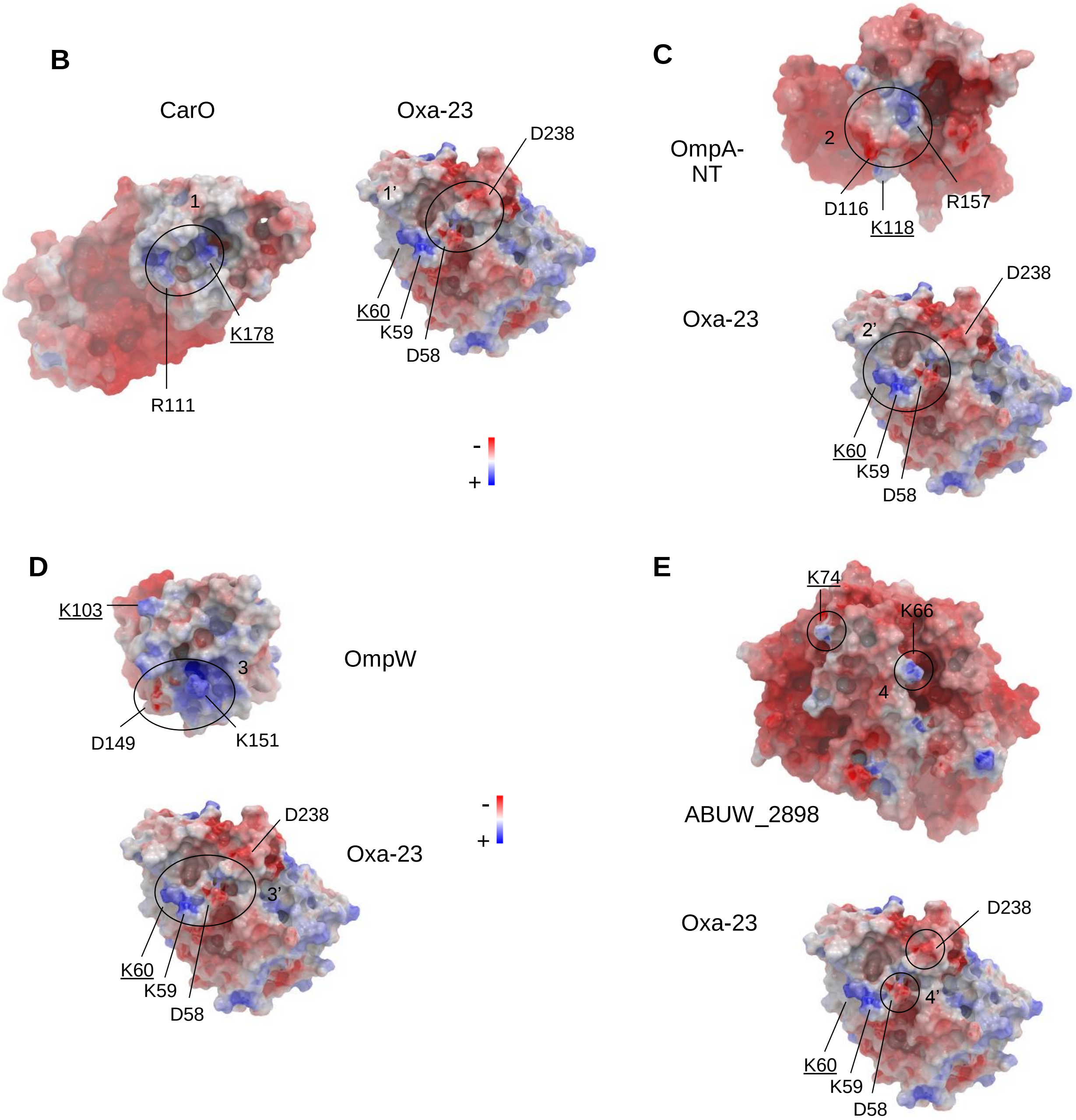

Supplementary Fig. 8 Putative interaction interfaces of Oxa-23 with outer membrane porins. A) Summary table showing residues localized at the periplasmic loops of outer membrane porins OmpA-N-terminal domain, CarO and OmpW, and Oxa-23 β_1-β_2 and β_7-β_8 loops. For ABUW_2898, residues localized at the periplasmic helix bundle[9] are focused. The cross-linked residues are bolded in red, and other charged residues are bolded in blue. B-E) Electrostatic potential surfaces of Oxa-23 and membrane porins, generated with MolSoft BrowserPro (version 3.8-0). Oxa-23 and porins are positioned as shown in Fig 3BC. Interfaces of the top view of Oxa-23, and bottom view of the porins are shown. The 1-4 circles indicate the matched complementary charged residues at β_1-β_2 and β_7-β_8 loops of Oxa-23, and at periplasmic loops of outer membrane porins CarO, OmpA-NT, OmpW and ABUW_2898. The cross-linked lysine pairs are underscored.

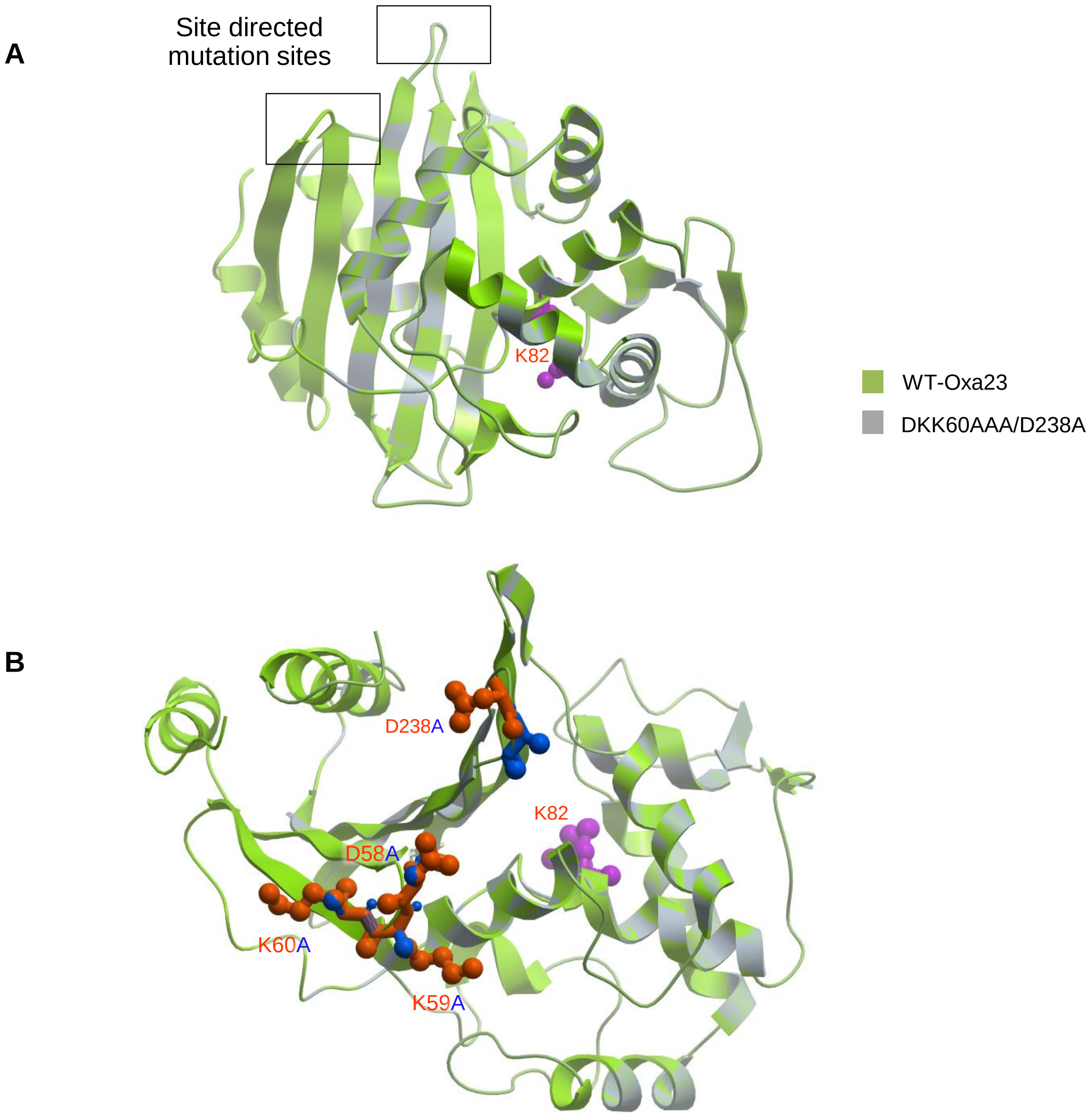

Supplementary Fig. 9 Structural comparison of WT-Oxa23 and DKK60AAA/D238A mutant. A) Structural alignment of WT-Oxa23 (green) and DKK60AAA/D238A (grey). The WT template structure[4] was 4JF6. The mutant structure was obtained with SCWRL4 modeling[10] using the 4JF6 template. The mutated regions at Oxa-23 loops β_1-β_2 and β_7-β_8 are indicated. B) Top view of the Oxa23 putative binding interfaces with porins. The mutated residues DKK60AAA, D238A and the catalytic residue K82 are highlighted.

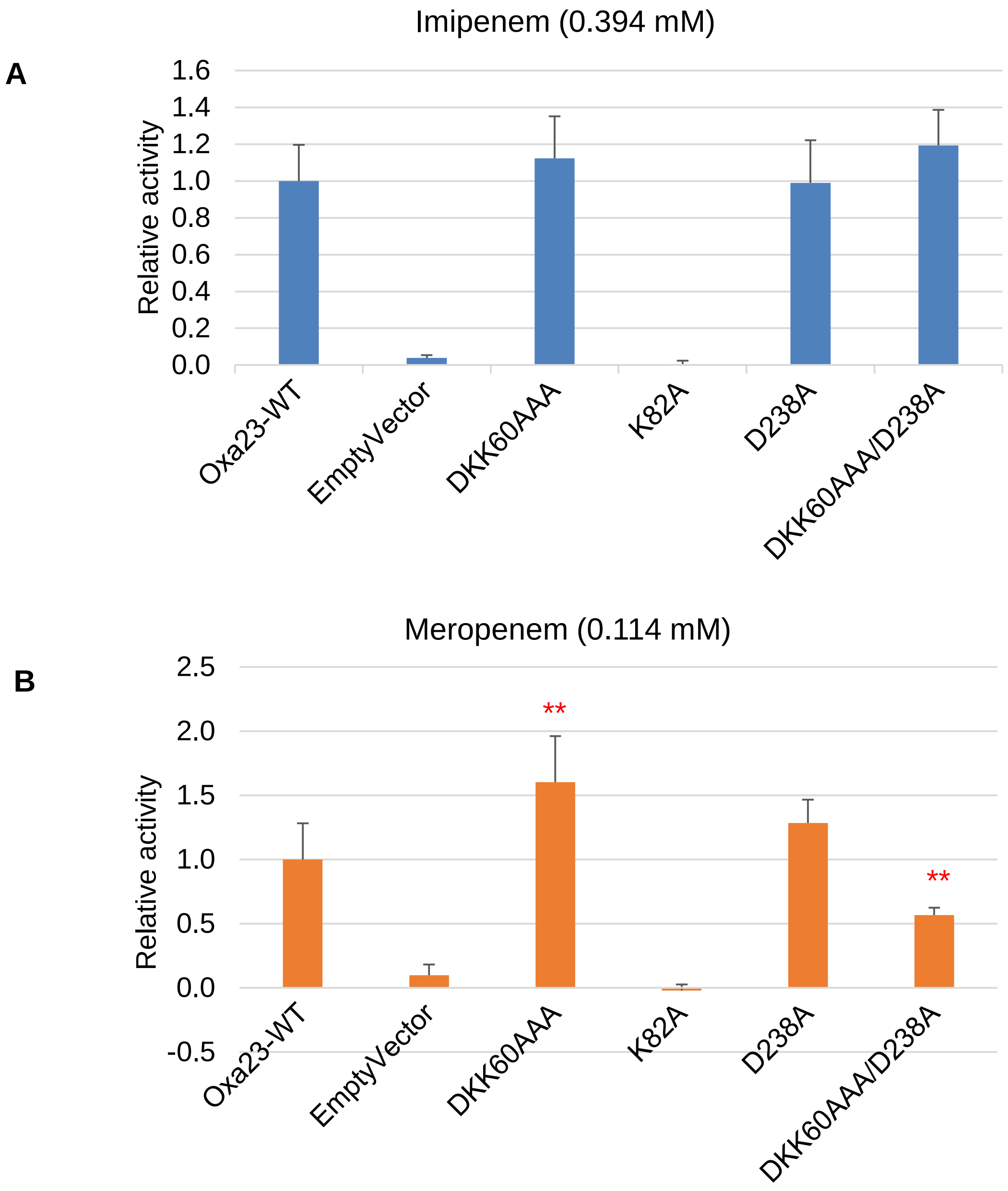

Supplementary Fig. 10 Carbapenem hydrolysis activity of Oxa-23 WT and mutant proteins. The hydrolysis activity of recombinant Oxa-23 proteins expressed with BL21 *E. coli* cells was examined using the spectrophotometric assays as described in Supplemental Methods. Error bars indicate the standard deviation for the two biological replicates (i.e. two independent protein purification events). Each biological replicate contains three technical replicates of enzyme activity measurements. A) Comparison of hydrolysis activity with the substrate of 0.394 mM imipenem. The activity level was normalized to WT Oxa-23. The activity level of Oxa-23 mutants DKK60AAA, D238A and DKK60AAA/D238A was not significantly altered compared to WT Oxa-23. Imipenem hydrolysis activity was not detected in *E. coli* lysates that did not express Oxa-23 enzymes (empty vector) or express Oxa-23 catalytic mutant K82A. B) Comparison of hydrolysis activity with the substrate of 0.114 mM meropenem. The activity level was normalized to WT Oxa-23. DKK60AAA was detected with about 30% increase of activity ($P < 0.01$, one-way ANOVA, n=6), and D238A did not show significant changes, while DKK60AAA/D238A was detected with about 30% decrease of activity ($P < 0.01$). Meropenem hydrolysis activity was not detected in *E. coli* lysates that did not express Oxa-23 enzymes (empty vector) or express Oxa-23 catalytic mutant K82A.

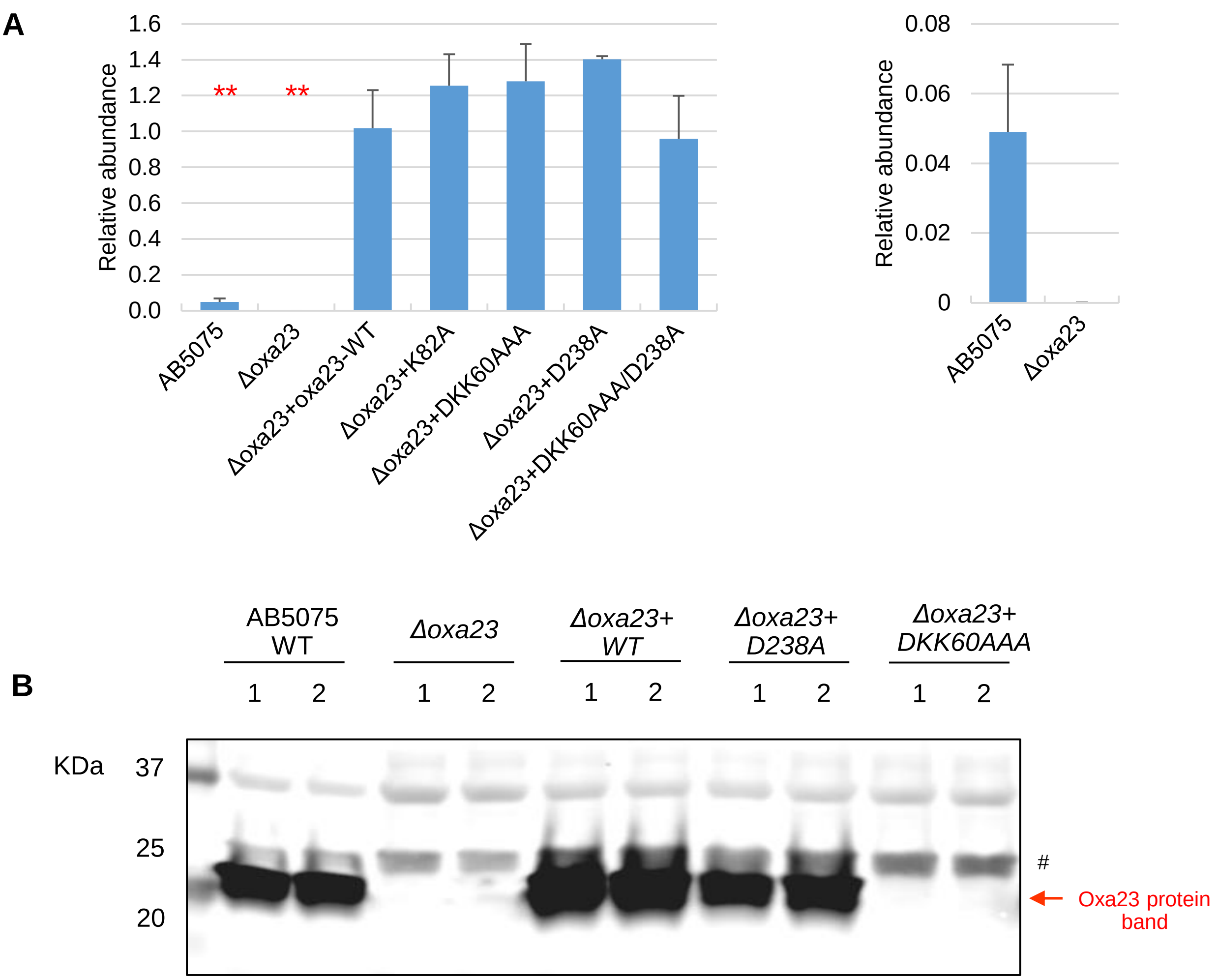

Supplementary Fig. 11 Validation of *oxa-23* expressions with qPCR and immunoblots with *Δoxa23* complementation mutants. A) Quantitative PCR (with *oxa-23* primer set 2) detected equivalent RNA level of *oxa-23* in WT and mutant complementation strains. The RNA levels in the complementation strains were over 20-fold higher compared to the original level in AB5075 WT. Similar results were also obtained with *oxa-23* primer set 1 (Fig. 6AB). Error bars indicate the standard deviation of the three biological replicates examined. Asterisks (**) indicate statistical significance (P<0.01, one-way ANOVA, n=3). B) Anti-Oxa23 antibodies detected decreased Oxa-23 protein abundance in *D238A* complementation strains compared with the *oxa23-WT* complementation strain. Two microgram of proteins were loaded for each lane. Similar results were observed with PRM analyses (Fig. 6C). Note that the DKK60AAA mutations occurred in the epitope region recognized by anti-Oxa23 antibodies, and thus the DKK60AAA mutant was unable to be detected by anti-Oxa23 antibodies. Nevertheless, the abundance level of DKK60AAA proteins could be quantified with PRM assays and compared with Oxa23-WT proteins, using tryptic peptides that do not contain site directed mutations. The # sign indicates a non-specific anti-Oxa23 protein band, as it was not detected with the *Δoxa23* lysate. This protein band was only apparent in the SDS protein extracts for AB5075 cells, and was not detected in the soluble lysates (Fig. 4A, B, D). Full blot image is shown in Supplementary Fig. 17.

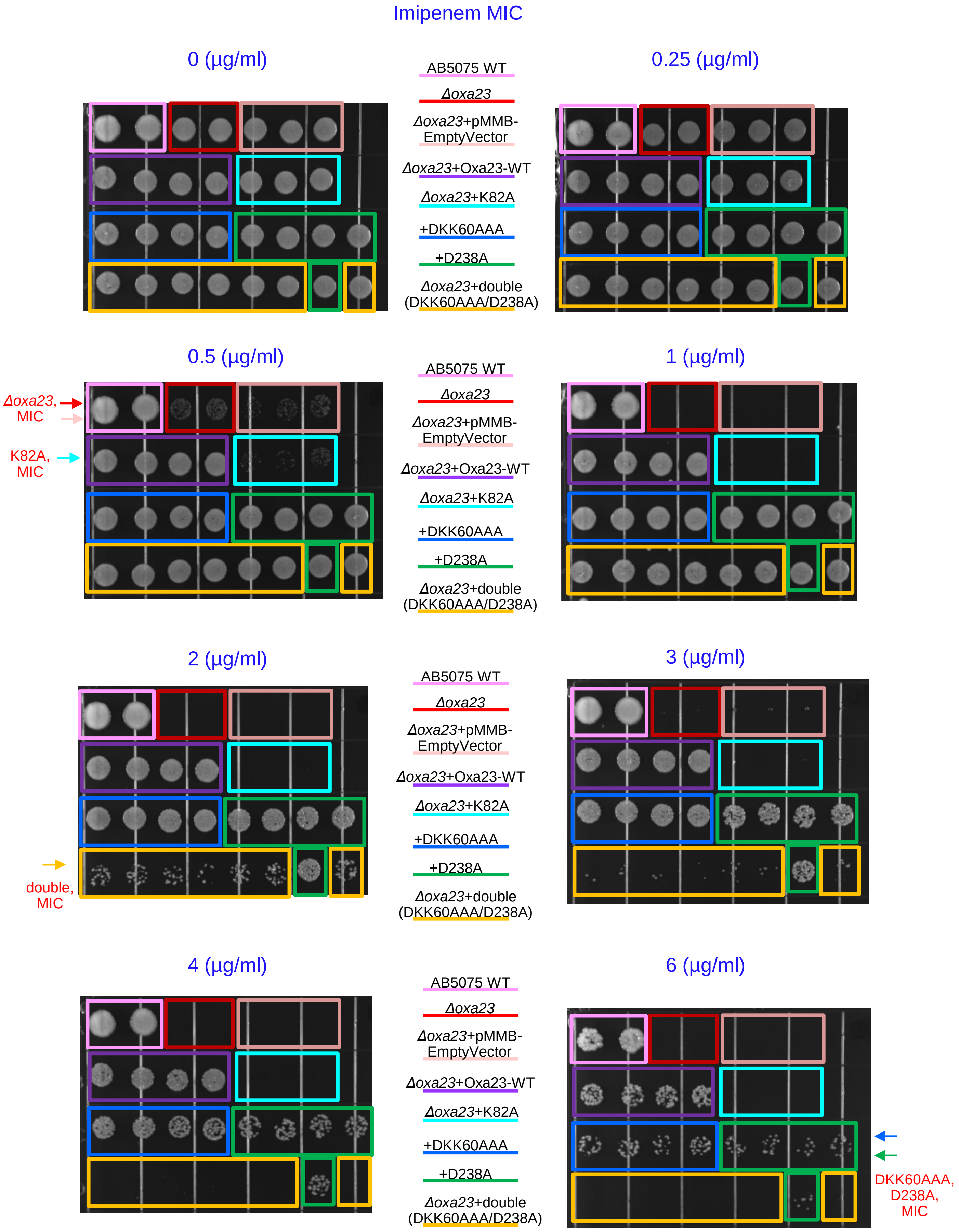

Imipenem MIC
0 (µg/ml)
0.25 (µg/ml)
AB5075 WT
Δoxa23
Δoxa23+pMMB-EmptyVector
Δoxa23+Oxa23-WT
Δoxa23+K82A
+DKK60AAA
+D238A
Δoxa23+double (DKK60AAA/D238A)
0.5 (µg/ml)
1 (µg/ml)
Δoxa23, MIC
K82A, MIC
AB5075 WT
Δoxa23
Δoxa23+pMMB-EmptyVector
Δoxa23+Oxa23-WT
Δoxa23+K82A
+DKK60AAA
+D238A
Δoxa23+double (DKK60AAA/D238A)
2 (µg/ml)
3 (µg/ml)
double, MIC
AB5075 WT
Δoxa23
Δoxa23+pMMB-EmptyVector
Δoxa23+Oxa23-WT
Δoxa23+K82A
+DKK60AAA
+D238A
Δoxa23+double (DKK60AAA/D238A)
4 (µg/ml)
6 (µg/ml)
AB5075 WT
Δoxa23
Δoxa23+pMMB-EmptyVector
Δoxa23+Oxa23-WT
Δoxa23+K82A
+DKK60AAA
+D238A
Δoxa23+double (DKK60AAA/D238A)
DKK60AAA, D238A, MIC

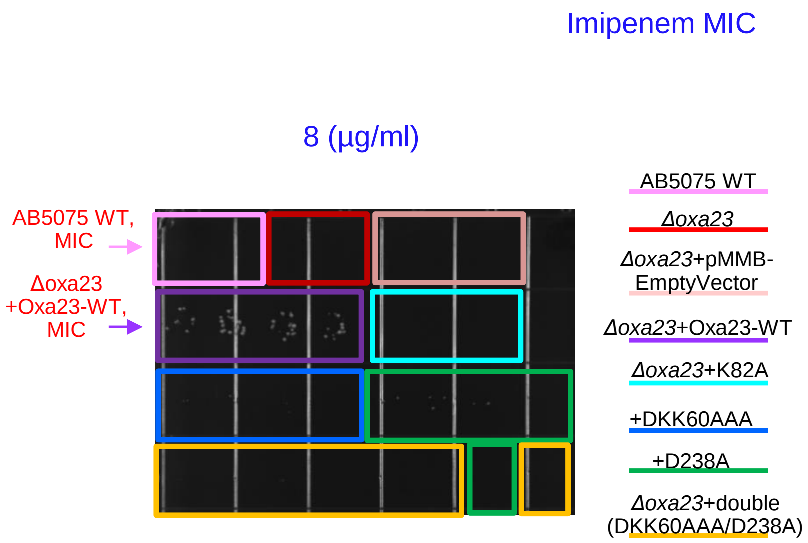

Supplementary Fig. 12 Minimal inhibitory concentration assays of imipenem with AB5075 mutants. Imipenem antibiotic was serially diluted in LB argar plates. Approximately 10^4 cells of overnight cultures were loaded for each spot. Plates were incubated at 37 ^{o}C for 24 h, and the minimal inhibitory concentration (MIC) was the lowest imipenem concentration that prevents the lawn growth of the bacteria. The loading map was kept consistent in all concentrations, as indicated with colored boxes. The MIC for each specific mutant was indicated with arrows in the figures. The double-patch mutants (DKK60AAA/D238A) exhibited 4-fold increase in imipenem sensitivity, compared to the Oxa-23 WT complemented mutant. Thinner lawn growth was observed for single patch mutants (DKK60AAA and D238A) at the 4 µg/ml and 6 µg/ml imipenem concentrations compared to Oxa-23 WT complementation strain.

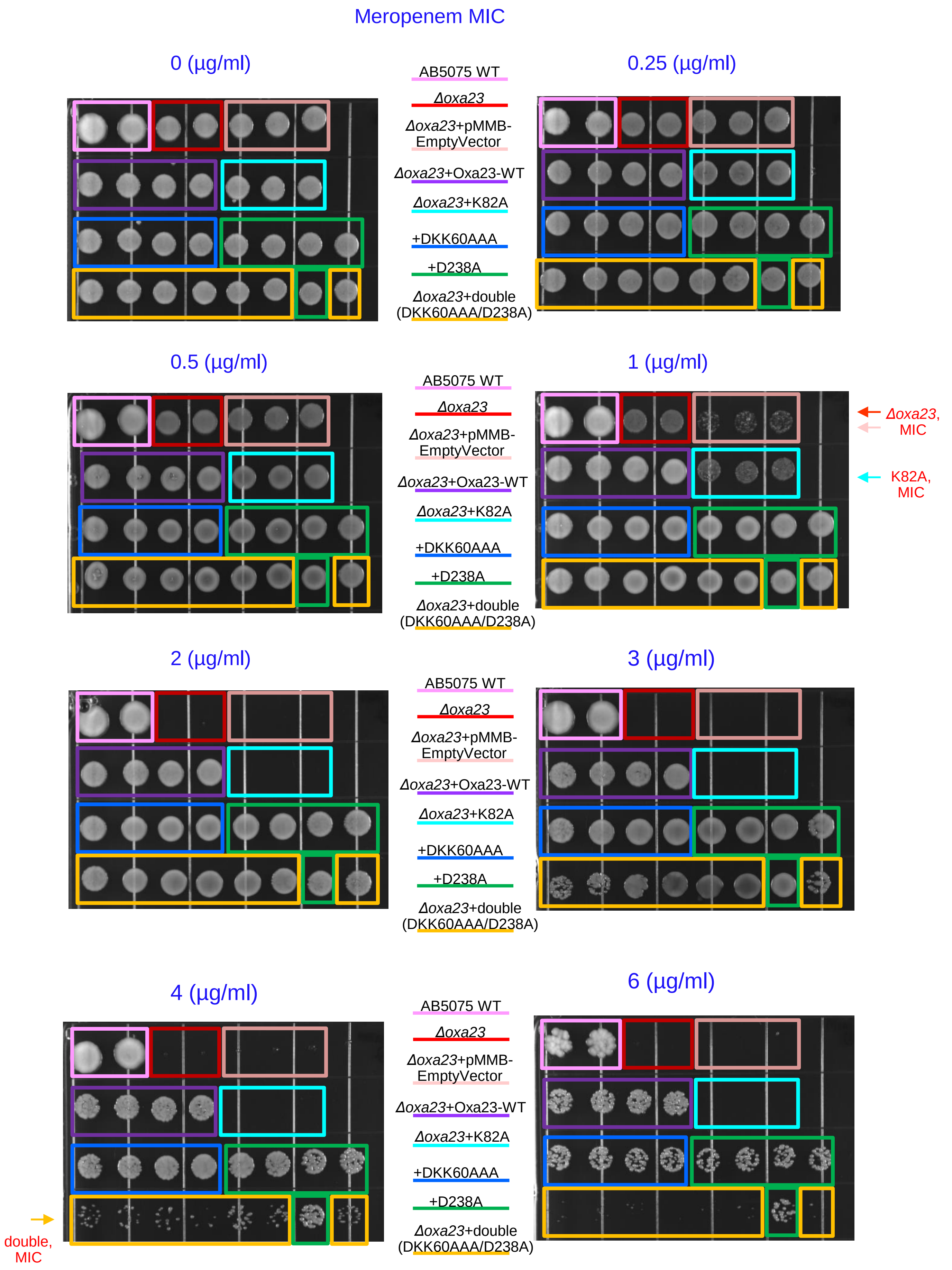
Meropenem MIC
0 (µg/ml)
0.25 (µg/ml)
AB5075 WT
Δoxa23
Δoxa23+pMMB-EmptyVector
Δoxa23+Oxa23-WT
Δoxa23+K82A
+DKK60AAA
+D238A
Δoxa23+double (DKK60AAA/D238A)
0.5 (µg/ml)
1 (µg/ml)
Δoxa23, MIC
K82A, MIC
AB5075 WT
Δoxa23
Δoxa23+pMMB-EmptyVector
Δoxa23+Oxa23-WT
Δoxa23+K82A
+DKK60AAA
+D238A
Δoxa23+double (DKK60AAA/D238A)
2 (µg/ml)
3 (µg/ml)
AB5075 WT
Δoxa23
Δoxa23+pMMB-EmptyVector
Δoxa23+Oxa23-WT
Δoxa23+K82A
+DKK60AAA
+D238A
Δoxa23+double (DKK60AAA/D238A)
4 (µg/ml)
6 (µg/ml)
AB5075 WT
Δoxa23
Δoxa23+pMMB-EmptyVector
Δoxa23+Oxa23-WT
Δoxa23+K82A
+DKK60AAA
+D238A
Δoxa23+double (DKK60AAA/D238A)
double, MIC

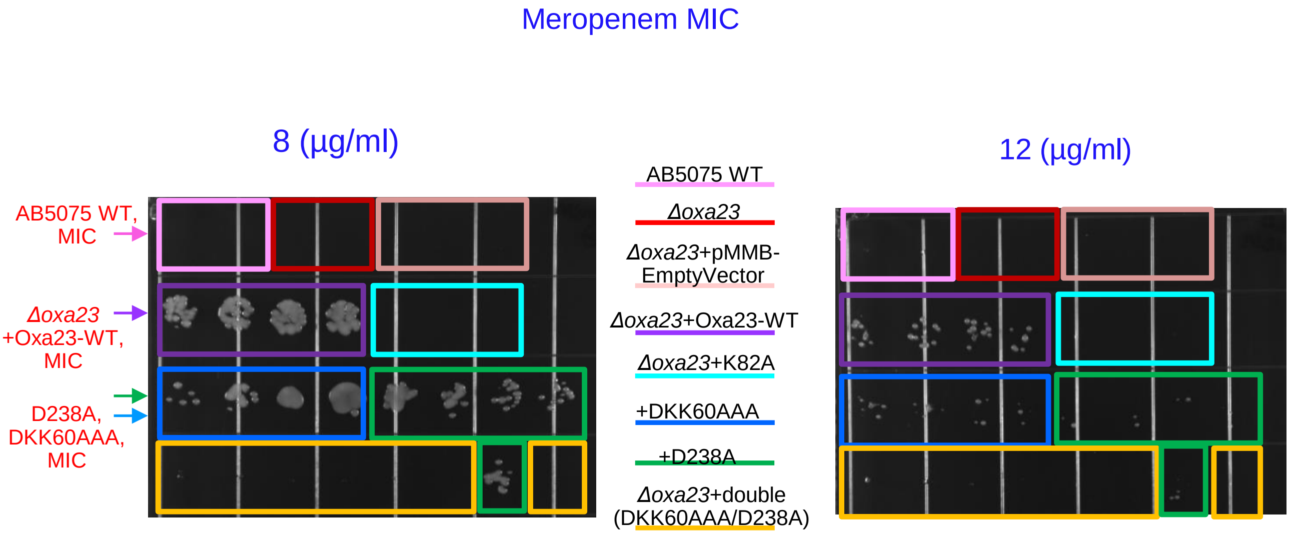

Supplementary Fig. 13 Minimal inhibitory concentration assays of meropenem with AB5075 mutants. Meropenem antibiotic was serially diluted in LB argar plates. Approximately 10^4 cells of the overnight cultures were loaded for each spot. Plates were incubated at 37ºC for 24 h, and the minimal inhibitory concentration (MIC) was the lowest meropenem concentration that prevents the lawn growth of the bacteria. The loading map was kept consistent in all concentrations, as indicated with colored boxes. The MIC for each specific mutant was indicated with arrows in the figures. The double patch mutants (DKK60AAA/D238A) exhibited 2-fold increase in meropenem sensitivity, compared to the Oxa-23 WT complemented mutant. Thinner lawn growth was observed for single patch mutants (DKK60AAA and D238A) at the 6 µg/ml meropenem concentration compared to Oxa-23 WT complementation strain.

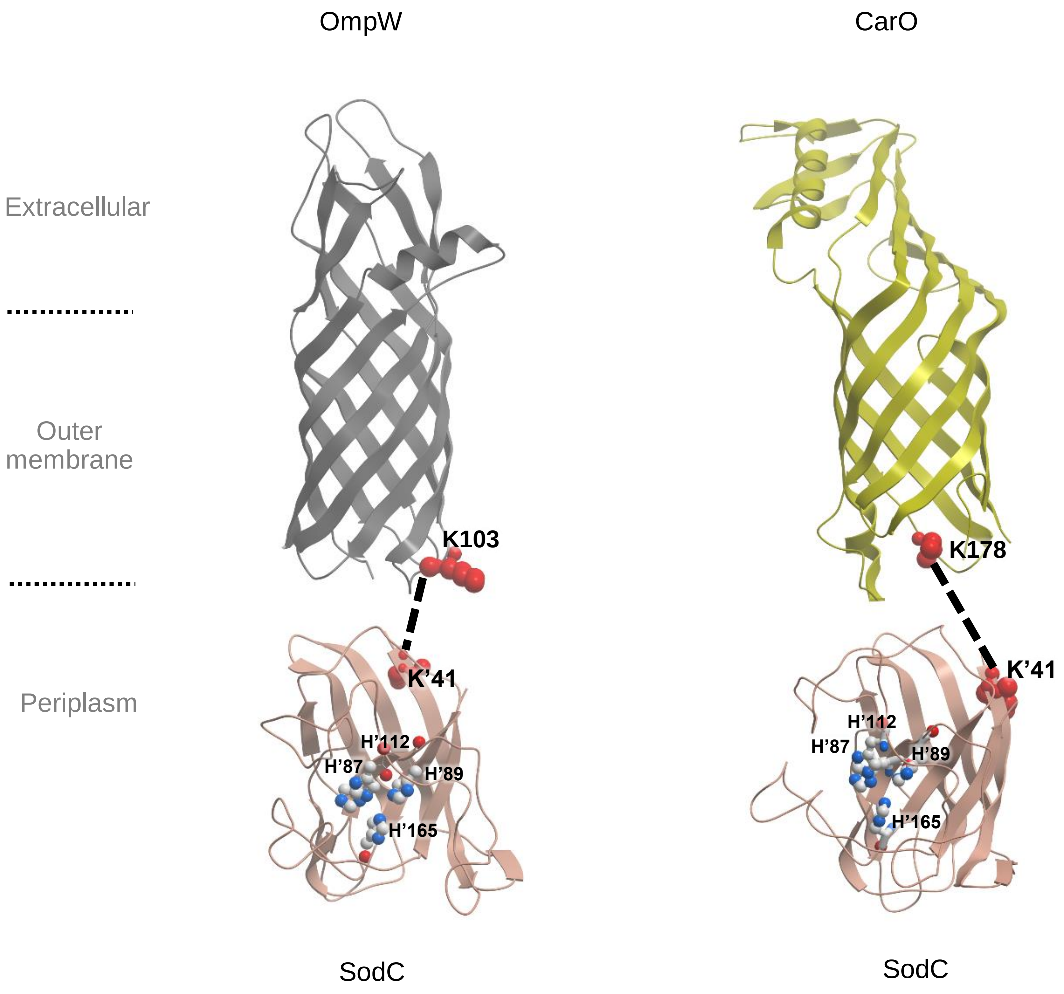

Supplementary Fig. 14 Superoxide dismutase (SodC) interactions with membrane porins OmpW and CarO. *A. baumannii* SodC structural model was obtained with Phyre2 modeling using *E. coli* template (PDB: 1ESO)[11]. Cross-linked residues SodC-OmpW (K'41-K103) and SodC-CarO (K'41-R178), and the conserved copper binding sites H'87, H'89, H'112, and H'165 in SodC are highlighted.

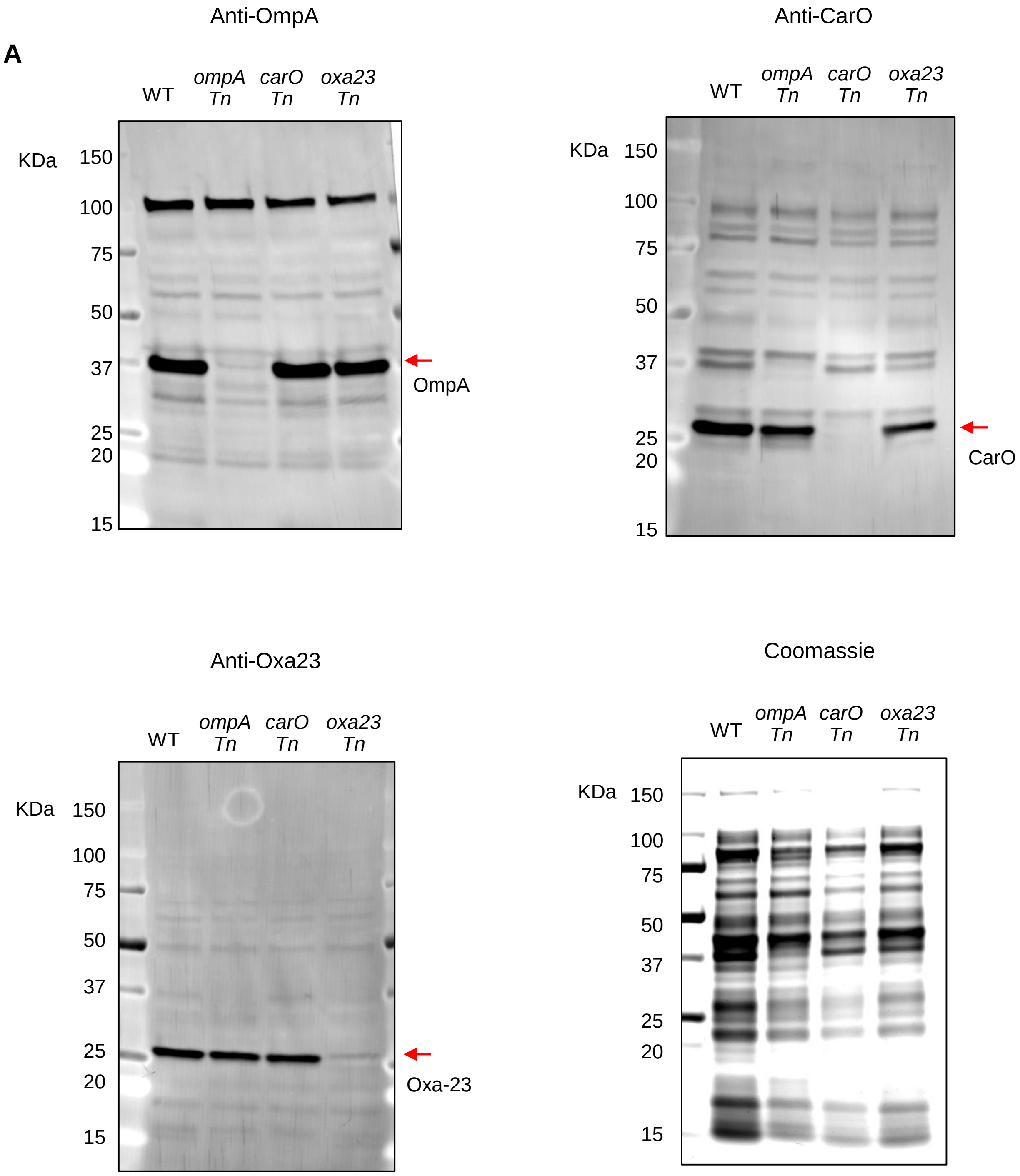
A
Anti-OmpA
WT
ompA Tn
carO Tn
oxa23 Tn
KDa
150
100
75
50
37
25
20
15
OmpA
Anti-CarO
WT
ompA Tn
carO Tn
oxa23 Tn
KDa
150
100
75
50
37
25
20
15
CarO
Anti-Oxa23
WT
ompA Tn
carO Tn
oxa23 Tn
KDa
150
100
75
50
37
25
20
15
Oxa-23
Coomassie
WT
ompA Tn
carO Tn
oxa23 Tn
KDa
150
100
75
50
37
25
20
15

B

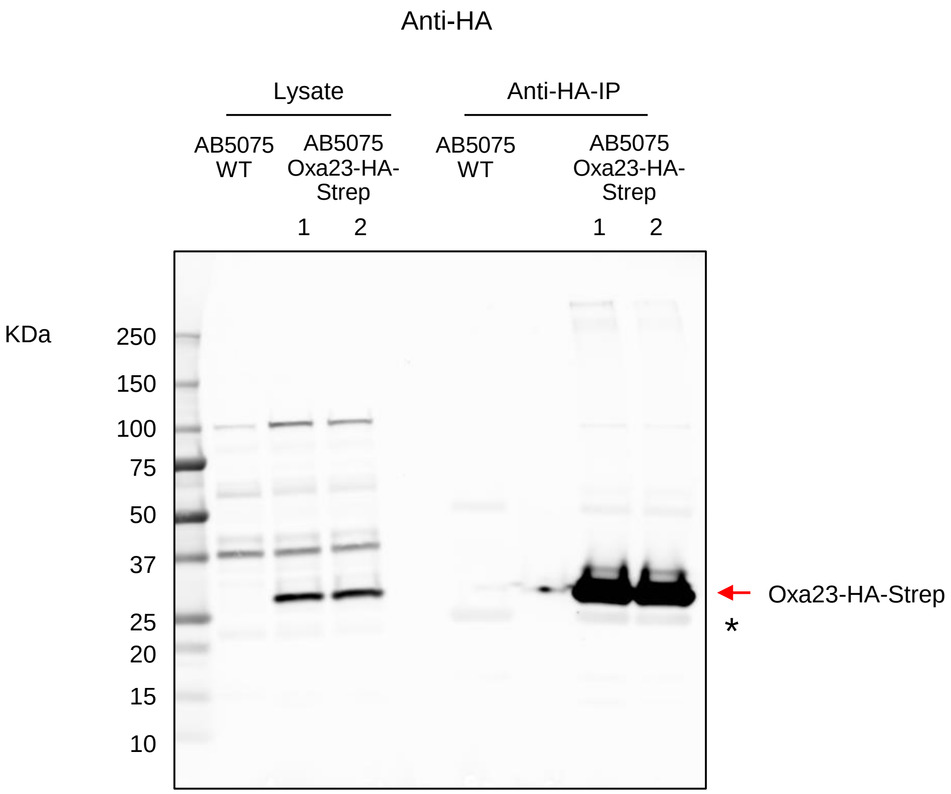

Anti-HA
Lysate
Anti-HA-IP
AB5075 WT
AB5075 Oxa23-HA-Strep
AB5075 WT
AB5075 Oxa23-HA-Strep
1
2
1
2
KDa
250
150
100
75
50
37
25
20
15
10
Oxa23-HA-Strep
*

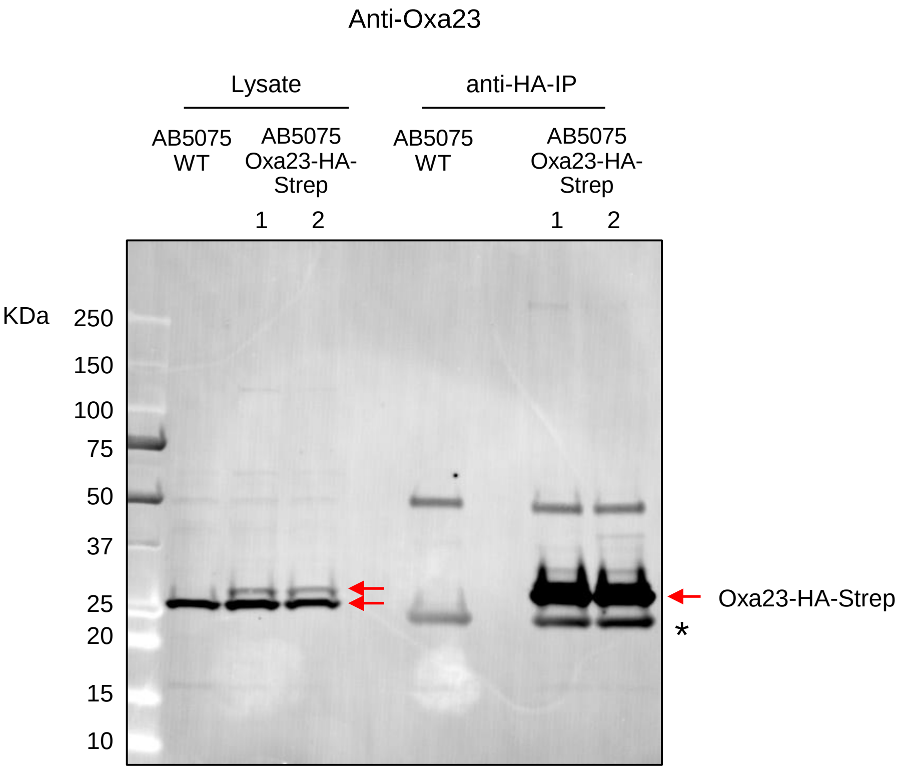

Anti-Oxa23
Lysate
anti-HA-IP
AB5075 WT
AB5075 Oxa23-HA-Strep
AB5075 WT
AB5075 Oxa23-HA-Strep
1
2
1
2
KDa
250
150
100
75
50
37
25
20
15
10
Oxa23-HA-Strep
*

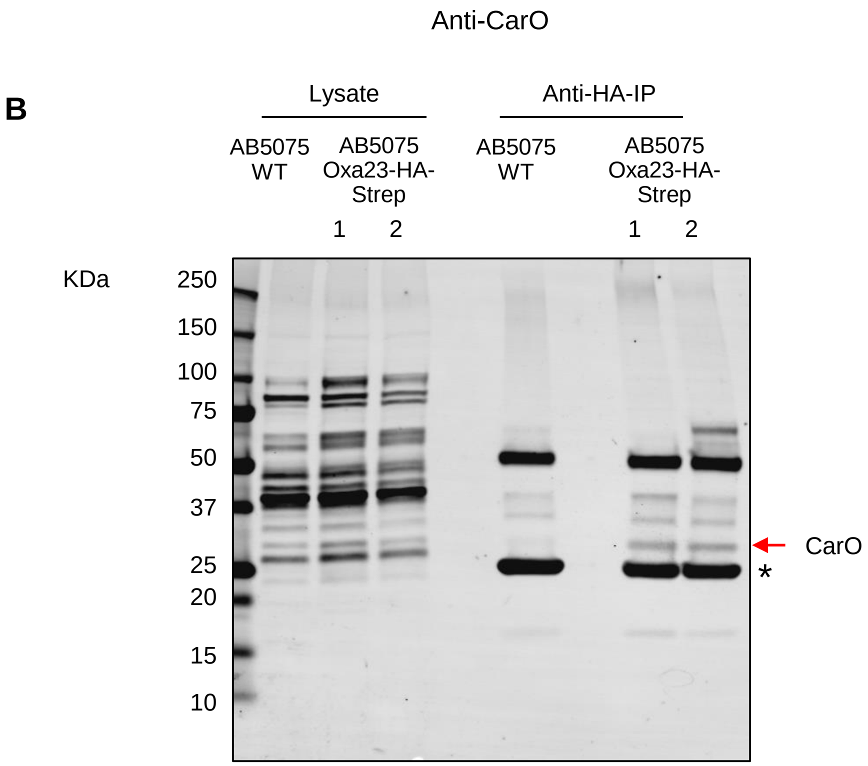
Anti-CarO
B
Lysate
Anti-HA-IP
AB5075 WT
AB5075 Oxa23-HA-Strep
AB5075 WT
AB5075 Oxa23-HA-Strep
1
2
1
2
KDa
250
150
100
75
50
37
25
20
15
10
CarO
*

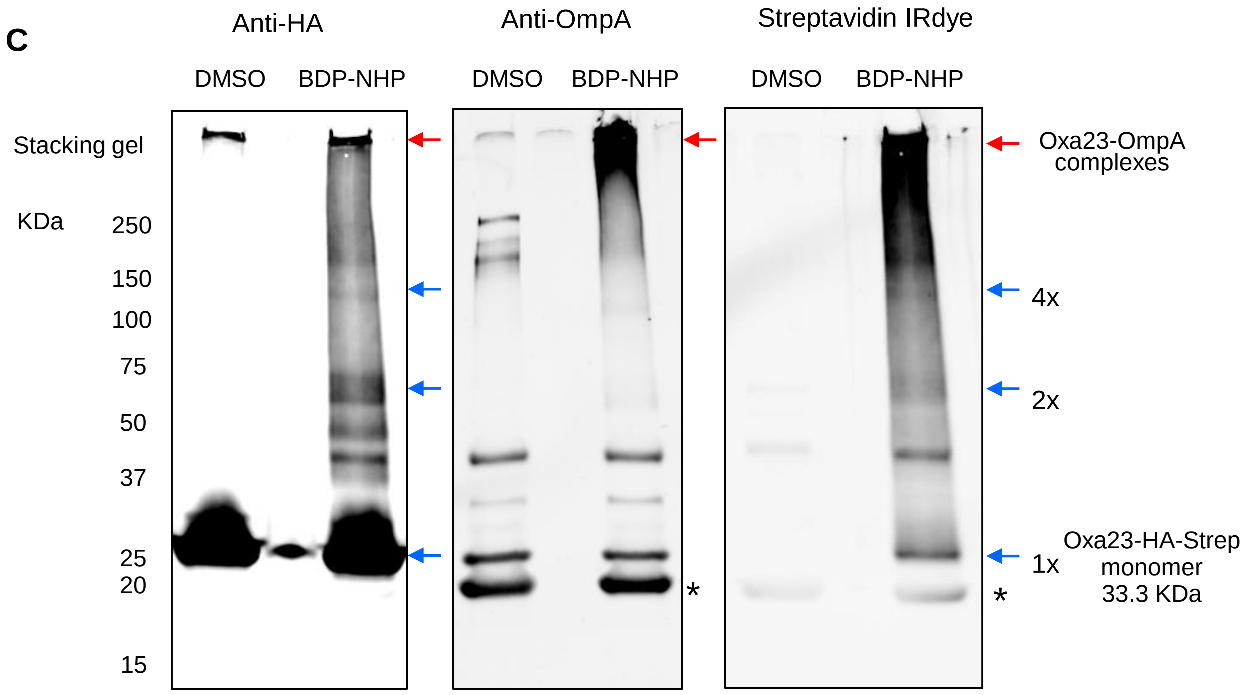
C
Anti-HA
Anti-OmpA
Streptavidin IRdye
DMSO
BDP-NHP
DMSO
BDP-NHP
DMSO
BDP-NHP
Stacking gel
KDa
250
150
100
75
50
37
25
20
15
Oxa23-OmpA complexes
4x
2x
1x
Oxa23-HA-Strep monomer 33.3 KDa
*
*

D

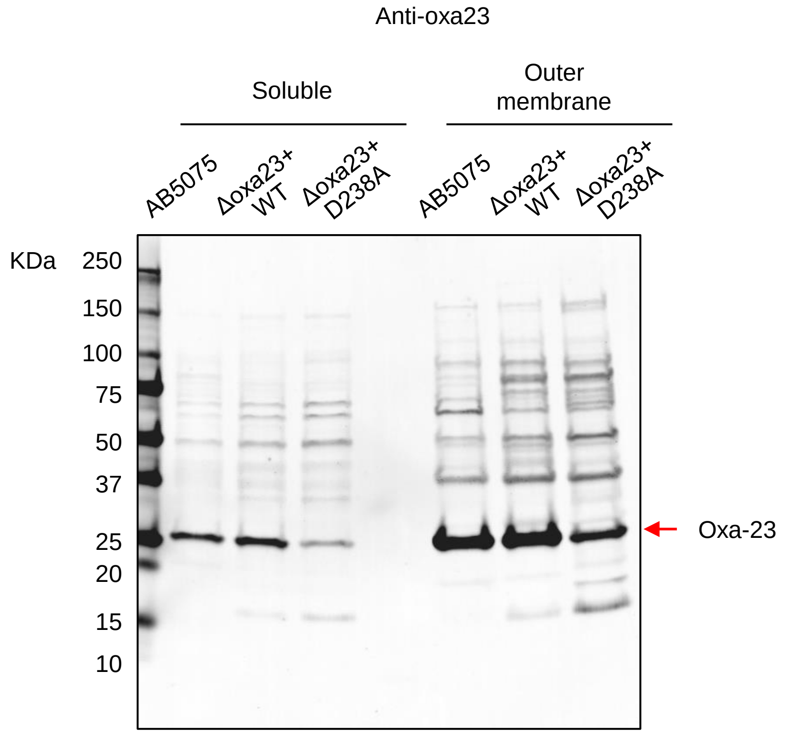

Anti-oxa23
Soluble
Outer membrane
AB5075
Δoxa23+ WT
Δoxa23+ D238A
AB5075
Δoxa23+ WT
Δoxa23+ D238A
KDa
250
150
100
75
50
37
25
20
15
10
Oxa-23

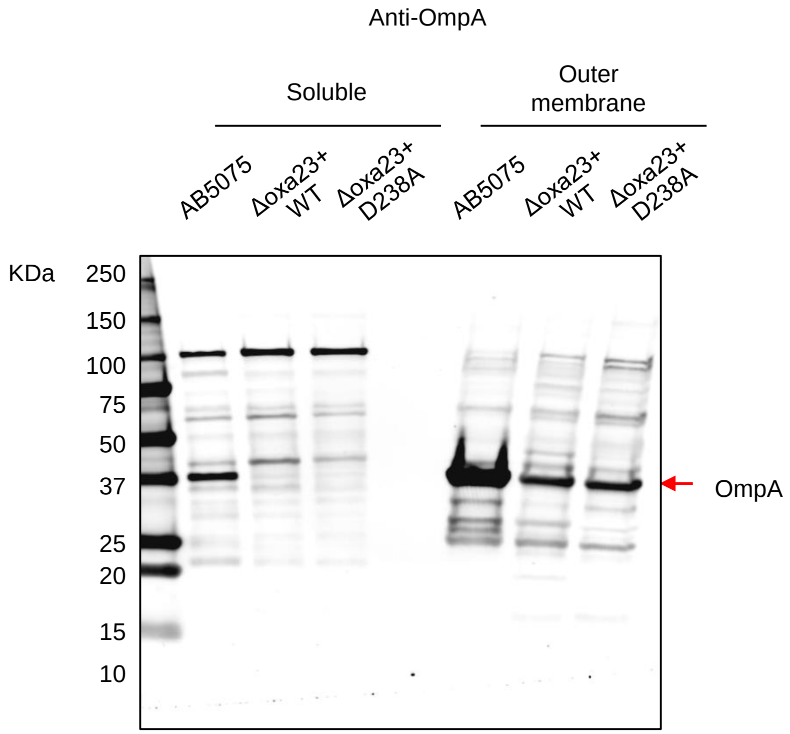

Anti-OmpA
Soluble
Outer membrane
AB5075
Δoxa23+ WT
Δoxa23+ D238A
AB5075
Δoxa23+ WT
Δoxa23+ D238A
KDa
250
150
100
75
50
37
25
20
15
10
OmpA

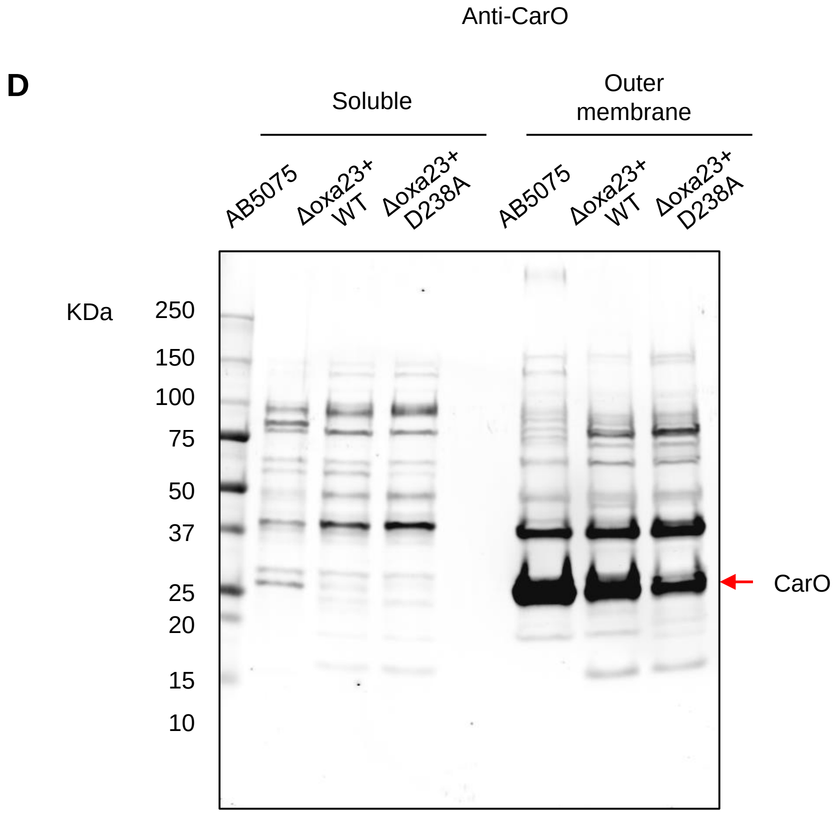

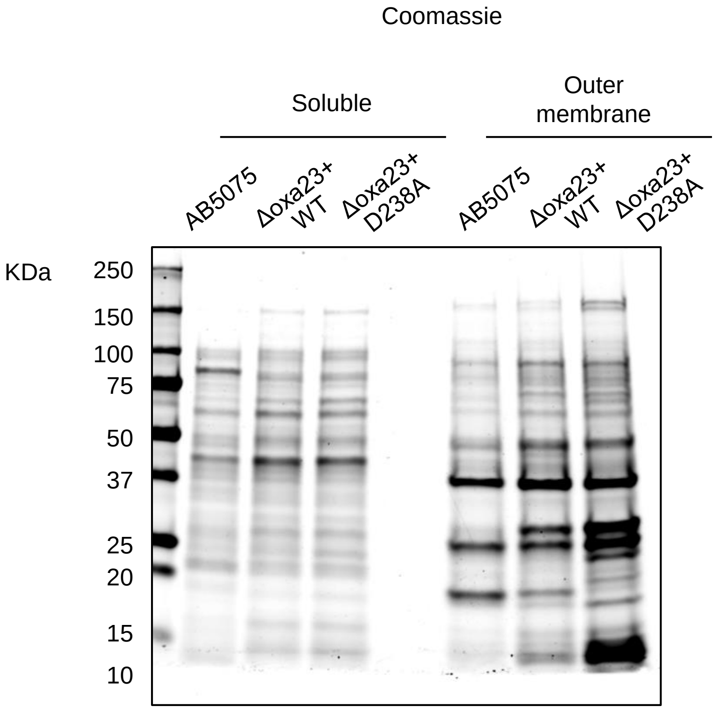

Supplementary Fig. 15 Full blots for the immunoblots shown in Fig. 4. Here letters "A-D" correspond to panels A-D in Fig. 4.

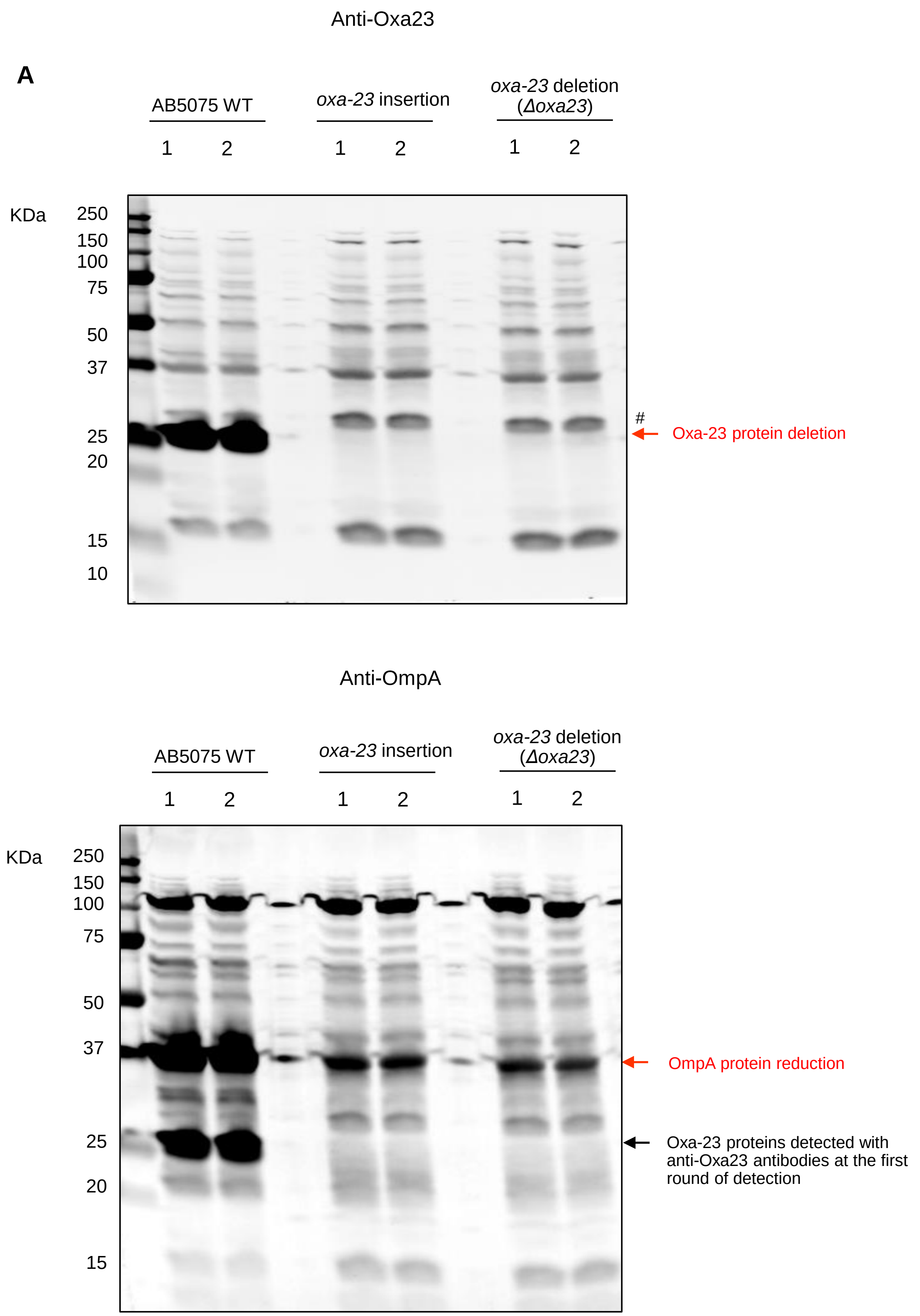

Anti-Oxa23
A
AB5075 WT
oxa-23 insertion
oxa-23 deletion (Δoxa23)
1
2
1
2
1
2
KDa
250
150
100
75
50
37
25
20
15
10
#
Oxa-23 protein deletion
Anti-OmpA
AB5075 WT
oxa-23 insertion
oxa-23 deletion (Δoxa23)
1
2
1
2
1
2
KDa
250
150
100
75
50
37
25
20
15
OmpA protein reduction
Oxa-23 proteins detected with anti-Oxa23 antibodies at the first round of detection

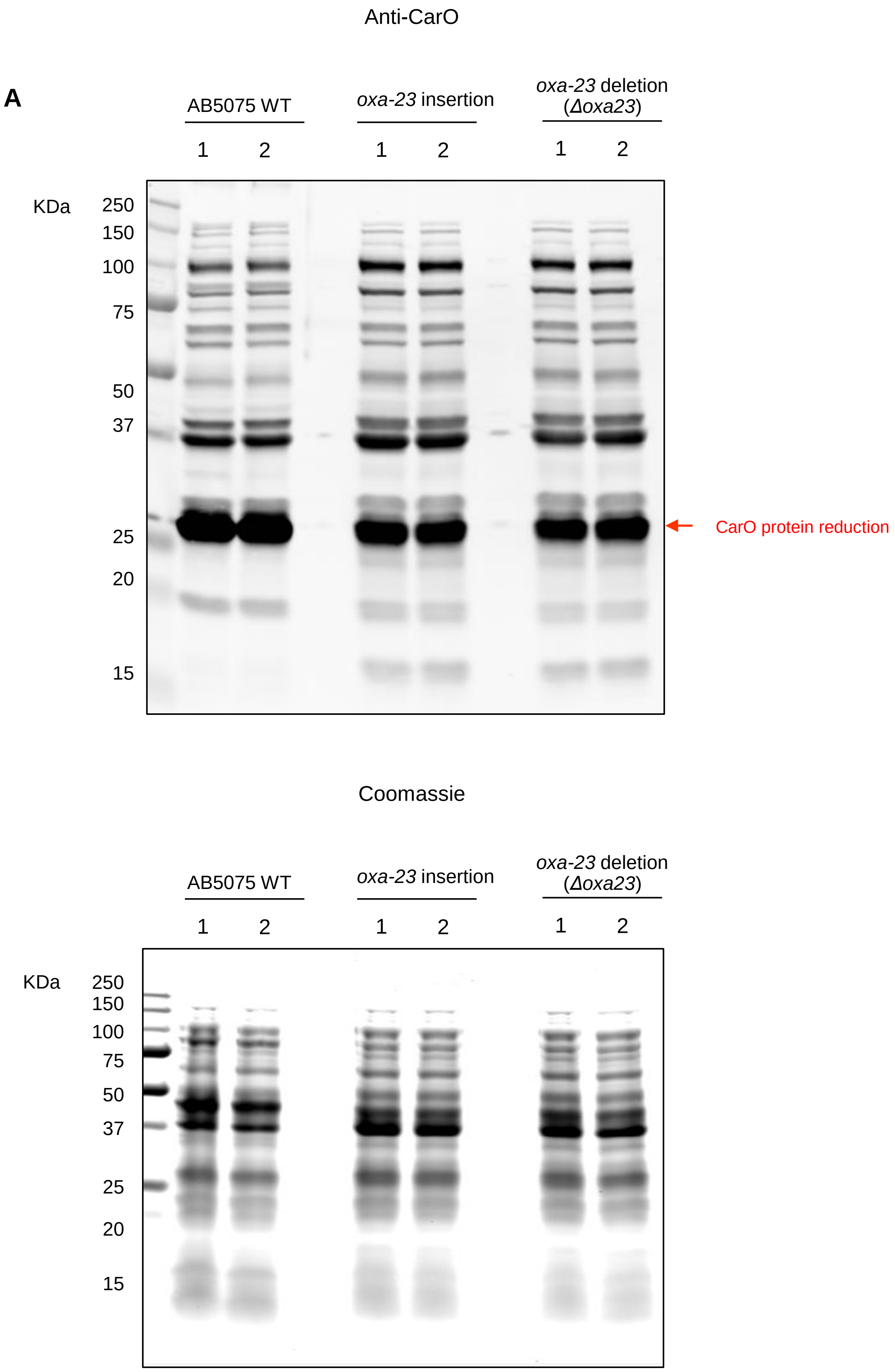
Anti-CarO
A
AB5075 WT
oxa-23 insertion
oxa-23 deletion (Δoxa23)
1 2 1 2 1 2
KDa
250
150
100
75
50
37
25
20
15
CarO protein reduction
Coomassie
AB5075 WT
oxa-23 insertion
oxa-23 deletion (Δoxa23)
1 2 1 2 1 2
KDa
250
150
100
75
50
37
25
20
15

B

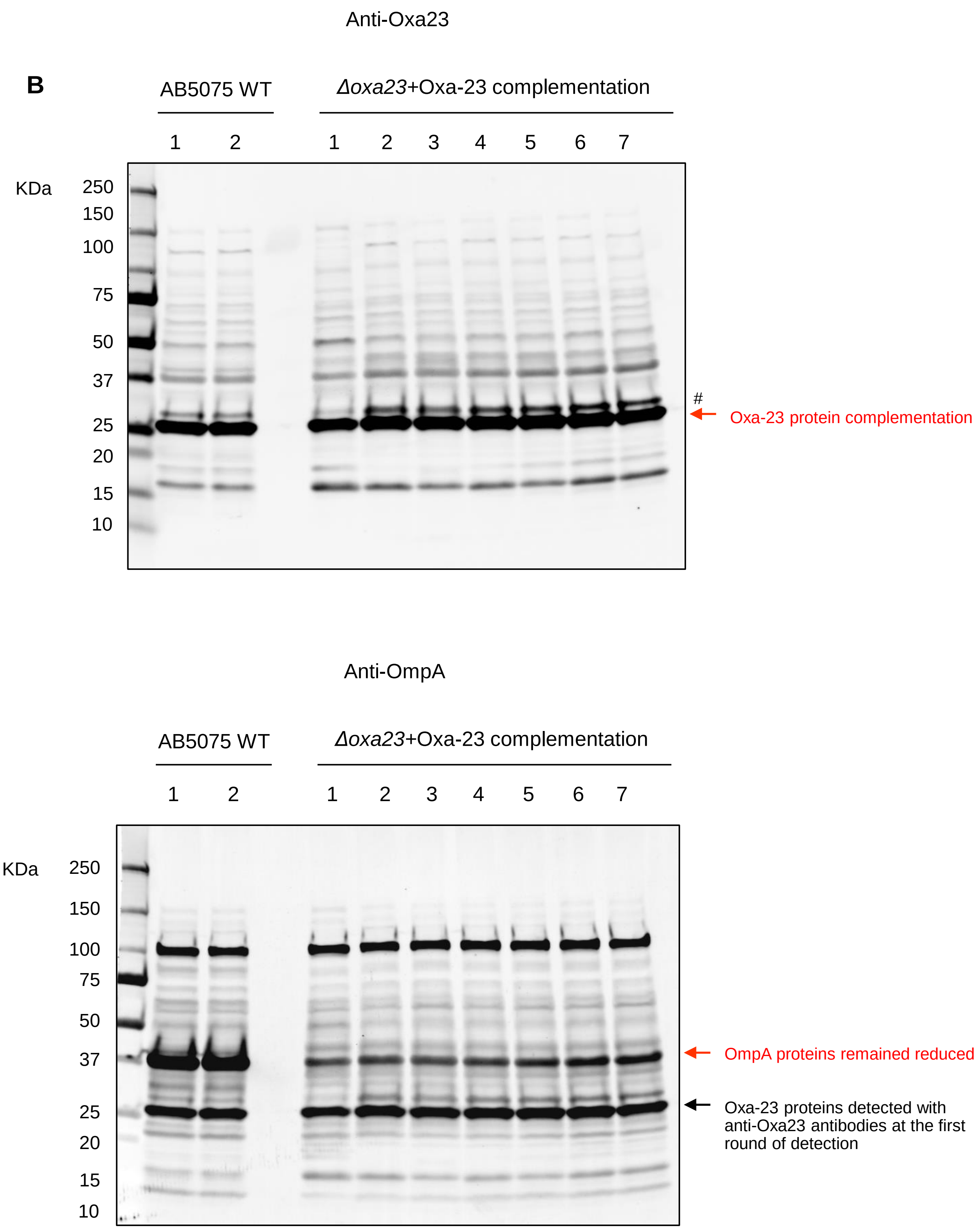
Anti-Oxa23
AB5075 WT
Δoxa23+Oxa-23 complementation
1 2
1 2 3 4 5 6 7
KDa
250
150
100
75
50
37
25
20
15
10
#
Oxa-23 protein complementation
Anti-OmpA
AB5075 WT
Δoxa23+Oxa-23 complementation
1 2
1 2 3 4 5 6 7
KDa
250
150
100
75
50
37
25
20
15
10
OmpA proteins remained reduced
Oxa-23 proteins detected with anti-Oxa23 antibodies at the first round of detection

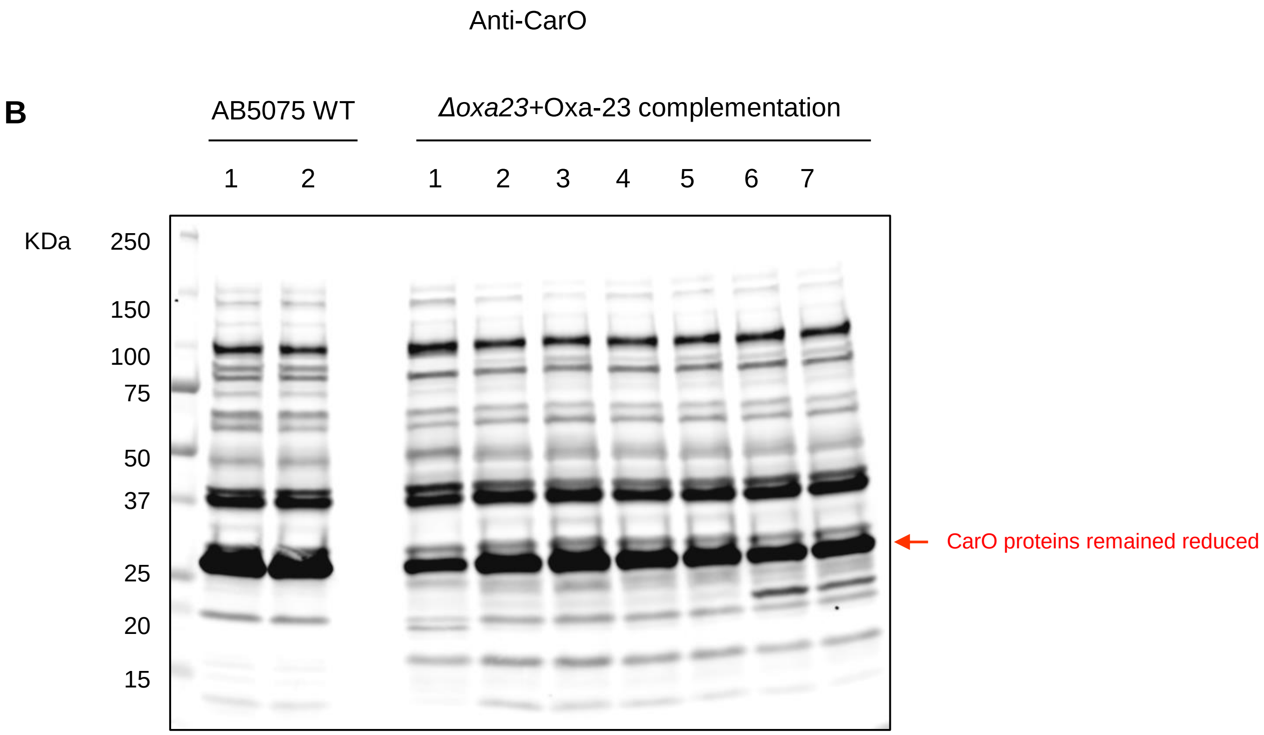

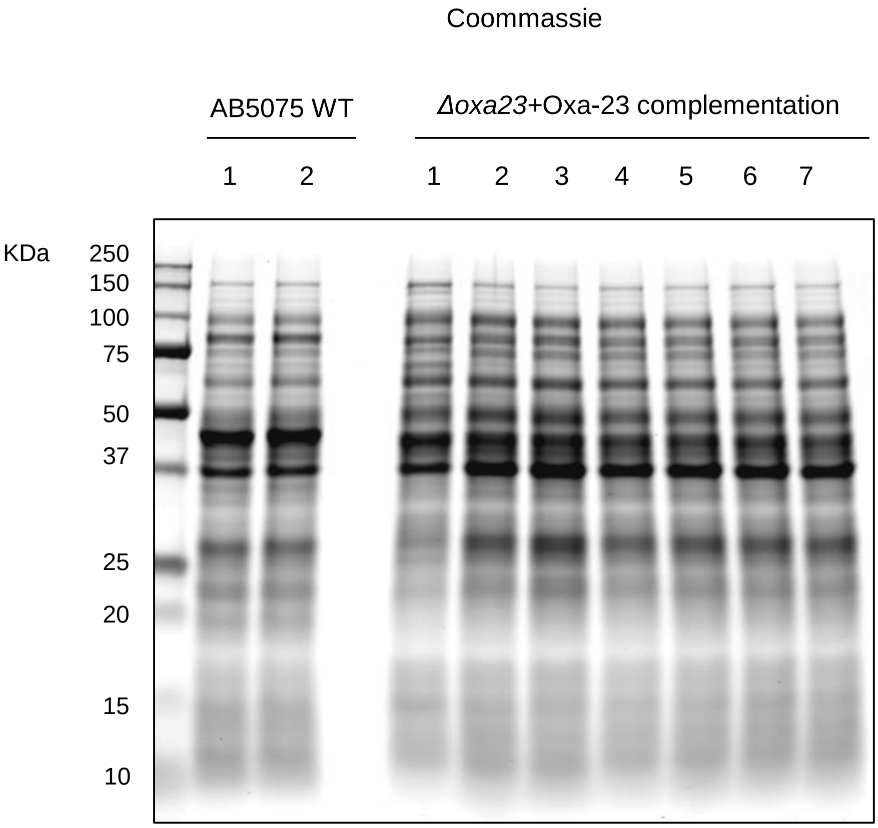

Supplementary Fig. 16 Full blots for the immunoblots shown in Supplementary Fig. 7. Here letters “A” or “B” correspond to panels A or B in Supplementary Fig. 7.

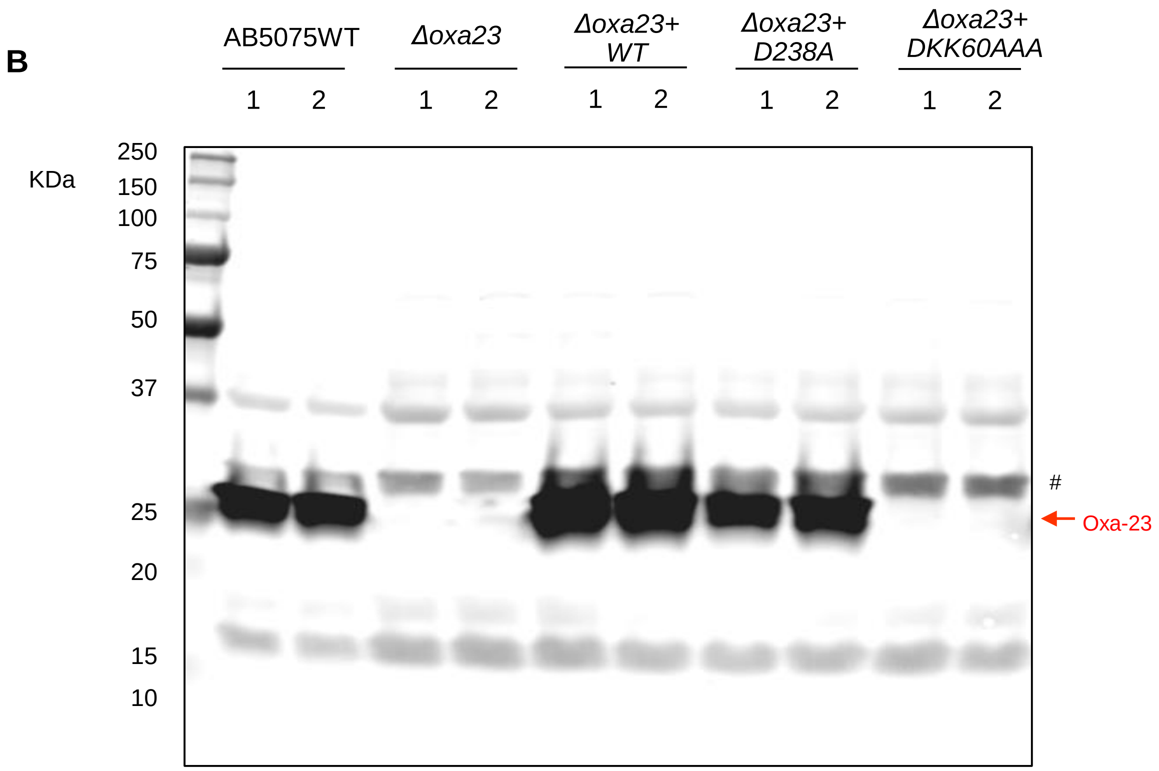

Supplementary Fig. 17 Full blot for the immunoblot shown in Supplementary Fig. 11. Here "B" corresponds to panel B in Supplementary Fig. 11.

Supplementary Table 1   Summary of plasmids and strains used in this study

**Plasmids**	**Generated Strains**	**Source or Reference**	**Application**
	AB5075	12,13	A carbapenem resistant strain, isolated from a patient with tibia osteomyelitis. It is the parent strain for transposon mutants and *Δoxa23* deletion and complementation mutants.
	AB5075 transposon mutants<br>*oxa-23 Tn*<br>*carO Tn*<br>*ompA Tn*<br>*ampC Tn*<br>*oxa-69 Tn*<br>*ompW Tn*<br>*yiaD Tn*<br>*ABUW_2898 Tn*<br>*ABUW_0724 Tn*	14	To study the carbapenem sensitivity due to gene inactivation. Here, *oxa-23* and protein interactors of Oxa-23 are focused.
pMMB-HA-Strep	AB5075 pMMB-*oxa23*-HA-Strep	15, this study	Producing Oxa-23 proteins with the C-terminal HA-Strep tag in AB5075 cells that allows co-IP analysis with Oxa-23 proteins. The pMMB-HA-Strep plasmid contains the lactose-dependent promoter.
pSL15A	*Δoxa23*	this study	Generation of *Δoxa23* deletion strain.
pMMB.A1	*Δoxa23*+pMMB.A1-*oxa23*-WT<br>*Δoxa23*+pMMB.A1-*oxa23*-K82A<br>*Δoxa23*+pMMB.A1-*oxa23*-DKK60AAA<br>*Δoxa23*+pMMB.A1-*oxa23*-D238A<br>*Δoxa23*+pMMB.A1-*oxa23*-DKKK60AAA/D238A	this study	Complementation analysis of *Δoxa23* deletion mutant with *oxa-23* or site-directed mutants of *oxa-23*. pMMB.A1 plasmid contains the constitutive expression promoter.
	AB19606	American Type Culture Collection (ATCC)	A control strain of a carbapenem sensitive strain.
pET-28b(+)	BL21 pET-28b-*oxa23*-WT<br>BL21 pET-28b-*oxa23*-K82A<br>BL21 pET-28b-*oxa23*-DKK60AAA<br>BL21 pET-28b-*oxa23*-D238A<br>BL21 pET-28b-*oxa23*-DKK60AAA/D238A	Novagen	Production of recombinant Oxa-23 proteins with BL21 *E. coli* cells.

Supplementary Methods

Synthesis of chemical cross-linkers

The PIR cross-linker Biotin Aspartyl Prolyl N-hydroxyphthalamide (BDP-NHP) was synthesized as previously described[16, 17]. Briefly, synthesis was performed with solid phase peptide synthesis method and fluorenylmethyloxycarbonyl (Fmoc) chemistry using an Endeavor 90 Peptide Synthesizer (AAPPTec, Louisville, KY, USA). Fmoc-Gly-SASRIN resin (Bachem, Torrance, CA, USA) was the solid support and amino acids (biotin)-Lys, Lys, Pro and Asp were sequentially coupled. Reaction yields were measured through the absorbance of released Fmoc at 307 nm with a cumulative yield of > 90%. To incorporate the reactive groups in the cross-linkers, succinic anhydride was coupled to the C-termini of the Asp residues and activated with TFA-NHP to form N-hydroxyphthalamide (NHP) ester reactive to primary amines[18]. The synthesized cross-linker was cleaved off the solid support with 95% trifluoroacetic acid 5% dichoromethane, and this step also helped remove the N-tert-butoxycarbonyl (t-Boc) protecting groups from Asp side chains. NHP-BDP was then purified with diethyl ether precipitation in a mixture ratio of 1:15 (v/v, cleavage product: diethyl ether). Precipitation reaction was kept overnight at -80 °C and the cross-linkers were pelleted by centrifugation at 10,000 × g for 30 min at 4 °C. Diethyl ether was decanted, and pellet was dried and dissolved with dimethyl sulfoxide (DMSO) to yield an approximately 200 mM concentration of BDP-NHP cross-linkers. The purity of the cross-linkers was confirmed with direct infusion ESI-MS analysis. Cross-linkers were stored at -80 °C prior to use.

LC-MS ReACT analysis

A real-time informatics strategy (ReACT), previously described[1], was used to improve the throughput of cross-linked peptide analysis. In MS^1 scan events, ions of charge states ≥ 4+ were identified using FTICR mass analyzer with 50,000 resolving power. The most abundant ≥ 4+ ions were selected for MS^2 fragmentation analysis, with 4 m/z isolation width, 25 CID energy and FTICR scans of 50,000 resolving power. Automatic gain control (AGC) target values were set at 5×10^5 for MS^1 and MS^2. Since the D-P

bond of the cross-linkers is cleaved by lower energy CID during MS^2, the cross-linked peptide pair was released into two individual peptides (each carrying a stump modification on the labeled Lys residues, 197.0324 Da), and a reporter ion corresponding to BDP-NHP fragments (752.4051 Da). Thus, immediately after the MS^2 spectrum was collected, the masses of the top 200 most abundant peaks were examined to determine if any of the two peaks can be paired to reconstitute the mass equation (Eq.1) within 20 ppm mass errors (allowing two ^{13}C isotope offsets).

$$\text{Mass } [MS^1, \text{Precursor}] = \text{Summed Masses } [MS^2, \text{Reporter} + \text{Peak}_1 + \text{Peak}_2] \qquad \text{(Eq.1)}$$

If the paired peaks were identified, their m/z values were used for MS^3 peptide identification analysis, using 5 m/z isolation width, 35 CID energy and linear ion trap scans. In occasions that 2 or more pairs of peaks met the mass equation, the most abundant pair (with highest summed peak intensity) was selected for MS^3. Since 1+ peptides are generally difficult to fragment preventing sequence identification, the theoretical 2+ ion species for the observed 1+ ions were also targeted for MS^3 events. Thus, the MS^3 events consist of 4 scans, including 1+, Peak_1 and Peak_2; 2+, Peak_1 and Peak_2. Each identification cycle MS^1- MS^2- $4{\times}MS^3$ was repeated during the whole LC separation process (examples see Supplementary Fig. 2A). AGC target value 5×10^4 was for MS^3. The dynamic exclusion for the ≥ 4+ ions was enabled, which includes repeat count 2, repeat durations 15 s, exclusion list size 500, exclusion durations 60 s, and exclusion mass width low 1.0 and high 3.0.

Generating the stage 1 protein database with BDP-NHP cross-linkers in AB5075

The purified proteins from the cross-linked AB5075 cells, which was in the buffer of 50 mM NH_4HCO_3, 8 M Urea were diluted with 50 mM NH_4HCO_3 (pH 8.0) solution to reduce the urea concentration to less than 1 M. One milligram of the proteins were input and incubated with 100 µl of Monomeric Avidin UltraLink resin at room temperature for 30 min. The resins were washed 5 times with 50 mM NH_4HCO_3 (pH 8.0) solution before eluting the bound proteins with 8 M urea and 100 mM Tris (pH 8.0). The eluted proteins were protein candidates reactive to BDP-NHP cross-linkers in AB5075

cells. The eluted proteins were quantified with Bradford assays, reduced with 10 mM DTT, alkylated with 10 mM iodoacetamide, and digested with trypin in a 1:50 ratio at 37 °C overnight. Peptides were collected with C18 Sep-Pak cartridges, speedvac dry, and dissolved with 0.1% formic acid. Samples were either analyzed with a Velos-FT system coupled to a Waters nanoAcquity UPLC, or with a Q Exactive Plus system coupled to a Thermo EASY-nLC 1000. The C18 columns of a 3 cm trap column (5 µm, 200 Å) and a 60 cm analytical column (5 µm, 100 Å, Bruker) were used for both systems. For Velos-FT analysis, the LC separation gradients were 5% - 35% acetonitrile at flow rate of 300 nL/min for 120 min. MS^1 scan was acquired at 50, 000 resolution, and the top 20 most abundant peaks were selected for tandem mass spectrometry analysis with the ion-trap Velos. Velos-FT MS^2 settings included isolation width of 2.0, activation type of CID, and normalized collision energy of 35, activation Q of 0.25 and activation time of 10 ms. AGC target value was 5×10^5 for FT MS^1, and 1×10^4 for ion trap MS^2. Charge state exclusion was applied to singly charged ions and those with the undetermined charge states. Dynamic exclusion was enabled, including the settings of exclusion window of 0.5 m/z low to 1.5 m/z high, exclusion duration 30 s, list size of 500, and repeat count of 1.

For QE plus analysis, the LC separation gradients were 2% - 10% acetonitrile for 1 min, and 10% - 30% acetonitrile for 89 min, at flow rate of 300 nL/mim. QE plus settings include 70,000 resolution for MS^1 scan, and 17,500 resolution for MS^2 scans. AGC target value was 1×10^6 for MS^1, and 5×10^4 for MS^2. The top 20 most abundant ions were selected for fragmentation analysis, using 1.6 m/z isolation width, 25 NCE. Charge exclusion was applied to ions of undetermined charges, and ions with charges of 1, 6-8 and > 8. The dynamic exclusion was set to 30 s.

The raw data was converted to mzXML files with ReAdW (version 4.2.1). Velos-FT data was searched against the concatenated database containing forward and reverse sequences of AB5075 draft genome (total of 7626 entries) download from PGAT[19]. Searches were performed with Comet (version 2015.01 rev.02) including the parameters of 20 ppm precursor mass tolerance, ^{13}C offsets (-1/0/1/2/3) enabled, fragment bin tolerance 1.0005, fragment bin offset 0.4, variable modifications of methionine oxidation (15.9949 Da), fixed modification of cysteine carbamidomethylation (57.021464 Da). Only

fully tryptic peptide sequences were considered and up to 2 missed cleavages were allowed. For QE plus data, the same database and Comet parameter settings were used as Velos-FT data, except for that fragment bin tolerance 0.1 and fragment bin offset 0 were used for QE plus data. The false discovery rate for the peptide identification was determined with the target-decoy approach, and was controlled at 1%. A total of 1,741 proteins were identified with two or more unique peptides. The list of the proteins is provided in Supplementary Data 3.

Anti-HA co-immunoprecipitation (co-IP) with AB5075-pMMB-*oxa23* cells

For Oxa23-HA-Strep protein expression, AB5075-pMMB-*oxa23* strain was grown overnight in LB medium at 37 °C. Cells were diluted to OD_{600} = 0.6, and 0.3 mM Isopropyl β-D-1-thiogalactopyranoside (IPTG) was added to the LB medium. After adding the IPTG, cells were grown at room temperature for 18 h and cell pellets were harvested with centrifugation of 3,000 × g at 4 °C for 20 min.

Cell pellets of AB5075-pMMB-*oxa23* (or AB5075 WT control) cells were washed once with cell washing buffer [50 mM MOPS (pH 7.4), 150 mM NaCl], and were resuspended in a 1:10 ratio (gram: ml) with the ice-cold protein extraction buffer which contains 50 mM MOPS (pH 7.4), 150 mM NaCl, 1% Triton X-100 and EDTA-free protease inhibitor cocktail tablets (using the ratio of 10 ml buffer per tablet) (Roche, Basel, Switzerland). Cells were sonicated on-ice with a GE-130 ultrasonic processor using 50% amplitude and three cycles of 1 min duration. Protein extracts were centrifuged with 20,000 × g for 20 min at 4 °C to remove cell debris, and protein concentrations were determined with Bradford assays.

Monoclonal anti-HA agarose (Sigma) were washed twice with protein washing buffer [50 mM MOPS (pH 7.4), 150 mM NaCl, 0.1% Triton X-100]. Twenty microliters of anti-HA agarose and one milligram total proteins were input in each Co-IP assay. The mixtures were incubated at 4 °C overnight on a rotating shaker. Anti-HA agarose was collected with centrifugation of 3,000 × g for 3 min at 4 °C, and was washed four times with protein washing buffer. The co-IP proteins were eluted with 2 × Laemmli buffer (Bio-Rad) and boiling at 95 °C for 5 min.

Anti-HA co-IP with the BDP-NHP cross-linked AB5075-pMMB-*oxa23* cells

Since membrane protein structures are highly dependent upon the presence of the lipid membrane environment, it only stands to reason that cell lysis and extraction to remove proteins from this lipid environment causes membrane protein structural perturbations and loss of relevant binding partners in a way not equaled with soluble protein complexes. Thus, the relative success rate of co-IP of soluble protein interactions is indisputably higher compared to complexes that involve membrane proteins. One way to improve the Co-IP success rate for membrane proteins is to add a cross-linking step before the Co-IP analysis.

AB5075-pMMB-*oxa23* cells were grown in LB medium and Oxa23-HA-Strep protein expressions were induced with 0.3 mM IPTG at room temperature for 18 h. Cell pellets were collected with centrifugation of 3,000 × g for 20 min at 4 °C. Cell pellets were further washed three times with 6 ml of 5 mM Na_2HPO_4 (pH 7.4) and 150 mM NaCl buffer, and one time with 3 ml of 500 mM Na_2HPO_4 (pH 7.4) and 150 mM NaCl buffer, before resuspended in 500 mM Na_2HPO_4 (pH 7.4) and 150 mM NaCl buffer. Cross-linking reactions were performed with two doses of 5 mM BDP-NHP at room temperature, with a total reaction time of 1 h (30 min for each dose). Cells were mixed with 1,300 rpm on a thermomixer. Cross-linking reactions were ended by the addition of 20 mM Tris (pH 8.0). Cells were harvested with 3,000 × g centrifugation at 4 °C for 20 min.

Proteins were denatured and extracted from the cell pellets with 1.5 × Laemmli buffer and with 95 °C boiling for 5 min. Protein extracts were cooled down, and were sonicated for 30 s with 50% amplitude. Cell debris were removed with centrifugation of 20,000 × g for 20 min at room temperature. Protein extracts were diluted with the cross-linking IP buffer [50 mM MOPS (pH 7.4), 20 mM Tris, 150 mM NaCl, 0.05% Triton X-100] to SDS concentration less than 0.1%. Anti-HA agarose was added to the mixture and were incubated at room temperature for five hours. Anti-HA agarose was spun down with 3,000 × g for 3 min at 4 °C, and was washed four times with the cross-linking IP buffer. Proteins were eluted with 2 × Laemmli buffer (Bio-Rad) and boiling at 95 °C for 5 min.

SDS-PAGE and immunoblot analysis

Proteins were analyzed with the Bio-Rad Mini-PROTEAN system, and were transferred to Immobilon-FL PVDF membranes (Millipore, Billerica, MA, USA). Immunoblots for anti-OmpA, anti-CarO and anti-Oxa23 were performed with primary antibodies at 1: 5000 (vol: vol) ratio in PBST. The secondary antibodies of Goat anti-Rabbit IgG (H+L) conjugated with IRDye 800CW (LI-COR Biosciences, Lincoln, NE, USA) or Alexa Fluor 680 (ThermoFisher Scientific) were used. Immunoblots were imaged with the Odyssey Infrared Imaging System (LI-COR Bioscience).

Streptavidin IRdye (680 or 800 channels) (LI-COR Bioscience) was used in a 1:20,000 dilution ratio with PBST, and was imaged with LI-COR Odyssey.

Colony forming units (CFU) analysis

A. baumannii cells that underwent BDP-NHP or DMSO treatments were pelleted with 3, 000 × g centrifugation at 4 °C for 15 min. Cell pellets were resuspended with the sterile PBS solution (pH 7.4), and were divided into two halves. One half of the cells were serially diluted with PBS, and 5 µl of the cells at 10^5 and 10^6 dilutions were spotted onto LB agar plates. The CFU observed from the two dilutions were averaged, and was converted to CFU/ml based on the starting volume and the plated volume.

The other half of the cells were lysed with SDS. The extracted proteins were separated with SDS-PAGE, and were analyzed with Infrared dye-labeled Streptavidins (LI-COR Bioscience) or with Coomassie protein stain (Bio-Rad).

Oxa-23 enzyme activity assays

Full length sequence of *oxa-23* WT or site directed mutants were cloned into pET-28b vector using restriction sites XhoI and NcoI. WT and mutant constructs were transformed to BL21 (DE3) *E. coli* cells (New England Biolabs). To produce the recombinant Oxa-23 proteins, BL21 cells were grown in LB medium to OD_{600} = 0.6, and 0.3 mM IPTG was added to the cell culture, and cells were further incubated

at room temperature for 18 h with shaking. Cells were harvested by centrifugation and washed once with cell washing buffer [50 mM MOPS (pH 7.4), 150 mM NaCl], before resuspended in a 1:10 ratio (gram: ml) with the ice-cold protein extraction buffer which contains 50 mM MOPS (pH 7.4), 150 mM NaCl, and EDTA-free protease inhibitor cocktail tablets (using the ratio of 10 ml buffer per tablet). Cells were lysed by sonication with a GE-130 ultrasonic processor using 50% amplitude and four cycles of 1 min duration. Protein extracts were centrifuged with 10,000 × g for 20 min at 4 °C, and supernatants were collected for activity assays. The protein concentration was determined with Bradford assay. The expression of Oxa-23 recombinant proteins were quantified with SDS-PAGE coomassie gel stain and anti-Oxa-23 immunoblots.

Oxa-23 enzyme activity was determined with photospectrometric methods[4] in a 96-well plate format, using a SpectraMax 250 Microplate Reader and SoftMax Pro (version 2.04) (Molecular Devices, Sunnyvale, CA, USA). The detection wavelength and extinction coefficients (297 nM and 9,627 M^{-1} cm^{-1}) for imipenem and (298 nM and 12,668 M^{-1} cm^{-1}) for meropenem were used. One hundred microliter of substrates and one hundred microliter of cell lysate (approximately 0.5 mg total proteins) were mixed with shaking for 10 s, and the reduction of absorbance at OD_{297} or OD_{298} were monitored for 6 min at 22 °C with measuring time points of every 30 s. The collected quantitation points were subjected to linear regression analysis to determine the velocity of substrate hydrolysis with Oxa-23. Substrate concentrations for imipenem at 1.575 mM, 0.394 mM, 0.158 mM, 0.0788 mM, 0.0394 mM, 0.0158 mM were tested. Imipenem concentrations 0.394 mM and 0.158 mM showed good signal-to-noise enzyme activity detection, and were the main concentrations for activity comparison. Likewise, substrate concentrations for meropenem at 0.571 mM, 0.229 mM, 0.114 mM, 0.0571 mM, 0.0229 mM and 0.0114 mM were tested. Meropenem concentrations 0.114 mM and 0.057 mM yielded good signal-to-noise enzyme activity detection, and were the main concentrations for activity comparison. The hydrolysis activity of Oxa-23 was further normalized with the relative protein abundance level of Oxa-23 (WT or mutants) in *E. coli* lysate. The comparison of relative hydrolysis activity of Oxa-23 WT versus mutants

(Supplementary Fig. 10) was based on measurements of two biological replicates (i.e. two independent protein purification events). Three technical replicates were analyzed for each biological replicate.

RNA extraction and quantitative polymerase chain reaction (qPCR)

AB5075 wild type or mutant cells were cryoground using Mixer Mill MM 400 (Retsch, Haan, Germany). Total RNA was extracted from the frozen cell powder with RNeasy Mini Kit (Qiagen, Hilden, Germany) following QiaGen protocol, including the on-column DNase digestion step using Ambion Dnase. RNA concentration was measured with Nanodrop N1000 (ThermoFisher Scientific). The cDNA was synthesized with ImProm-II ReverseTranscription System kit (Promega) using the random hexamer primers, with the RNA template amount of 800 ng in each reaction.

The qPCR primers include a primer set for *16S RNA*[20] 5'CAGCTCGTGTCGTGAGATGT 3' (F), 5'CGTAAGGGCCATGATGACTT 3' (R), and two primer sets for oxa-23 gene 5' AAGCTTTCTGCAGTCCCAGT 3' (F), TCCAATTTCAGCATTACCGA 3' (R) that targets 370-474 bp position of *oxa-23* gene (corresponding to123-158 amino acid residues) and 5'TGGTTGGGCAATGGATATAA 3' (F), 5'TTTCTGACCGCATTTCCATA 3' (R) that targets 651-771 bp position of *oxa-23* gene (corresponding to 217-257 amino acid residues). The PCR amplification of cDNA template with these primer sets was verified with gel electrophoresis.

The qPCR was performed with a 7900HT Fast Real-Time PCR System (Applied Biosystems, Foster City, CA, USA) using SYBR green PCR master mix (Applied Biosystems), 200 nM primer concentration and a 1:10 dilution of the cDNA template. The thermal cycles were 50 °C 2 min, 95 °C 10 min, and 40 cycles of 15 seconds at 95 °C and 1 min at 56 °C. The qPCR data were analyzed with Sequence Detection Systems version 2.4 (Applied Biosystems). The critical threshold cycle (C_T) of *oxa-23* gene was referenced to *16S RNA* to obtain the ΔC_T for the wild type and the mutant cells. The $\Delta\Delta C_T$ of the mutant cells compared with wild type cells were used to estimate the relative abundance of *oxa-23* RNA in wild type versus mutant cells. Two technical replicates and three biological replicates for each mutant strain were analyzed.

Outer membrane protein fractionation with AB5075 cells

AB5075 wild type or mutant cells were grown overnight at 37 °C in LB medium. Cells were pelleted with centrifugation of 3,000 × g at 4 °C for 20 min. Cell pellets were resuspended with 10 ml cell washing buffer [50 mM MOPS (pH 7.4), 150 mM NaCl], and were pelleted again with centrifugation of 3,000 × g at 4 °C for 20 min. The cell pellets were resuspended in a 1:10 ratio (gram: ml) with the ice-cold Protein Extraction Buffer which contains 50 mM MOPS (pH 7.4), 150 mM NaCl, and EDTA-free protease inhibitor cocktail tablets (using the ratio of 20 ml buffer per tablet). Cells were cooled in ice for at least 15 min, before lysis with sonication on-ice with a GE-130 ultrasonic processor using 50% amplitude and four cycles of 1.5 min duration. Cell debris was removed by centrifugation of 10,000 × g for 20 min at 4 °C. The supernatants were further centrifuged at 100,000 × g for 1 h at 4 °C using Optima LE-80K ultracentrifuge and SW 41 Ti Rotor Swinging Bucket (Beckman Coulter, Brea, CA, USA). The collected supernatants are soluble protein-enriched fractions (Figs. 4D, 6D). The pellets were resuspended with 6 ml membrane solubilization buffer (100-fold volume excess), which contained 50 mM MOPS (pH 7.4), 150 mM NaCl, EDTA-free protease inhibitor cocktail tablets, 2% (v/v) Triton X-100 and 10 mM $MgCl_2$. The membrane solubilization buffer dissolved the cytoplasmic membranes, while did not dissolve the outer membranes[21, 22]. The solubilization mixtures were incubated on-ice for 30 min, and were centrifuged at 100,000 × g for 1 h at 4 °C. The supernatants were removed, and the pellets were washed with another 10 ml Protein Extraction Buffer (200-fold volume excess), and centrifuged at 100,000 × g for 50 min at 4 °C. The pellets were resuspended with 8 M urea in 50 mM NH_4HCO_3 (pH 8.0), vortexed, incubated at room temperature for 1 h, and were centrifuged at 20,000 × g at room temperature for 20 min. The collected supernatants were the outer membrane protein-enriched fractions (Figs. 4D, 6D). Protein concentrations for the soluble and outer membrane proteins enriched fractions were determined with Bradford assays. The extracted proteins were either denatured with 2 × Laemmli buffer for SDS-PAGE immunoblot analyses (Fig. 4D) or digested with trypsin for mass spectrometry analyses (Fig. 6D).

Parallel reaction monitoring (PRM) assays for Oxa-23 proteins

PRM analysis for label-free peptide quantitation was performed with a Q Exactive Plus system coupled to a Thermo EASY-nLC 1000. Peptides were separated with LC separation gradients 2% - 10% acetonitrile for 1 min, and 10% - 30% acetonitrile for 89 min, at flow rate of 300 nL/mim, using a 3 cm trap column packed with ReproSil-Pur 200 C18-AQ, 5 µm silica, and a 60 cm analytical column packed with ReproSil-Pur 120 C18-AQ, 5 µm silica (Dr. Maisch HPLC GmbH, Ammerbuch-Entringen, Germany).

PRM data were collected with QE plus settings 17,500 resolution for MS scans, AGC target value 5 $\times 10^4$, isolation window 1.6 m/z, maximum ion time 50 ms, fragmentation energy 25 NCE. The peptide target masses and the scheduled retention time were determined according to the peptide identification results with data-dependent acquisition analysis. Nine tryptic peptides of Oxa-23 could be quantified with PRM assays, three of which (NTSGVLVIQTDK, 48-59), (INLYGNALSR, 61-70) and (TGWAMDIKPQVGWLTGWVEQPDGK, 217-240) correspond to site directed mutation residues DKK60AAA and D238A, and the detection of these peptide was used for validation of specific mutants. The six other peptides (ANTEYVPASTFK, 71-82), (TDINEIFK, 95-102), (LSAVPVYQELAR, 125-136), (IGFGNAEIGQQVDNFWLVGPLK, 149-170), (VTPIQEVEFVSQLAHTQLPFSEK, 172-194) and (IVAFALNMEMR, 241-251) could be detected in both the wild type and mutant forms, and were used to quantify the relative abundance of Oxa-23 proteins.

The PRM data was analyzed with Skyline (version 3.5.0)[23] as previously described[24]. The log2 of peptide ratios were averaged to yield the protein-level ratios.

Supplementary References

1. Weisbrod CR, Chavez JD, Eng JK, Yang L, Zheng C, Bruce JE. In vivo protein interaction network identified with a novel real-time cross-linked peptide identification strategy. *Journal of proteome research* **12**, 1569-1579 (2013).

2. Navare AT, *et al.* Probing the protein interaction network of Pseudomonas aeruginosa cells by chemical cross-linking mass spectrometry. *Structure* **23**, 762-773 (2015).

3. Ishihama Y, *et al.* Exponentially modified protein abundance index (emPAI) for estimation of absolute protein amount in proteomics by the number of sequenced peptides per protein. *Mol Cell Proteomics* **4**, 1265-1272 (2005).

4. Smith CA, *et al.* Structural basis for carbapenemase activity of the OXA-23 beta-lactamase from Acinetobacter baumannii. *Chemistry & biology* **20**, 1107-1115 (2013).

5. Paetzel M, Danel F, de Castro L, Mosimann SC, Page MG, Strynadka NC. Crystal structure of the class D beta-lactamase OXA-10. *Nature structural biology* **7**, 918-925 (2000).

6. Chavez JD, Weisbrod CR, Zheng C, Eng JK, Bruce JE. Protein interactions, post-translational modifications and topologies in human cells. *Mol Cell Proteomics* **12**, 1451-1467 (2013).

7. Docquier JD, *et al.* Crystal structure of the narrow-spectrum OXA-46 class D beta-lactamase: relationship between active-site lysine carbamylation and inhibition by polycarboxylates. *Antimicrobial agents and chemotherapy* **54**, 2167-2174 (2010).

8. Park JS, *et al.* Mechanism of anchoring of OmpA protein to the cell wall peptidoglycan of the gram-negative bacterial outer membrane. *FASEB journal : official publication of the Federation of American Societies for Experimental Biology* **26**, 219-228 (2012).

9. Bushell SR, Mainprize IL, Wear MA, Lou H, Whitfield C, Naismith JH. Wzi is an outer membrane lectin that underpins group 1 capsule assembly in Escherichia coli. *Structure* **21**, 844-853 (2013).

10. Krivov GG, Shapovalov MV, Dunbrack RL, Jr. Improved prediction of protein side-chain conformations with SCWRL4. *Proteins* **77**, 778-795 (2009).

11. Pesce A, *et al.* Unique structural features of the monomeric Cu,Zn superoxide dismutase from Escherichia coli, revealed by X-ray crystallography. *Journal of molecular biology* **274**, 408-420 (1997).

12. Zurawski DV, *et al.* Genome sequences of four divergent multidrug-resistant Acinetobacter baumannii strains isolated from patients with sepsis or osteomyelitis. *J Bacteriol* **194**, 1619-1620 (2012).

13. Jacobs AC, *et al.* AB5075, a Highly Virulent Isolate of Acinetobacter baumannii, as a Model Strain for the Evaluation of Pathogenesis and Antimicrobial Treatments. *Mbio* **5**, e01076-01014 (2014).

14. Gallagher LA, *et al.* Resources for Genetic and Genomic Analysis of Emerging Pathogen Acinetobacter baumannii. *J Bacteriol* **197**, 2027-2035 (2015).

15. Kulasekara HD, Ventre I, Kulasekara BR, Lazdunski A, Filloux A, Lory S. A novel two-component system controls the expression of Pseudomonas aeruginosa fimbrial cup genes. *Molecular microbiology* **55**, 368-380 (2005).

16. Tang X, Munske GR, Siems WF, Bruce JE. Mass spectrometry identifiable cross-linking strategy for studying protein-protein interactions. *Analytical chemistry* **77**, 311-318 (2005).

17. Chavez JD, Weisbrod CR, Zheng C, Eng JK, Bruce JE. Protein interactions, post-translational modifications and topologies in human cells. *Mol Cell Proteomics* **12**, 1451-1467 (2013).

18. Bich C, Maedler S, Chiesa K, DeGiacomo F, Bogliotti N, Zenobi R. Reactivity and applications of new amine reactive cross-linkers for mass spectrometric detection of protein-protein complexes. *Analytical chemistry* **82**, 172-179 (2010).

19. Brittnacher MJ, Fong C, Hayden HS, Jacobs MA, Radey M, Rohmer L. PGAT: a multistrain analysis resource for microbial genomes. *Bioinformatics* **27**, 2429-2430 (2011).

20. Peleg AY, Adams J, Paterson DL. Tigecycline Efflux as a Mechanism for Nonsusceptibility in Acinetobacter baumannii. *Antimicrobial agents and chemotherapy* **51**, 2065-2069 (2007).

21. Filip C, Fletcher G, Wulff JL, Earhart CF. Solubilization of the cytoplasmic membrane of Escherichia coli by the ionic detergent sodium-lauryl sarcosinate. *J Bacteriol* **115**, 717-722 (1973).

22. Thein M, Sauer G, Paramasivam N, Grin I, Linke D. Efficient subfractionation of gram-negative bacteria for proteomics studies. *Journal of proteome research* **9**, 6135-6147 (2010).

23. MacLean B, *et al.* Skyline: an open source document editor for creating and analyzing targeted proteomics experiments. *Bioinformatics* **26**, 966-968 (2010).

24. Wu X, *et al*. Dynamic proteome response of Pseudomonas aeruginosa to tobramycin antibiotic treatment. *Mol Cell Proteomics*, (2015).
